# Mesothelial cells promote peritoneal invasion and metastasis of ascites-derived ovarian cancer cells through spheroid formation

**DOI:** 10.1126/sciadv.adu5944

**Published:** 2026-02-06

**Authors:** Kaname Uno, Masato Yoshihara, Yoshihiko Yamakita, Kazuhisa Kitami, Shohei Iyoshi, Mai Sugiyama, Yoshihiro Koya, Tomihiro Kanayama, Haruhito Sahara, Satoshi Nomura, Kazumasa Mogi, Emiri Miyamoto, Hiroki Fujimoto, Kosuke Yoshida, Satoshi Tamauchi, Akira Yokoi, Nobuhisa Yoshikawa, Kaoru Niimi, Yukihiro Shiraki, Jonas Sjölund, Hidenori Oguchi, Kristian Pietras, Atsushi Enomoto, Akihiro Nawa, Hiroyuki Tomita, Hiroaki Kajiyama

**Affiliations:** ^1^ Department of Obstetrics and Gynecology, Nagoya University Graduate School of Medicine, Nagoya, Japan.; ^2^ Division of Clinical Genetics, Department of Laboratory Medicine, Lund University, Lund, Sweden.; ^3^ Division of Oncology, Department of Clinical Sciences, Lund University, Lund, Sweden.; ^4^ Department of Obstetrics and Gynecology, TOYOTA Memorial Hospital, Toyota, Japan.; ^5^ Bell Research Center, Department of Obstetrics and Gynecology Collaborative Research, Nagoya University Graduate School of Medicine, Nagoya, Japan.; ^6^ Department of Tumor Pathology, Graduate School of Medicine, Gifu University, Gifu, Japan.; ^7^ Department of Pathology, TOYOTA Memorial Hospital, Toyota, Japan.; ^8^ Discipline of Obstetrics and Gynecology, Adelaide Medical School, Robinson Research Institute, University of Adelaide, Adelaide, Australia.; ^9^ Department of Pathology, Graduate School of Medicine, Nagoya University, Nagoya, Japan.; ^10^ Department of Laboratory Medicine, Division of Translational Cancer Research, Lund University Cancer Center, Lund University, Lund, Sweden.; ^11^ SciLifeLab, Department of Laboratory Medicine, Lund University, Lund, Sweden.

## Abstract

Patients with epithelial ovarian cancer (EOC) are often diagnosed with peritoneal metastasis and ascites, the accumulation of intraperitoneal fluid containing nonmalignant cells. However, the interactions between EOC and nonmalignant cells before peritoneal metastasis remain unclear. To investigate this, whole EOC spheroids were observed using a multiphoton microscope, and their invasion ability was assessed. Mesothelial cells were identified as notable components of ascites through morphological assessment, immunohistochemical/immunofluorescence staining, and single-cell RNA sequencing analyses. Almost all EOC cells were spheroids, with 60% containing mesothelial cells. EOC cells quickly generate aggregated spheroids with mesothelial cells, and these aggregated cancer-mesothelial spheroids (ACMSs) invade collagen or mesothelial layers. Mesothelial cells forming ACMSs initiated the invasion. RNA sequencing analysis revealed marked RNA expression changes in mesothelial cells, whereas the changes in EOC cells were minor. Transforming growth factor–β1–stimulated mesothelial cells showed increased invadopodium formation along with fascin-1 up-regulation. These findings suggest that EOC cells alter mesothelial cells through ACMSs, thereby elucidating the rapid spread of EOC in the abdominal cavity.

## Teaser

Ovarian cancer cells form spheroids with surrounding mesothelial cells in the ascites, altering them to facilitate metastasis.

## INTRODUCTION

Epithelial ovarian cancer (EOC) is a fatal gynecological cancer (*1*). More than 75% of patients with EOC are diagnosed at an advanced stage because there is currently no effective screening process and there are no specific symptoms in the initial stages (*2*). Many clinical trials have attempted to detect early-stage EOC using frequent ultrasound examinations and tumor markers, including CA125. However, these interventions did not improve patient prognosis (*3*). Early detection primarily fails because EOC can almost immediately metastasize to other organs inside the abdominal cavity once it develops in the ovary or fallopian tube (*4*). Induction of metastasis via ascites (abdominal fluid) is known as trans-coelomic dissemination, which is a unique characteristic of EOC. Although a previous model described EOC cells as traveling through the abdominal cavity using ascites fluid as a single cell (*5*, *6*), a more recent study provided evidence indicating that EOC cells can form groups referred to as cancer spheroids (*7*, *8*). Several studies have suggested that the formation of spheroids may potentially prevent programmed cell death induced by detachment from the extracellular matrix (ECM), which is termed anoikis (*8*, *9*). Research has also shown that EOC cells form spheroids with both cancerous and nonmalignant cells in ascites. These EOC spheroids comprise macrophages (*10*), mesothelial cells (*11*, *12*), and fibroblasts (*13*). However, the mechanism by which EOC cells survive in ascites, the induction of peritoneal disseminations through the interaction with the tumor microenvironment in ascites, and the advantages of EOC cells that form heterocellular spheroids with surrounding cells have not yet been fully elucidated.

The surface of the abdominal cavity is covered with a single layer of mesothelial cells, which has an area large enough to cover the entire human body (*14*). The interior of the cavity is filled with ascites fluid, which acts as a lubricant for the internal organs. The fluid comprises various cell types, including macrophages, mesothelial cells, lymphocytes, and neutrophils (*7*), creating a unique microenvironment. Mesothelial cells are the predominant cell type, accounting for 30% of the ascites (*6*, *15*, *16*). Mesothelial cells exhibit both epithelial and mesenchymal characteristics depending on their surroundings (*17**–**20*) and help to maintain homeostatic stability in the abdominal cavity (*21**–**23*). Mesothelial cells can markedly alter their morphology, especially in response to inflammation in the abdominal cavity (*24**–**26*). Reactive mesothelial cells have large nuclei, high chromatin content, and irregularly shaped nuclear membranes. Occasionally, their morphology is similar to that of malignant cells (*27*, *28*). Moreover, the ancestors of EOC and mesothelial cells are similar (*16*, *21*, *29*); consequently, they share several markers, which can pose challenges in distinguishing between the two using a single marker (*27*, *30*). Although mesothelial cells may play an important role in EOC progression, few studies have assessed mesothelial cells in relation to peritoneal disseminations (*19*, *31*); therefore, their roles in this process are currently unclear.

When EOC cells metastasize to other organs, they are initially transported by the ascites and must attach to the mesothelial cell layer to reach the basement membranes below (*6*, *7*). Once they reach the basement membrane, they use matrix metalloproteinases (MMPs) to degrade the ECM and dig into the connective tissues and stroma (*32*, *33*). Cancer cells use actin-based cellular instruments, such as invadopodia, filopodia, and lamellipodia (*34*), during these processes. A report on gastric cancer indicated that mesothelial cells form invadopodia and use them as a spearhead to invade the stroma (*35*). However, the mechanism by which cancer cells modify mesothelial cells, which are considered protective barriers of the intraperitoneal cavity, into invasive entities capable of metastasizing to other internal organs remains unclear.

Growth factors and cytokines in cancerous ascites alter mesothelial cells to cancer-associated mesothelial cells (*14*, *19*, *31*). We suspect that direct contact between cancerous and mesothelial cells in ascites is necessary for mesothelial cell transformation. In this study, we hypothesized that EOC cells interact with surrounding nonmalignant cells in the ascites before inducing peritoneal metastasis and that EOC cells can survive and induce peritoneal dissemination via the formation of heterocellular spheroids with unique cellular components in the ascites.

## RESULTS

### Ovarian cancer cells are present as spheroids, and mesothelial cells are a major component of ascites

**Fig. 1.**
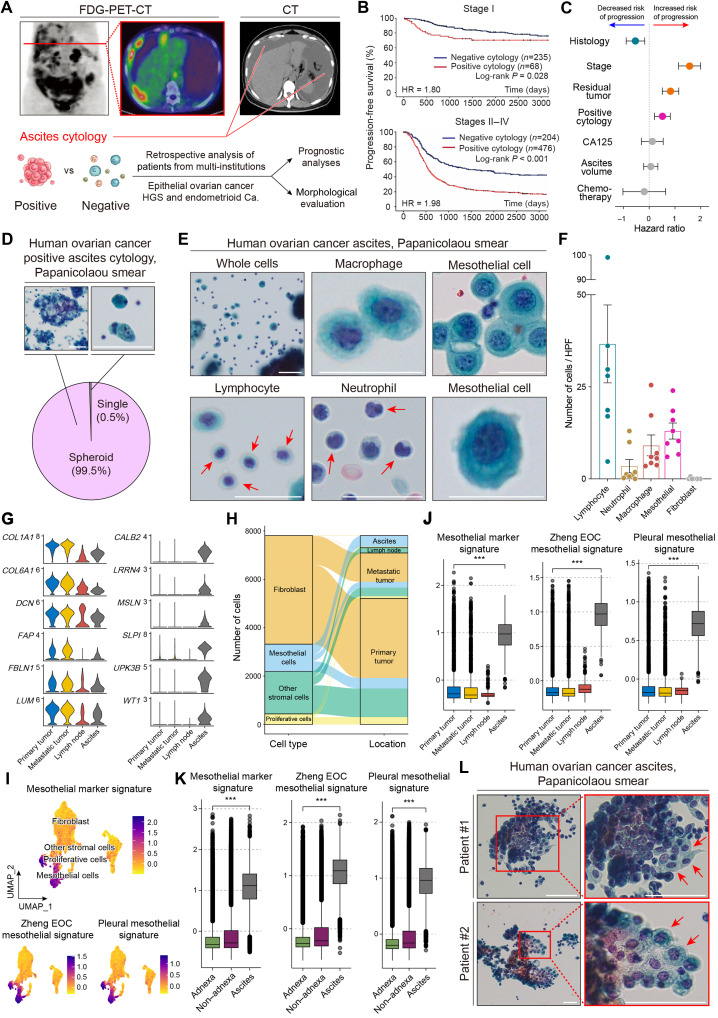
Ovarian cancer cells are present as spheroids, and mesothelial cells are major components of ascites. (**A**) PET-CT (positron emission tomography-computed tomography) and CT images from a typical patient with advanced ovarian cancer. (**B**) Kaplan-Meier analyses for progression-free survival in patients with stage I (*n* = 303) and stage II/III/IV (*n* = 680) ovarian cancer. Positive cytological results indicate the existence of EOC cells in ascites. (**C**) Forest plot of multiple regression analysis related to progression-free survival. (**D**) Pie chart showing the proportion of spheroids or single cells in ascites with cytology slides from ascites with Papanicolaou staining. Scale bars, 50 μm. (**E**) Representative images of nonmalignant cells in ascites with Papanicolaou staining, which present as single cells. Scale bars, 25 μm. (**F**) The bar figure shows frequencies of the nonmalignant cells in ascites on the basis of their cellular morphology in the high-power fields. (**G**) Violin plots showing the expression of fibroblast and mesothelial markers in the dataset (*40*) depending on tumor location. (**H**) Alluvial plot illustrating the number of cells and proportion of stromal cell components depending on tumor location in the Zheng *et al.* scRNA-seq dataset (*40*). (**I**) UMAP plots of the stromal cell clusters of the Zheng *et al.* EOC dataset (*40*) showing the expression of the three different mesothelial signatures. (**J**) Box plots showing the expression of the three different mesothelial signatures in the Zheng *et al.* EOC dataset (*40*) depending on tumor locations. (**K**) Box plots showing the expression of the three different mesothelial signatures in another EOC scRNA-seq dataset (*37*) depending on tumor sites. (**L**) Representative images of suspected heterocellular spheroids composed of different cell types. Scale bars, 50 μm. FDG, fluorodeoxyglucose; Ca., carcinoma; HPF, high-power field. ***P < 0.001.

Most patients with EOC are diagnosed at an advanced stage and present with malignant ascites containing numerous EOC cells (Fig. 1A). To understand the importance of malignant ascites, we retrospectively collected multi-institutional data from 1856 patients with ovarian cancer from the Tokai Ovarian Cancer Group. Among these patients, 983 were recruited with high-grade serous ovarian carcinoma (HGSOC) or endometrioid carcinoma, and their prognoses were compared on the basis of the presence (positive) or absence (negative) of malignant cells in their ascites. Positive cytology resulted in significantly shorter progression-free survival in stage I disease [hazard ratio (HR) = 1.80] (Fig. 1B). Moreover, patients with stage >I disease who already had metastases had significantly poorer prognoses when EOC cells were identified in the ascites (HR = 1.98). Positive cytology was also associated with overall patient survival in both groups (fig. S1A). Forest plot analysis revealed that positive cytology was significantly associated with shorter progression-free survival in the multivariate analysis (Fig. 1C; HR: 1.430; 95% confidential interval: 1.157 to 1.766). The patient backgrounds are summarized in table S1.

EOC cells in the ascites were further examined to evaluate their structures. Although previous studies have mentioned that EOC cells occur as “single cells,” almost all (99.5%) of the EOC cells identified in this study were spheroids (Fig. 1D). The sizes and constituents of the spheroids varied among patients (fig. S1B). Nonmalignant cells were also identified around the cancer spheroids, most of which were single cells. Macrophages, mesothelial cells, neutrophils, and lymphocytes constituted the major cellular components of the ascites morphological signatures (Fig. 1E). The proportion of lymphocytes varied according to the patient, and no fibroblast-like spindle cells were identified in the ascites (Fig. 1F).

Mesothelial cells, the major cellular component in ascites (*6*, *15*), are considered complex because they can alter their morphology and protein expression depending on the surrounding circumstances (*17*, *18*). They can exhibit morphologies similar to cancer cells under inflammation, during which they are called activated mesothelial cells (fig. S1C). For example, their morphology changed from a cobblestone appearance to a mesenchymal-like phenotype, and calretinin levels significantly decreased when mesothelial cells were stimulated with transforming growth factor–β1 (TGF-β1) (fig. S1D). Although mesothelial cells are the primary and essential components of ascites, Izar *et al.* (*36*) and Vázquez-García *et al.* (*37*) did not consider them in their studies using single-cell RNA sequencing (scRNA-seq) analysis. To address this issue, we reanalyzed the data of Izar *et al.* (*36*), reproduced their clustering (fig. S2A), and checked their four-fibroblast clusters for the RNA expression levels of fibroblast, mesothelial, and ovarian cancer cell markers (fig. S2B). Most of the cells in clusters 6 to 9, which had been annotated as “fibroblasts” by Izar *et al.* (*36*), did not only express fibroblast markers (e.g., *COL1A1*, *DCN*, and *ACTA2*), but they also expressed mesothelial-specific markers (e.g., *DES*, *LRRN4*, *UPK3B*, *MSLN*, and *WT1*) (fig. S2C). Moreover, the cells in clusters 6 to 9 also expressed keratin 8 (KRT8) and KRT18, which are normally negative in fibroblasts (*38*, *39*). The cells in the cluster showed low or absent expression of cancer cell–specific markers (e.g., *PAX8*, *TP53*, and *EPCAM*) (fig. S2D). These results indicate that the “fibroblasts” were a mix of fibroblasts and mesothelial cells. Furthermore, their study included only ascites samples from a small number of patients (*n* = 6), making it impossible to perform comparative analysis among different tumor sites, e.g., primary tumors, metastases, and ascites.

To strengthen the notion that mesothelial cells are the principal stromal cell type in ascites as compared to other tumor sites, we used a more recent scRNA-seq dataset from 14 patients with multiple samples (*40*). This study showed that desmin-positive mesothelial cells are the dominate subpopulation of stromal cells in ascites as compared to other tumor locations. We reproduced the exact clustering (fig. S3A) and show the stromal expression levels depending on the tumor site (primary, metastasis, lymph node, and ascites). As expected, only the stromal cells in ascites and mesothelial cells showed high expression levels of mesothelial-specific markers (*LRRN4*, *KRT8*, *KRT18*, *MSLN*, *UPK3B*, and *WT1*), and they were also positive for fibroblast markers (e.g., *COL1A1*, *FAP*, and *PDGFRA*) (Fig. 1G and fig. S3, B and C). Stromal cells at other sites were almost negative for the mesothelial markers. An analysis of the cell type composition of various tumor locations, the alluvial plot, showed that >90% of the stromal cells in the ascites were mesothelial cells (Fig. 1H). In addition to the expression of the individual mesothelial and fibroblastic marker genes, we also created signatures of the six prototypical marker genes of each cell type (Fig. 1G), as well as signatures comprising the top 30 differentially expressed genes between the fibroblast and mesothelial cell clusters of the Zheng *et al.* EOC dataset (*40*) and a human parietal pleural scRNA-seq dataset (fig. S3D) (*41*). The expression analyses of the three different signatures of each cell type showed that the signatures were highly specific toward fibroblasts and mesothelial cells (Fig. 1I and fig. S3, E and F) and that the mesothelial signatures were expressed at higher levels in the ascites samples compared with the primary and metastasis tumors (Fig. 1J). In contrast, the fibroblast gene signatures were expressed at lower levels in the ascites samples than in the primary and metastasis tumor samples (fig. S3G).

Furthermore, we downloaded and reanalyzed additional EOC scRNA-seq datasets (*37*) to validate and compare cellular components in different tumor locations. This study included 160 adnexa, non-adnexa, and ascites samples. It should be noted that the original cell type annotation failed to identify mesothelial cells among their cell clusters. We recreated the uniform manifold approximation and projection (UMAP) (fig. S3H) and compared RNA expression levels in the cluster annotated as fibroblasts using the six archetypical marker genes of each cell type, the three fibroblast and mesothelial signatures across the various tumor sampling sites. All three mesothelial cell signatures were expressed at higher levels in the ascites than in the adnexa and non-adnexa samples (Fig. 1K and fig. S3I). In contrast, the fibroblast signatures were lower in the ascites (fig. S3J). These results were in accordance with our morphological assessments using clinical cytology slides (Fig. 1F) and strongly support the significant presence of mesothelial cells in ascites.

When assessing EOC spheroids in ascites, we realized that there were several spheroids that were composed of at least two cell types because of the differences in the morphologies of the cells. EOC cells had large and irregularly shaped nuclei with high chromatin concentrations. In contrast, cells accompanied by EOC spheroids had small and smooth nuclei without chromatin concentrations and lower nucleus/cytoplasm ratios than EOC cells (Fig. 1L, red arrows). These morphologies are similar to those of mesothelial cells. Immunohistochemistry (IHC) analysis of the cell block samples from ascites revealed that several cells within the cancer spheroids were negative for EPCAM (epithelial cell adhesion molecule; epithelial marker) and positive for calretinin or Hector Battifora mesothelial 1 (HBME1; for detecting mesothelial cells) (fig. S3K). Calretinin and Wilms tumor 1 (WT1)–positive mesothelial cells were negative for paired box gene 8 (PAX8), claudin-4, and CD8 (fig. S3L). On the basis of these results, we hypothesized that EOC spheroids were composed of EOC and mesothelial cells and that these heterocellular spheroids have survival advantages in ascites and induced peritoneal metastasis.

### HBME1 can be used to distinguish mesothelial cells from the cancer spheroids

**Fig. 2.**
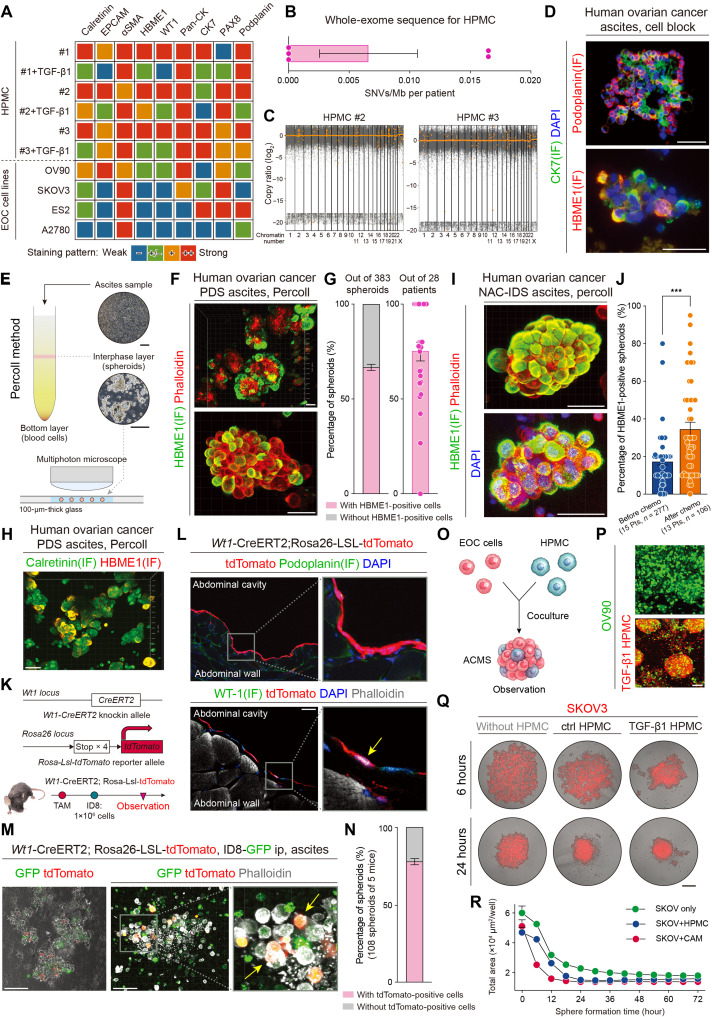
HBME1 can be used to distinguish mesothelial cells, which are present in ovarian cancer spheroids. (**A**) Heatmap showing the IHC staining pattern in each marker. (**B** and **C**) Whole-exome sequencing of the HPMCs. (B) The single-nucleotide variants (SNVs) per megabase and (C) copy number variations (CNVs) were very small. (**D**) Immunofluorescence (IF) image of the cell block samples with CK7 (for EOC cells) and HBME1 or podoplanin (for mesothelial cells). Scale bars, 100 μm. (**E**) Scheme depicting and observing the spheroids in the ascites using the Percoll method and a multiphoton microscope. Scale bars, 200 μm. (**F**) Representative images of spheroids with HBME1 (green) and phalloidin (red) staining using a multiphoton microscope. Scale bars, 50 μm. (**G**) Box plots showing the percentage of spheroids with and without HBME1-positive mesothelial cells. (**H**) Images of spheroids with HBME1 and calretinin staining. Scale bar, 50 μm. (**I**) Representative images of spheroids with HBME1 (green) after chemotherapy. Scale bars, 50 μm. (**J**) Difference in the percentage of HBME1-positive cells in spheroids collected before [PDS (primary debulking surgery)] and after [NAC-IDS (neoadjuvant chemotherapy-interval debulking surgery)] chemotherapy. ***P < 0.001. (**K**) Schematic diagram showing the Wt1-CreERT2; tdTomato mouse model. (**L**) Abdominal cavity 4 weeks after tamoxifen induction. Scale bar, 15 μm. (**M**) Representative images of spheroids from malignant ascites. Scale bars, 50 μm. (**N**) Bar graph showing that more than 75% of these spheroids were composed of mesothelial cells. (**O**) Schematic image of spheroid formation. (**P**) Images of spheroids after being in the rounding incubation system. Scale bar, 200 μm. (**Q** and **R**) Formation of spheroids with different incubation times and sphere-forming curves analyzed using the time-lapse imaging system. (i) SKOV3 only, (ii) with HPMCs, and (iii) with TGF-β1–stimulated HPMCs. Scale bar, 200 μm. TAM, tamoxifen; ip, intraperitoneally.

Mesothelial cells have the similar ancestor to the EOC cells, and they sometimes share the same markers (*16*, *30*, *36*). When EOC spheroids in the ascites were stained with calretinin or αSMA (α–smooth muscle actin) to detect mesothelial cells after incubation on a glass slide, both mesothelial and EOC cells were positive for the markers (fig. S4A). These markers could not be used to detect mesothelial cells. Consequently, to find a suitable marker to divide mesothelial cells from EOC cells, three human primary mesothelial cells (HPMCs, nos. 1 to 3), HPMCs stimulated with TGF-β1, and four EOC cell lines (OV90, SKOV3, ES2, and A2780) were used to identify a suitable marker for distinguishing mesothelial cells from EOC cells. These cells were stained with nine well-known clinically used markers to detect EOC or mesothelial cells. Expectedly, calretinin was positive in HPMCs as well as in OV90 cells. In addition, the expression was decreased or negative in TGF-β1–stimulated HPMCs (fig. S4B). The staining patterns of HPMCs and TGF-β1–stimulated HPMCs differed, and mesothelial cells showed both epithelial and stromal characteristics. HBME1 was considered the most suitable marker for distinguishing mesothelial cells from EOC cells (Fig. 2A and fig. S4B). Considering the heterogeneity of ovarian cancer cells, we assessed HBME1 staining in primary ovarian cancer tumor. Among 14 HGSOC cases, no cancer cells showed positive staining for HBME1 (fig. S4C). HBME1 was considered to detect mesothelial cells in EOC spheroids in subsequent experiments.

Before performing subsequent experiments, we analyzed whole-exome sequencing and immunofluorescence (IF) staining in our HPMCs to confirm that they were not from cancer cells. We analyzed whether HPMCs had *TP53* mutation or high-copy-number aberrations, which are the HGSOC-specific mutation and genetic character (*42*, *43*). Whole-exome sequencing revealed that our HPMCs exhibited minimal genomic aberrations, a low frequency of single-nucleotide variants (SNVs) per megabase, and no mutations in ovarian cancer–specific genes, including *TP53*, *BRCA1/2*, *ARID1A*, *PIC3CA*, and *KRAS* (Fig. 2, B and C, and fig. S5, A and B). In contrast, EOC typically displays a high number of copy number aberrations, and EOC cell lines commonly harbor these mutations (*44**–**46*). We also assessed p53 staining of HPMCs and compared the staining pattern with ovarian cancer cell lines. Kuramochi, OVCAR3, and ES2 were strongly positive for p53 staining, whereas all HPMCs showed a wild-type p53 staining pattern (~5% of mesothelial cells were positive with low intensity; fig. S5C). Moreover, our HPMCs were isolated from the omentum whose patients had borderline tumor or stage IA carcinoma. These results indicated that our HPMCs were not from cancer cells.

Then, we checked that our HPMCs had a similar staining pattern to mesothelial cells detected using the scRNA-seq dataset in ascites. HPMCs were stained with some fibroblast- and mesothelial-specific markers, and the staining pattern was compared with the mesothelial cell line LP9 and fibroblast cell line BJ. All the HPMCs, LP9, and BJ were positive for fibroblast markers [PDGFRα (platelet-derived growth factor receptor α) and vimentin]. HPMCs and LP9 were also positive for mesothelial-specific markers leucine-rich repeat neuronal 4 (LRRN4), desmin, mesothelin (MSLN), KRT8, KRT18, and uroplakin 3B (UPK3B), whereas BJ was negative for these staining. Figure S6 summarizes these staining results. The immunofluorescence results were in accordance with the scRNA-seq dataset shown in Fig. 1 (G to K) and figs. S2 and S3, and our HPMCs had the typical mesothelial characters in ascites.

### Mesothelial cells are present in ovarian cancer spheroids, forming ACMSs, in different perspectives

Cell blocks were created from prospectively collected clinical ascites to detect mesothelial cells in EOC spheroids. IHC revealed HBME1-, podoplanin-, and calretinin-positive cells in the EOC spheroids (fig. S7A). Double immunofluorescence staining with CK7 or PAX8 (for EOC cells) and podoplanin or HBME1 (for mesothelial cells) revealed that mesothelial cells formed spheroids with EOC cells (Fig. 2D and fig. S7B). HBME1-expressing cells were also positive for calretinin (fig. S7B). Although staining cell block samples showed mesothelial cells in spheroids, analyzing a sufficient quantity of spheroids was difficult. Thus, the Percoll method was used to remove most red blood cells from the bottom layer, and spheroids were collected from the interphase layer (Fig. 2E) (*47*). A multiphoton microscope was used to observe whole-spheroid structures because its observations are deeper than those of an ordinary laser confocal microscope. Spheroids were inserted in a 100-μm-wide space formed by glass plates that were 100 μm thick before observation (Fig. 2E). The multiphoton microscope could observe whole spheroids, and HBME1 clearly distinguished mesothelial cells within EOC spheroids in the three-dimensional (3D) images (Fig. 2F, fig. S7C, and movie S1), implying the potential use of HBME1 as a marker for mesothelial cells. Overall, 383 EOC spheroids were observed in 28 patients with HGSOC, of whom 13 had received chemotherapy before surgery and 15 were chemotherapy naïve. The results revealed that >60% were composed of mesothelial cells, and >50% cancer spheroids were composed of HBME1-positive mesothelial cells in most patients with HGSOC (Fig. 2G). We also attempted to detect mesothelial cells using calretinin, WT1, and podoplanin. However, some EOC cells were positive for these markers (Fig. 2H and fig. S7D). Both EOC and mesothelial cells were positive for calretinin and WT1. Notably, when EOC spheroids were compared before and after chemotherapy, the proportion of HBME1-positive mesothelial cells was significantly higher in patients who received chemotherapy before surgery than in those who did not (average HBME1-positive cells: before, 13.8%, *n* = 277 versus after, 29.8%, *n* = 106, *P* < 0.001; Fig. 2, I and J). Some spheroids observed after chemotherapy showed cancer cells located within HBME1-positive cells (suggesting that mesothelial cells may provide protection to the cancer cells; Fig. 2I and movie S2).

Last, to strengthen our hypothesis that mesothelial cells are present in cancer spheroids, we created a Wt1-CreERT2; tdTomato reporter mouse model. The Wt1-CreERT2 mouse lines combined with fluorescent reporter systems enable effective lineage tracing of mesothelial cells in various organs, including the peritoneum, heart, and kidneys (*48*). In this model, Wt1-positive cells expressed tdTomato fluorescence protein expression after tamoxifen induction (Fig. 2K). In the abdominal cavity, we confirmed that only the mesothelial layer was positive for tomato expression 4 weeks after tamoxifen induction, and these cells were positive for both WT1 and Tomato on the peritoneal membrane and surface of omentum (Fig. 2L and fig. S7E). Green fluorescent protein (GFP)–labeled ID8 mouse EOC cells were injected into the abdominal cavity. Four weeks after injection, we isolated cancer spheroids from malignant ascites. More than 70% of these spheroids comprised both GFP-positive ID8 and tdTomato-positive mesothelial cells (Fig. 2, M and N).

To determine how EOC cells form spheroids with mesothelial cells in ascites, we created EOC spheroids with and without mesothelial cells (Fig. 2O). In the rounding incubation system, which reflects ascites movement, OV90 did not form aggregated spheroids. In contrast, OV90 formed aggregated spheroids with mesothelial cells. OV90 and mesothelial cells were randomly present in these aggregated spheroids (Fig. 2P). We continually observed sphere formation every hour for 72 hours in 96-well low-attachment round plates, which promoted the formation of a spheroid-like structure. OV90 or SKOV3 rapidly formed aggregated spheroids with mesothelial cells, especially with mesothelial cells stimulated by TGF-β1, compared with spheroids containing only EOC cells (Fig. 2Q and fig. S7F). The time required for spheroid formation was 1.5 to 6 times shorter when EOC cells formed spheroids with mesothelial cells. Spheroids with mesothelial cells were 1.5 to 2 times more aggregated than spheroids with only EOC cells (SKOV3; 3.18 × 10^5^ versus 1.59 × 10^5^ μm^2^, *P* < 0.001) (Fig. 2R and fig. S7G). Moreover, spheroids composed of only EOC cells were easily broken, whereas EOC spheroids with mesothelial cells were more strongly connected (movies S3 and S4). These results revealed that cancer spheroids comprise EOC and mesothelial cells. EOC cells can form compact spheroids with surrounding mesothelial cells, which are termed as aggregated cancer-mesothelial spheroids (ACMSs), in ascites immediately after detachment from the primary site.

### ACMSs are highly invasive into collagen and mesothelial layers

**Fig. 3.**
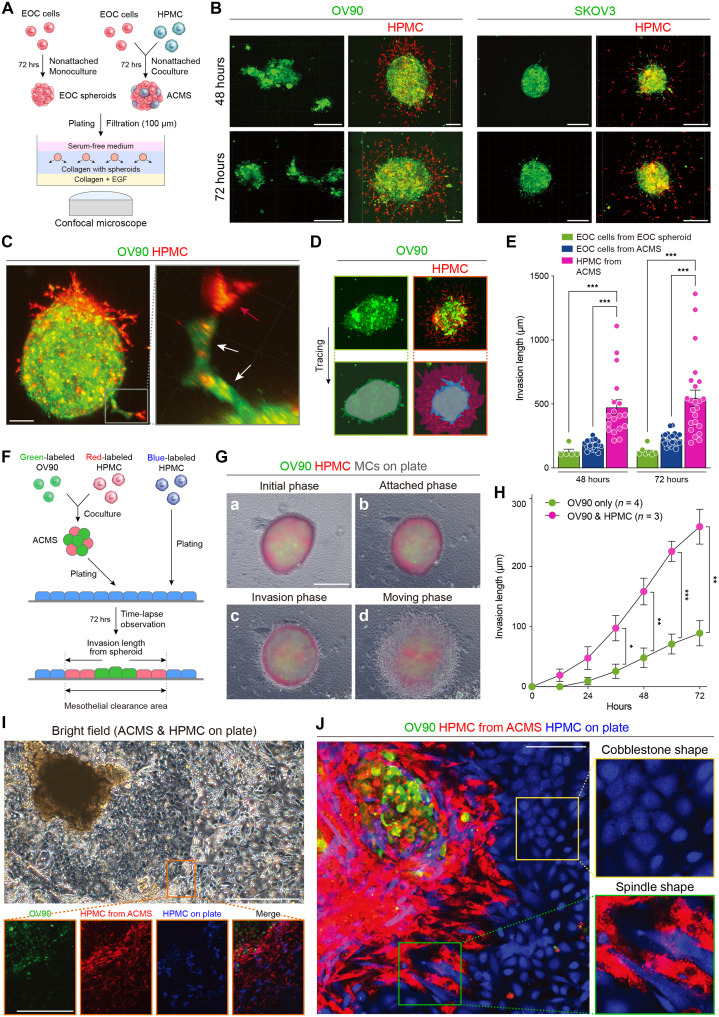
ACMS has an aggressive invasion ability, and mesothelial cells inside ACMSs invade the collagen and mesothelial layers first. (**A**) Schematic illustration of the collagen invasion assay. (**B**) Representative images of spheroid collagen invasion. Scale bars, 200 μm. (**C**) Different angles of 3D collagen spheroid invasion. Red-stained mesothelial cells invaded the collagen first (red arrow), followed by green-stained OV90 cells, which used the same route (white arrow). Scale bar, 50 μm. (**D**) Scheme showing spheroid invasion areas into the collagen layer and original spheroid area. (**E**) Bar plots of the spheroid invasion areas. The invasion area of ACMS was significantly larger compared with spheroids with only EOC cells (125 versus 480%, extended from the original spheroid area at 48 hours). The invasion area of EOC cells from ACMSs was significantly larger compared to those with only EOC spheroids (128% versus 223%, at 72 hours). Each dot represents each experiment. (**F**) Scheme of the mesothelial clearance assay. (**G**) Representative images of several different ACMS invasion timings into the mesothelial layer. (a) Initial phase, (b) attached phase, (c) invasion phase, and (d) moving phase. Scale bar, 200 μm. (**H**) The mesothelial clearance area was significantly larger for ACMSs than that of spheroids with only OV90. (**I**) Representative images of the invasion front from ACMSs into the mesothelial layer. Scale bar, 200 μm. (**J**) High-magnification images of the ACMS and mesothelial layer invasion front. The blue-stained mesothelial cells close to the EOC invasion area were of a spindle-like shape, but originally, they were cobblestone shaped. The morphology was similar to that of red-stained mesothelial cells that interacted with EOC cells in ACMSs. Scale bar, 100 μm. *P < 0.05, **P < 0.01, and ***P < 0.001.

To induce peritoneal metastasis, EOC cells must attach to and invade the mesothelial layer. Spheroids are important structures that induce peritoneal metastases (*49*). We compared the invasion ability of spheroids containing only EOC cells (homotypic) and ACMSs (heterotypic). Green-stained EOC cells (OV90 or SKOV3) formed spheroids with or without red-stained mesothelial cells. They were embedded in the collagen layer, and invading cells were observed after 48 and 72 hours (Fig. 3A). When spheroids were composed of only EOC cells (homotypic), a few EOC cells invaded the collagen layer, and the invasion depth was <100 μm. In contrast, the red-stained mesothelial cells from ACMSs (heterotypic) aggressively invaded the collagen layer at a depth of >300 μm (Fig. 3B). Notably, the red-stained mesothelial cells invaded the collagen layer first, followed by green-stained EOC cells (Fig. 3C). These results remained the same even when the staining colors were changed (fig. S8A). Red-stained mesothelial cells invaded the collagen layer further in all directions from the spheroids (fig. S8B). The invasive area of ACMSs was significantly larger than that of EOC cells (Fig. 3D). At 48 hours, although EOC spheroids invaded only 125% of the original spheroid area, ACMSs extended their invasion area by >400%. EOC cells from ACMSs followed the way after mesothelial cells, with the invasive area of EOC cells from ACMSs being significantly larger compared to those with only EOC spheroids (Fig. 3E). Moreover, to demonstrate that the invasion model of ACMSs was not dependent on cell lines, we used Kuramochi and OVCAR3 as representing models of HGSOC cell lines. Both cell lines did not invade the collagen layer when they formed spheroids with only EOC cells (homotypic). In contrast, when they formed ACMSs with mesothelial cells (heterotypic), mesothelial cells from ACMSs invaded the collagen layer (fig. S8, C and D). These results indicate that EOC cells can invade collagen with the assistance of mesothelial cells.

To observe the interaction between EOC spheroids and the mesothelial layer comprising the peritoneal wall, an in vitro model was established in which spheroids were placed on a single layer of blue-stained mesothelial cells. ACMSs comprising green-stained OV90 and red-stained mesothelial cells were observed every 15 min for 72 hours to reveal continuous changes in the spheroid structures and the order of invasion into the mesothelial layer (Fig. 3F). Spheroids gradually invaded the mesothelial layer and induced metastatic regions. Initially, red- and green-stained cells were randomly identified in the ACMSs (initial phase; Fig. 3G-a). When spheroids attached to the layer, red-stained mesothelial cells gradually moved from inside to outside the spheroids (attached phase; Fig. 3G-b). Then, red-stained mesothelial cells aggressively invaded the mesothelial layer (invasion phase; Fig. 3G-c). Following the appearance of mesothelial cells, green-OV90 cells started to invade the spheroids and move freely into the mesothelial layer (moving phase; Fig. 3G-d). Movie S5 illustrates these four steps. The mesothelial clearance area was significantly larger in ACMSs than in spheroids with only OV90 at each time point (Fig. 3H). Spheroids with only OV90 cells demonstrated a longer duration before invading the mesothelial layer (fig. S9A and movie S6). When spheroids comprised solely of mesothelial cells, they did not invade the mesothelial layer and remained localized in the same position (fig. S9B and movie S7). When we focused on the invasive leader cells in the mesothelial layer, almost all cells were red-stained mesothelial cells with a spindle morphology, also known as mesenchymal cells (Fig. 3I). In contrast, green-stained OV90 cells were observed inside the border area. Furthermore, blue-stained mesothelial cells identified close to the invasive front became increasingly spindle shaped, whereas they normally exhibited a cobblestone appearance in noninvasive areas (Fig. 3J). Mesothelial cells close to the invasive front exhibited expression of the epithelial mesenchymal marker vimentin in contrast to those in the noninvasive area, which showed a low level of the expression (fig. S9C). TGF-β1–stimulated mesothelial cells expressed vimentin, whereas mesothelial cells normally expressed low levels of vimentin (fig. S9D). These results indicate that mesothelial cells convert their characteristics and acquire highly aggressive features for invasion into collagen or mesothelial layers when they interact with EOC cells through spheroid formation.

Another benefit of spheroid formation is acquired resistance to anoikis and chemotherapy in ascites. Nonadherent conditions induce strong apoptosis in epithelial cells (*8*). ACMSs formed more aggregated spheroids than spheroids containing only EOC cells (Fig. 2Q and fig. S7F). As expected, the apoptosis ratio of OV90 cells was high in spheroids containing only OV90 cells (8.4%). However, when OV90 formed spheroids with mesothelial cells, the ratio was lower (3.9 or 3.7%). By forming aggregate spheroids, the EOC cells acquired anoikis resistance. These spheroids were then treated with cisplatin, and those containing only OV90 cells were the most sensitive. In contrast, OV90 cells forming ACMSs were more resistant to cisplatin treatment in both early apoptosis and dead cell fractions (fig. S9E). These results indicate that by forming ACMSs with mesothelial cells, EOC cells can acquire a high level of invasiveness and resistance for chemotherapy in ascites.

### Mesothelial cells in ACMSs invade first to induce peritoneal metastasis ex vivo and in vivo

**Fig. 4.**
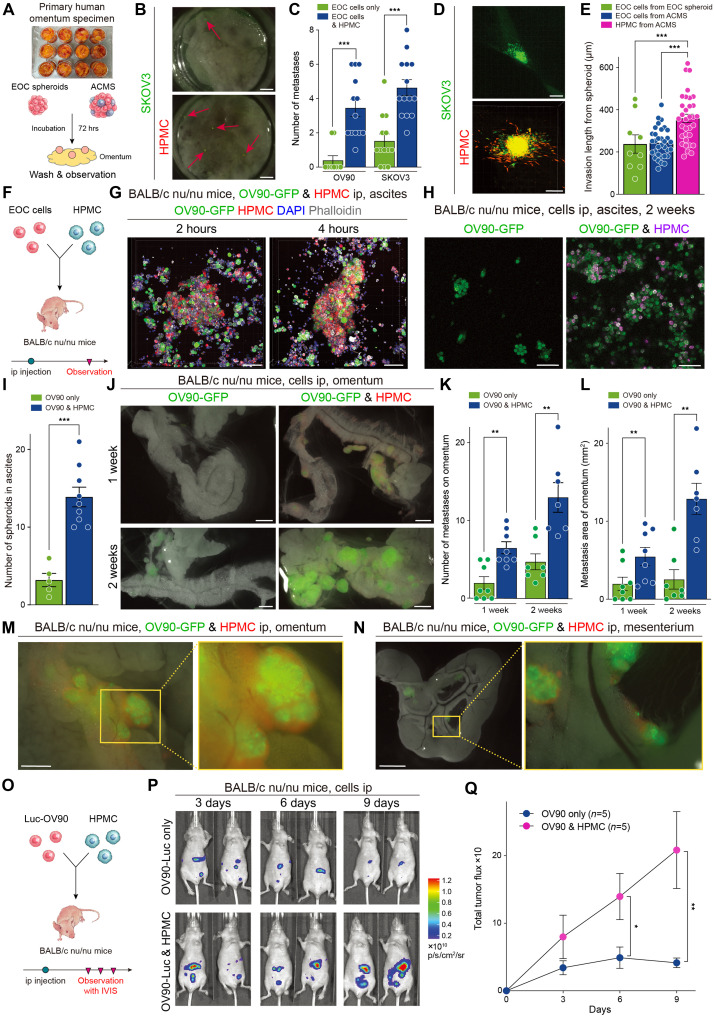
Mesothelial cells within ACMSs invaded first and induced peritoneal metastases ex vivo and in vivo. (**A**) Scheme for the ex vivo model using the resected omentum. (**B**) Images of metastases on the omentum in the ex vivo model. Red arrows denote the metastasis sites. Scale bars, 2 mm. (**C**) Bar graph showing the number of metastases in each group (OV90, *n* = 23; SKOV3, *n* = 26). (**D**) Representative confocal microscopy images of green-stained SKOV3 and red-stained mesothelial cells in the ex vivo model. Scale bars, 200 μm. (**E**) Bar graphs showing the invasion length from the original spheroid into omentum tissue. Each dot represents the number of spheroids. (**F**) Scheme of the malignant ascites in vivo model. (**G**) Spheroids composed of green-stained SKOV3 and red-stained mesothelial cells using a confocal microscopy in vivo model at 2 or 4 hours later after injection. Scale bars, 100 μm. (**H**) Differences in the formation of spheroids in ascites when mice were injected with OV90 with or without HPMCs. Scale bars, 100 μm. (**I**) Bar graph showing the number of spheroids detected in ascites. (**J**) Images of the omentum using fluorescence microscopy 1 or 2 weeks after green-stained OV90 injection. Scale bars, 1 mm. (**K**) Bar graph showing the number of omentum metastases with and without mesothelial cell injection. (**L**) Metastasis area of the omentum in different compositions of the spheroids. (**M**) Magnification images of the omentum metastasis sites. Scale bar, 750 μm. (**N**) Magnification of the mesenteric metastases. Scale bar, 2.5 mm. (**O**) Scheme for the assay of tumor burden over time in the in vivo model. (**P**) Representative bioluminescence images after luc-OV90 injection with or without mesothelial cells. (**Q**) Total tumor flux using bioluminescence images. *P < 0.05, **P < 0.01, and ***P < 0.001.

The omentum is the first abdominal site of EOC metastasis. It comprises adipocytes, fibroblasts, immune cells, and a single layer of mesothelial cells. Assessing the mechanism by which EOC spheroids induce metastases on omentum tissues requires an ex vivo experimental model of the human body. To achieve this, spheroids formed from EOC cells with or without mesothelial cells were gently placed on a small piece of the omentum (Fig. 4A). After 72 hours, ACMSs induced significantly more disseminations on the surface of the omentum than the spheroids that only contained EOC cells (Fig. 4, B and C, and fig. S10A). At the metastasis site, red-stained mesothelial cells invaded adipose tissues first, followed by green-stained EOC cells. A few EOC cells invaded when the spheroids were composed only of EOC cells (Fig. 4D and fig. S10B). The invasion length from the original spheroids was significantly greater in mesothelial cells than in EOC cells (Fig. 4E).

To determine how quickly EOC cells form spheroids in ascites, green-stained OV90 cells were injected into the abdominal cavities of mice with red-stained mesothelial cells (Fig. 4F). Ascites was collected 2 or 4 hours after injection. During the initial 2 hours, OV90 cells formed spheroids with mesothelial cells (Fig. 4G). Spheroids formed after 4 hours were more aggregated than those formed at 2 hours. These spheroids contained randomly distributed EOC and mesothelial cells. These results indicate that detached EOC cells immediately form spheroids with surrounding mesothelial cells in ascites. Thereafter, to assess the early stage of invasion, the omentum was observed 24 hours after injection using a multiphoton microscope before resection. Red-stained mesothelial cells invaded deeper into the adipose tissue surrounding the green-stained OV90 cells (fig. S10C). Tissue decolorization showed that both types of cells were present on the surface of the omentum. In contrast, only mesothelial cells were present in the deeper adipose regions (fig. S10D and movie S8). Two weeks after the peritoneal injection, we observed ascites, and the results showed numerous spheroids composed of OV90-GFP and mesothelial cells. In contrast, small number of spheroids were present in the ascites of mice injected with only OV90-GFP cells (Fig. 4, H and I). When OV90-GFP cells were injected into mice, small metastasis was observed after 1 week, and some metastases were observed after 2 weeks. In contrast, when OV90-GFP cells were injected with the same number of mesothelial cells, metastasis was induced at week 1 (Fig. 4J). The number of metastases and the metastatic area on the omentum were significantly higher and larger in mice injected with OV90-GFP and mesothelial cells than in mice injected with OV90-GFP alone (Fig. 4, K and L). Notably, when closely observing the metastatic areas on the omentum, OV90-GFP cells were identified in the center of the metastasis sites, whereas red-stained mesothelial cells surrounded the cancer cells because of invasion into the adipose tissue (Fig. 4M). The metastatic pattern in the mesenterium was the same as that for the metastases in the omentum (Fig. 4N). We also assessed peritoneal metastases following the injection of spheroids into the abdominal cavity. When the spheroids were composed of only OV90 cells, the metastatic area was negligible after week 1. In contrast, when ACMSs were injected, the metastatic area was larger, and red-stained mesothelial cells were present outside the metastatic sites (fig. S10, E and F). When only mesothelial cells were injected into the abdominal cavities of the mice, they attached to the surface of the omentum but did not invade the adipose tissue (fig. S10G).

Last, we analyzed tumor burden over time using OV90 luciferase cells (luc-OV90) and an IVIS imaging system. The burden of peritoneal metastasis was assessed on days 3, 6, and 9 after the peritoneal injection of luc-OV90 cells with or without the same number of mesothelial cells (Fig. 4O). Mice injected with mesothelial cells showed a significantly higher burden of metastasis on each day (Fig. 4, P and Q). These results support our hypothesis that mesothelial cells play an important role in the induction of metastasis through the formation of spheroids with EOC cells in ascites. Mesothelial cells invaded the collagen layer and adipose tissues before EOC cells, which then follow the same route.

### EOC cells alter the RNA expression profile of mesothelial cells through ACMS formation

**Fig. 5.**
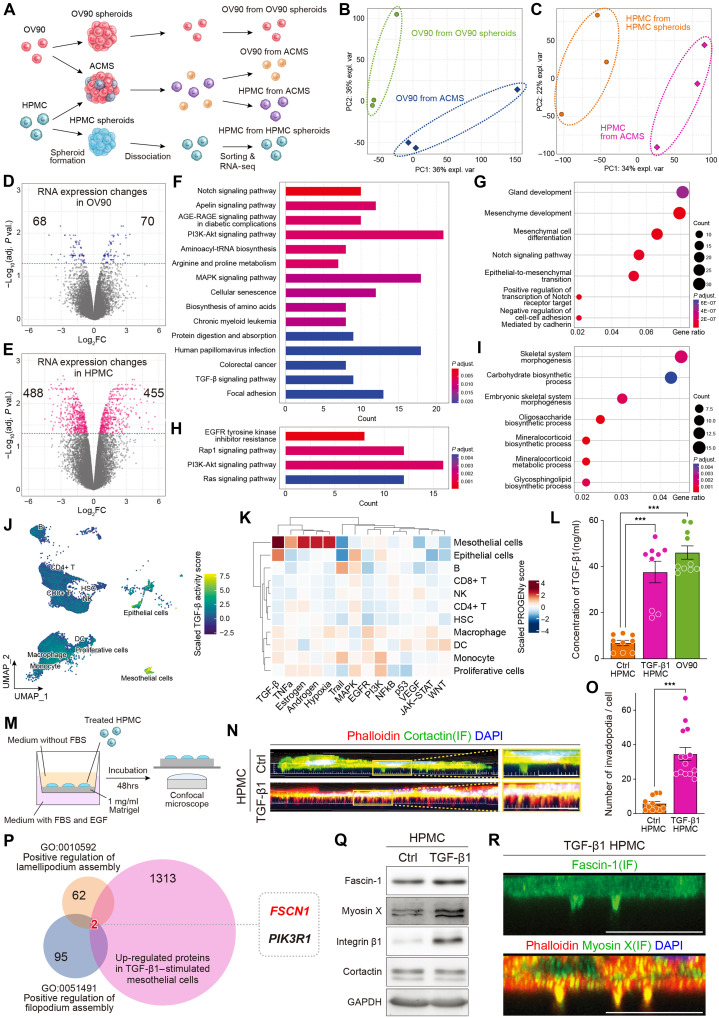
EOC cells change RNA expression in mesothelial cells via spheroid interactions. (**A**) Schematic showing the comparison between RNA expression in OV90 and mesothelial cells. (**B** and **C**) PCA plot of (B) OV90 and (C) HPMCs. (**D**) Volcano plot and clustering of RNA expression changes in OV90. The red line indicates an adjusted *P* value <0.05. (**E**) Volcano plot and clustering of RNA expression changes in HPMCs. The red line represents an adjusted *P* value <0.05. The right side of the volcano plot represents a fold change. (**F**) Significant up-regulated pathway changes in mesothelial cells after interaction with OV90 in KEGG. (**G**) Significant up-regulated pathway changes in mesothelial cells after interaction with OV90 in the GO term. (**H**) Significant down-regulated pathway changes in mesothelial cells after interaction with OV90 in KEGG. (**I**) Significant down-regulated pathway changes in mesothelial cells after interaction with OV90 in the GO term. (**J** and **K**) PROGENy pathway activity analysis of the ascites samples of the Zheng *et al.* EOC scRNA-seq dataset (*40*) revealed high TGF-β pathway activity in both EOC and mesothelial cells. (**L**) Bar plot showing the concentration of TGF-β1 in the supernatant from HPMCs, TGF-β1–stimulated HPMCs, and OV90 cells. (**M**) Scheme of an invadopodium in a mesothelial cell. (**N**) Immunofluorescence images of a single cell invading the collagen layer using invadopodium formation. Green, cortactin; red, phalloidin. Scale bars, 10 μm. (**O**) The number of invadopodia was significantly higher in TGF-β1–stimulated mesothelial cells. (**P**) Strategy to detect candidates with a high invasion ability in mesothelial cells. (**Q**) Western blot analysis of fascin-1 and several proteins related to invadopodium formation. (**R**) Immunofluorescence images of fascin-1 or myosin X (green) in TGF-β1–stimulated mesothelial cells. Scale bars, 5 μm. FACS, fluorescence-activated cell sorting; FC, fold change. ***P < 0.001.

To identify the molecular mechanisms that enable mesothelial cells to acquire aggressive invasive abilities when they form ACMSs, we analyzed RNA expression changes in both EOC and mesothelial cells. GFP-labeled OV90 and red-stained mesothelial cells formed spheroids for 72 hours and were then separated from each other using flow cytometry. Spheroids consisting of each cell type were generated, collected in the same manner, and used as controls. RNA expression levels were analyzed using RNA sequencing in (i) OV90 cells with and without the interaction with mesothelial cells and (ii) mesothelial cells with and without the interaction with OV90 cells (Fig. 5A). The principal components analysis (PCA) plot and heatmap clearly separated the two groups [Fig. 5B and fig. S11A (OV90) and Fig. 5C and fig. S11B (HPMCs)]. Gene expression changes were relatively minor in OV90 cells; only 70 genes were up-regulated (*P* < 0.05) by the interaction with mesothelial cells (Fig. 5D). In contrast, RNA expression in mesothelial cells was markedly affected by OV90 spheroid formation; the number of up-regulated genes was almost 6.5 times higher in mesothelial cells (70 versus 455) (Fig. 5E). These results show that ACMS formation markedly alters RNA expression in mesothelial cells, whereas the RNA expression profile of EOC cells is similar. Consequently, we focused primarily on RNA expression changes in mesothelial cells that underline the acquisition of invasion features.

Pathway analyses of the altered RNA expression in mesothelial cells using Kyoto Encyclopedia of Genes and Genomes (KEGG) after the interaction with EOC cells showed that the Notch, PI3K (phosphatidylinositol 3-kinase)-Akt, MAPK (mitogen-activated protein kinase), and TGF-β pathways were significantly up-regulated (Fig. 5F and fig. S11C). Gene Ontology (GO) term analysis revealed up-regulation of pathways associated with epithelial-to-mesenchymal transition (Fig. 5G). Pathway analyses of down-regulated genes, using KEGG and GO terms, identified several metabolism pathways (Fig. 5, H and I). To assess which cellular compartments were associated with these pathways, we analyzed the scRNA-seq dataset, focusing only on the ascites samples (*40*). The pathway activity analysis showed high TGF-β pathway activity in both cancer and mesothelial cells (Fig. 5, J and K). On the basis of these results, we hypothesized that the activation of the TGF-β pathway enhanced the aggressive phenotype of mesothelial cells because TGF-β1 stimulation induced morphological changes and altered protein expression, consistent with a more mesenchymal subtype (*50*, *51*). As a source of TGF-β1, we calculated the concentration of TGF-β1 in the supernatant collected from mesothelial cells, TGF-β1–stimulated mesothelial cells, and OV90. OV90 exhibited a significantly higher secretion of TGF-β1 than mesothelial cells, and TGF-β1–stimulated mesothelial cells also demonstrated increased secretion (Fig. 5L). Relative quantitative polymerase chain reaction (qPCR) expression of TGF-β1 in TGF-β1–stimulated mesothelial cells was significantly higher than that in nonstimulated mesothelial cells (fig. S11D). We cultured TGF-β1–stimulated mesothelial cells on Matrigel to observe their invasiveness (Fig. 5M). TGF-β1–stimulated mesothelial cells increased the number of invadopodia, which are an essential structure for the degradation of the ECM (Fig. 5N). The number of invadopodia was significantly higher in TGF-β1–stimulated mesothelial cells than in nonstimulated cells (5 versus more than 30 per cell) (Fig. 5O). Among the proteins up-regulated in TGF-β1–stimulated mesothelial cells, we focused on *FSCN1* and *PIK3R1* because they are related to filopodium and lamellipodium formation (GO: 0051491 and GO: 0010592), which share proteins involved in the formation of invadopodia (Fig. 5P). Moreover, spheroid formation and the ascites environment can cause hypoxic conditions in these cells (*4*, *52*). The expression of hypoxia-inducible factor 1α (HIF1A), one of the most reliable hypoxia markers, was significantly increased when mesothelial cells were stimulated with TGF-β1 and formed spheroids (fig. S11, E and F). The hypoxia-detecting probe was strongly positive in large ACMSs (fig. S11G). TGF-β1 and HIF1A can induce invadopodium precursor formation in malignant cells (*51*). Moreover, hypoxia facilitates the epithelial-to-mesenchymal transition (*33*, *53*). Therefore, we identified *FSCN1*, which was associated with aggressive invasion and invadopodium formation in mesothelial cells. Fascin-1, an actin-binding and bundling protein, plays essential roles in invadopodium formation in association with several proteins, including Tks5, integrin β1, cortactin, and myosin X (*32*, *54*). Western blotting analysis showed that the expression of fascin-1, Tks-5, integrin β1, and myosin X was up-regulated in TGF-β1–stimulated mesothelial, whereas, unexpectedly, the expression of cortactin was not altered (Fig. 5Q and fig. S11H). Tks5 was reported to have essential roles in mesothelial cells in gastric cancer (*35*). Myosin X carries integrin in invadopodia (*55*). Furthermore, fascin-1 and myosin X were localized at the invadopodia in TGF-β1–stimulated mesothelial cells (Fig. 5R). RNA sequencing and proteomic analysis of mesothelial cells showed that the TGF-β1/fascin-1 axis can confer a high invasive ability through spheroid formation with EOC cells.

### TGF-β1 stimulation from EOC cells transforms mesothelial cells into an invasive leader phenotype

**Fig. 6.**
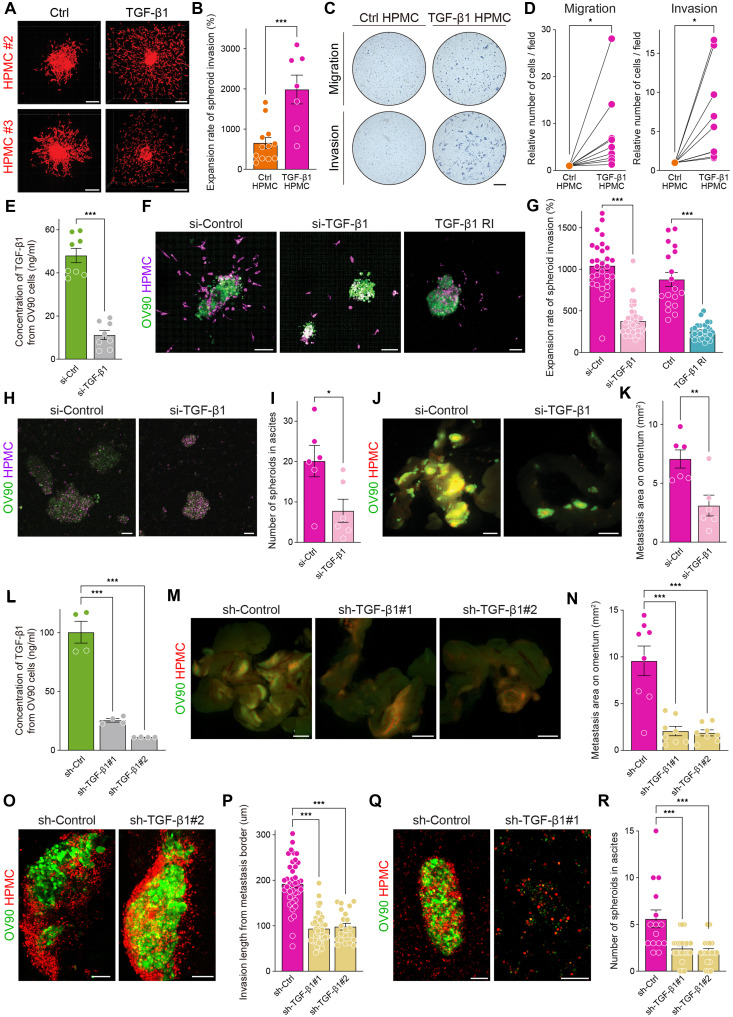
TGF-β1 stimulation from EOC cells transforms mesothelial cells into an invasive leader phenotype. (**A**) Representative images of collagen invasion of spheroids composed of HPMCs or TGF-β1–stimulated HPMCs. Scale bars, 200 μm. (**B**) The bar graph showing TGF-β1–stimulated HPMCs showed a higher invasion ability into the collagen layer. (**C**) Images of migration or invasion cells using the Transwell assay. Scale bar, 200 μm. (**D**) Linear plots showing that TGF-β1–stimulated HPMCs had a higher migration and invasion ability compared with the control HPMCs. (**E**) Bar graph showing the concentration of TGF-β1 in the supernatant in EOC cells when inhibiting TGF-β1 with siRNA. (**F**) Representative images of spheroid collagen invasion. Scale bars, 100 μm. (**G**) Bar graphs showing that inhibition of TGF-β1 by siRNA in OV90 cells or the TGF-β1 receptor blocker in HPMCs reduced the invasion ability of ACMSs compared with the control. (**H** and **I**) Differences in the spheroids in ascites when mice were injected with OV90 (green) and HPMCs (red). The number of spheroids was significantly decreased when OV90 cells were treated with siRNA for TGF-β1 than those with si-control. Scale bars, 100 μm. (**J** and **K**) Representative images of metastases on the omentum and bar graph showing the metastasis area on the omentum. Scale bars, 1000 μm. (**L**) Bar graph showing the TGF-β1 concentration in the culture supernatant in sh-control– or TGF-β1–transduced OV90 cells. (**M** and **N**) Representative images and bar graph showing the omental metastatic area (*n* = 8). Scale bars, 1000 μm. (**O** and **P**) Representative images of confocal imaging of the omental micrometastasis area and bar graph showing the invasion depth of mesothelial cells from the metastatic border. Scale bars, 100 μm. (**Q** and **R**) Representative images and bar graph showing that both the number and size of spheroids in ascites were significantly decreased in mice injected with sh-TGF-β1 no. 1 or 2 O90 cells compared with sh-control mice. Scale bars, 100 μm. *P < 0.05, **P < 0.01, and *** *P* < 0.001.

We found that spheroids with EOC and mesothelial cells were more efficient at metastasizing to other organs than spheroids with EOC cells alone (Figs. 3 and 4). To ensure the RNA sequencing data that TGF-β1 stimulation from EOC cells transforms mesothelial cells into an invasive leader phenotype, first, we have assessed the differences of invasion ability between mesothelial and TGF-β1–stimulated mesothelial cells. TGF-β1–stimulated mesothelial cells degraded more collagen than control cells using fluorescein isothiocyanate (FITC)–conjugated gelatin (fig. S12A). Spheroids formed with TGF-β1–stimulated mesothelial cells exhibited a significantly increased rate and extent of invasion into the collagen layer compared to those formed with control mesothelial cells (Fig. 6, A and B). In the Transwell assay, TGF-β1–stimulated mesothelial cells had a higher ability of both migration and invasion compared to control cells (Fig. 6, C and D). Thereafter, we assessed the effect of the invasive ability of ACMSs when TGF-β1 stimulation was inhibited, either by transfecting EOC cells with si-TGF-β1 or by treating mesothelial cells with a TGF-β1 receptor blocker before ACMS formation. We designed three small interfering RNAs (siRNAs) targeting TGF-β1 (nos. 1 to 3) and also tested a commercially available siRNA mix (no. 4); the latter showed the strongest inhibitory effect and was selected for subsequent experiments (fig. S12B). The relative qPCR expression of TGF-β1 and the concentration of TGF-β1 in the supernatant of siRNA-treated OV90 cells showed a significant decrease compared to si-control cells at both days 2 and 5 after siRNA treatment (Fig. 6E and fig. S12C) without affecting cell growth (fig. S12D). When ACMSs formed with si-TGF-β1–induced OV90 and mesothelial cells, the invasion ability into the collagen layer was significantly inhibited compared to spheroids formed with si-control–induced OV90 cells (Fig. 6F). Moreover, when ACMSs formed with mesothelial cells pretreated with a TGF-β1 receptor blocker and OV90, the expansion rate was significantly inhibited compared to ACMSs formed with control mesothelial cells (Fig. 6, F and G). The invasive length of mesothelial cells from original spheroids was significantly shorter when OV90 cells were treated with si-TGF-β1 or when mesothelial cells were treated with a TGF-β1 receptor blocker (fig. S12E). Furthermore, in vivo, the number of spheroids in ascites from mice injected with si-TGF-β1–treated OV90 and mesothelial cells was significantly lower than in mice injected with si-control cells at 3 days postinjection (Fig. 6, H and I). The progression of metastasis was also inhibited when mice were injected with OV90 cells treated with si-TGF-β1 (Fig. 6, J and K). To strengthen our in vivo findings, we transduced OV90 cells with three different short hairpin RNAs (shRNAs) targeting TGF-β1 (nos. 1 to 3). Both TGF-β1 mRNA expression (qPCR) and protein concentration in the culture supernatant were significantly decreased in sh-TGF-β1–transduced cells compared with sh-control cells (Fig. 6L and fig. S12F). On the basis of these results, sh-TGF-β1 nos. 1 and 2 were selected for the subsequent in vivo experiments. The growth rate of sh-TGF-β1–transduced OV90 cells on day 3 did not differ significantly from that of sh-control cells (fig. S12G). In vivo, the omental metastatic area was significantly reduced in mice injected with sh-TGF-β1–transduced OV90 cells compared with sh-control cells (Fig. 6, M and N). Confocal imaging of omental micrometastases further revealed that the invasive depth of mesothelial cells from the metastatic border was markedly inhibited in mice injected with sh-TGF-β1-OV90 cells relative to controls (Fig. 6, O and P). Moreover, both the number and size of spheroids in ascites were significantly decreased in mice injected with sh-TGF-β1 no. 1 or 2 O90 cells compared with sh-control mice (Fig. 6, Q and R). Consistently, the TGF-β1 concentration in ascites was significantly lower in mice receiving sh-TGF-β1–transduced OV90 cells than in control mice (fig. S12H). Last, we determined the concentration of TGF-β1 in ascites from patients with benign, borderline/early-stage, and advanced-stage cancer. Specifically, the concentration of TGF-β1 in ascites from patients with benign tumors (fibroma or teratoma) was under calculation. More than 50% of individuals with advanced-stage ascites contained TGF-β1 (>10 ng/ml) in their ascites (fig. S12I). These results indicate that TGF-β1 stimulation from EOC cells changes the mesothelial cells to more aggressive invasive characters through spheroid formation.

### Invadopodium formation mediated by fascin-1 endows an aggressive invasive ability to mesothelial cells, and stromal expression of fascin-1 correlates with poor prognosis

**Fig. 7.**
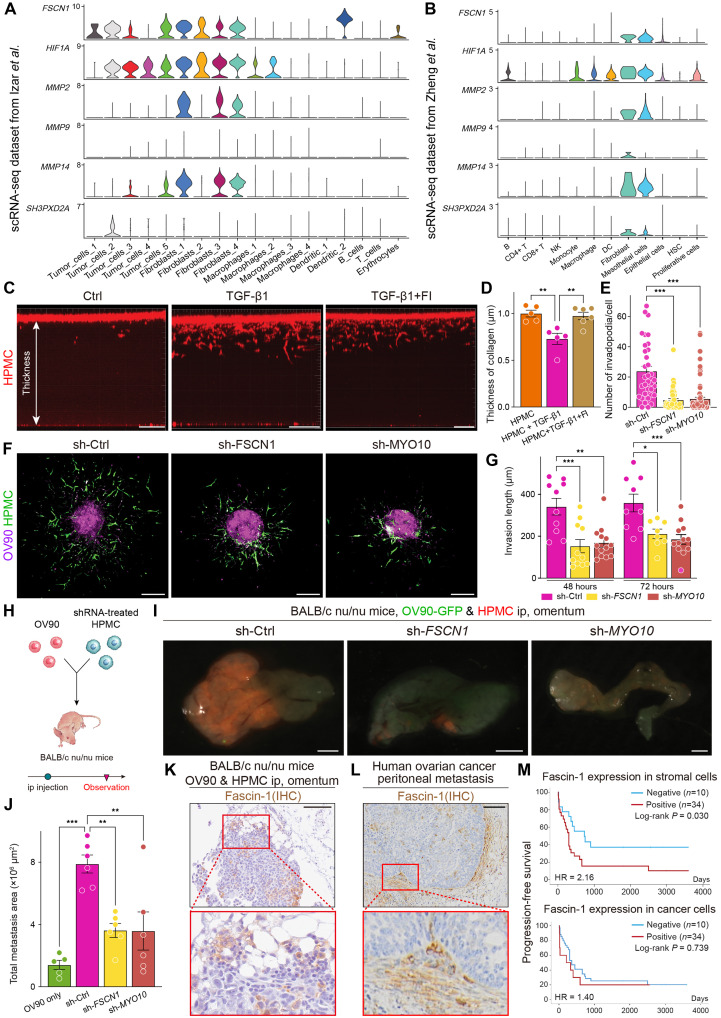
Invadopodium formation mediated by fascin-1 endows an aggressive invasive ability to mesothelial cells, and stromal expression of fascin-1 is related to a worse prognosis. (**A** and **B**) Violin plots showing the expression of invadopodium-related genes across cell components in ascites on the basis of two different scRNA-seq datasets from Izar *et al.* (*36*) and Zheng *et al.* (*40*). In the dataset of (A), mesothelial cells are classified as fibroblasts. (**C**) Collagen degradation assay. The thickness represents the cell invasion ability. Scale bars, 400 μm. (**D**) Bar graph showing the thickness of remnant collagen 48 hours after incubation. (**E**) Bar graph showing the number of invadopodia. sh-Fascin-1 or sh-myosin X inhibited invadopodium maturation. (**F** and **G**) 3D images and bar graph showing that spheroids invade collagen with shRNA-induced mesothelial cells (green) and OV90 (red). The invasion ability of mesothelial cells was significantly inhibited by sh-*FSCN1* or sh-*MYO10*. Scale bars, 200 μm. (**H**) Scheme of the malignant ascites in vivo model using shRNA-treated HPMCs. (**I** and **J**) Images and bar graph showing the differences in the metastasis area on the omentum from mice 1 week after the injection of OV90 with or without sh-induced mesothelial cells. Scale bars, 1 mm. (**K**) Representative IHC image of mouse tissue with fascin-1. Invasive stromal cells strongly expressed fascin-1. Scale bar, 100 μm. (**L**) IHC of metastasis samples in clinical samples. Fascin-1–positive stromal cells were present in the tumor-invasive regions. Scale bar, 100 μm. (**M**) Kaplan-Meier plot showing the patient’s progression-free survival depending on fascin-1 expression in stromal cells or cancer cells. Fascin-1 expression in stromal cells in metastasis samples was significantly related to a worse prognosis (*P* = 0.030). *P < 0.05, **P < 0.01, and ***P < 0.001.

We further investigated fascin-1 and myosin X because both proteins play important roles in invadopodium maturation. To degrade collagen, cells use MMP at the invadopodia, and fascin-1 is as an actin bundle protein that can carry these molecules (*53*). It is also well known that MT1-MMP (MMP14) activates MMP2 to degrade the ECM in cancer cells (*56*), and MMP14 expression facilitates spheroid formation (*57*). The MMP2 and MMP9 levels in the supernatant from TGF-β1–stimulated mesothelial cells were substantially elevated compared to those in control cells (fig. S13A). We checked the expression and location of MMP14 and found that its expression was elevated by TGF-β1 stimulation and spheroid formation in mesothelial cells (fig. S13B), and MMP14 was localized at the invadopodia (fig. S13C). We also assessed these markers using two scRNA-seq datasets shown in Fig. 1 and figs. S2A and S13D (*36*, *40*). *FSCN1* was strongly expressed in stromal cells in ascites, whereas the expression in EOC cells was not high (Fig. 7, A and B). MMP2, MMP14, and HIF1A were also strongly expressed, especially in mesothelial cells in ascites.

To further assess the importance of fascin-1 in invasion, it was inhibited using the fascin inhibitor (FI) NP-G2-044, and the collagen degradation ability was compared (fig. S13E). TGF-β1–stimulated mesothelial cells degraded collagen much faster than control mesothelial cells. The aggressive ability of cells was inhibited by treatment with the FI (Fig. 7, C and D). Next, we prepared shRNAs for the fascin-1– or myosin X–expressing vector and transfected them into mesothelial cells using the AAVpro helper-free system. Because this vector was coexpressed with the ZsGreen fluorescent protein, we could easily assess the transfection efficiency (fig. S13F). The results showed that >75% of mesothelial cells were ZsGreen-positive, and it suppressed the expression levels of fascin-1 or myosin X (fig. S13, G and H). The number of invadopodia was significantly decreased when fascin-1 or myosin X was inhibited compared with that in control cells (Fig. 7E).

ACMS was then constructed using shRNA-knockdown mesothelial and red-stained OV90 cells, and invasiveness was determined using a collagen invasion assay. When spheroids were formed with sh-*FSCN1* or sh-*MYO10* mesothelial cells, the number of invading mesothelial cells and the length of invasion from the spheroids were significantly lower than those of sh-control cells (Fig. 7, F and G). To confirm the crucial role of invadopodia in the high invasion ability of mesothelial cells, Tks5 was inhibited using siRNA in mesothelial cells. Inhibition of Tks5 in mesothelial cells decreased the invasion ability compared to the si-control (fig. S13I). Furthermore, when sh-*FSCN1* or sh-*MYO10* mesothelial cells were injected with red-stained OV90 cells into the abdominal cavities of the mice (Fig. 7H), the tumor volume and the number of metastases were significantly inhibited compared with those in sh-control cells (Fig. 7, I and J). Stromal cells at the invasive front expressed fascin-1 in metastasis tumors, whereas EOC cells did not express fascin-1 (Fig. 7K).

A previous study on EOC revealed that almost all stromal compartments showed positive staining for fascin-1 (*58*). Furthermore, higher expression levels of *FSCN1* were observed in mesothelial cells than in EOC cells using the scRNA-seq dataset from ascites samples (Fig. 7, A and B). We assessed whether *FSCN1* was associated with poor outcomes in patients with ovarian cancer using the Kaplan-Meier plotter including 1435 patients with HGSOC. These data were derived from RNA expression profiling of bulk solid tumors, including nonmalignant cells (*36*). *FSCN1* expression was associated with progression-free survival and overall survival in these patients (fig. S13J). Several studies have revealed that fascin-1 is expressed in cancer cells (*33*, *34*). Therefore, to reveal the fascin-1 expression in stromal cells and its relationship with prognosis in ovarian cancer, we evaluated 44 metastasis samples. Although fascin-1 was expressed at low levels in EOC cells, it was strongly expressed in the surrounding stromal cells (Fig. 7L). Fascin-1 expression was confirmed in 22 and 60% of EOC and stromal cells, respectively. Furthermore, fascin-1 expression in stromal cells was significantly related to worse prognosis (*P* = 0.030), whereas its expression in EOC cells was not (*P* = 0.739) (Fig. 7M). These results indicate that fascin-1 plays a vital role in inducing peritoneal metastasis by increasing the number of invadopodia with MMP14 in mesothelial cells, which degrades the surrounding collagen. Inhibition of fascin-1 could reduce the invasion ability in mesothelial cells and decrease the number of peritoneal metastases.

## DISCUSSION

**Fig. 8.**
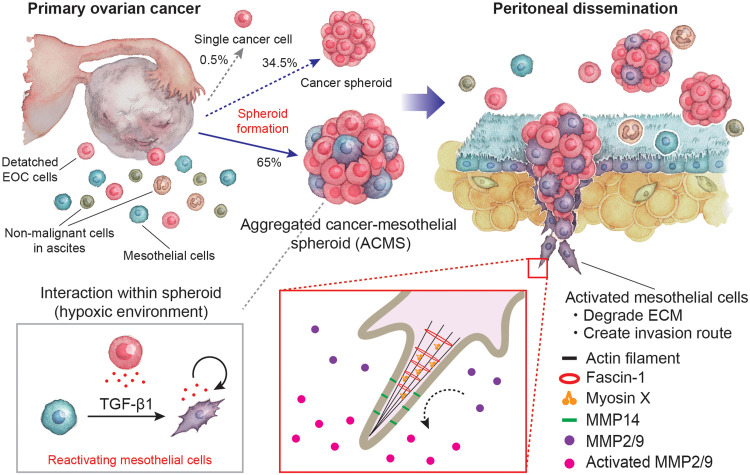
Model of mechanisms by which ovarian cancer cells survive in non-adherent ascites and rapidly form broad peritoneal metastases in the abdominal cavity. Almost all the EOC cells identified in the ascites were in a spheroids formation and 65% were accompanied by mesothelial cells, referred to as ACMSs. The formation of ACMSs enabled EOC cells to alter the RNA expression profiles of mesothelial cells via TGF-β related pathway. These alternations increased the expression of fascin-1 in this pathway, which caused invadopodia formations in mesothelial cells to mature, and this degraded collagen with MMP14. Mesothelial cells interacted with EOC cells, which aggressively invaded the collagen and mesothelial layer. These results show that EOC cells can induce peritoneal metastasis without direct dynamic RNA expression changes. EOC cells then followed the route created by the mesothelial cells. This model explains that EOC cells control the unique tumor microenvironment in ascites to rapidly induce abdominal dissemination.

In this study, the EOC cells identified in the ascites predominantly exhibited spheroid formation, with >60% being accompanied by a significant number of mesothelial cells in the ascites; these were referred to as rapidly forming ACMSs. The formation of ACMSs enabled EOC cells to alter RNA expression in mesothelial cells via TGF-β1–related pathways. These alterations increased the expression of fascin-1, resulting in maturation of invadopodium formations in mesothelial cells and subsequent collagen degradation by MMP14. Mesothelial cells engaged in aggressive interaction with EOC cells, resulting in the invasion into the collagen and mesothelial layers. These results show that EOC cells can induce peritoneal metastasis without direct dynamic RNA expression changes. EOC cells then adhere to the path established by the mesothelial cells. This study focused on how EOC cells control the unique tumor microenvironment in ascites to promptly induce abdominal dissemination immediately after detaching the primary site (Fig. 8).

The tumor microenvironment in ascites differs considerably from that of solid tumors, and thus, any analysis of scRNA-seq data should be aware of the notable changes in cellular compositions, which are very dependent on tumor locations of the HGSOC samples. However, some previous studies analyzing ascites did not take this into consideration during their analyses (*36*, *37*, *59*). Izar *et al.* (*36*) divided the cellular components in ascites as tumor cells, fibroblasts, macrophages, and immune cells, because they referred to a study that analyzed ascites using the same protocol as solid tumors (*60*). Their cell type composition of ascites differs greatly from fundamental cellular components in clinical ascites samples (*6*, *15*). Ascites must contain mesothelial cells because mesothelial cells are very specific and significant in ascites (*16*, *18*, *61*). Therefore, we reanalyzed three different datasets (*36*, *37*, *40*) and revealed that most of the “fibroblast” clusters in ascites also strongly expressed mesothelial markers. These mesothelial markers are rarely expressed in fibroblasts (*38*, *39*, *62*). This is underlined especially when we compare the stromal components of solid tumors and ascites. Although stromal cells in each region express fibroblast markers, only stromal cells in ascites strongly express mesothelial markers. A more recent scRNA-seq EOC study also supports the presence of mesothelial cells in ascites samples (*40*). Our scRNA-seq data analysis strongly demonstrates the presence of mesothelial cells in ascites, and the cellular components are similar to our pathological observations.

Most studies on ovarian cancer have focused on primary or metastatic sites despite EOC cells in ascites playing key roles in inducing peritoneal metastasis and recurrence (*5*, *6*). Images of ascites often depict EOC cells “as single cells” (*5*, *6*, *63*, *64*). Observation of clinical ascites samples showed that almost all (99%) EOC cells identified in this study were part of aggregated spheroids. The presence of EOC spheroids in ascites had a significantly worse prognosis than those with negative cytology at any stage. Although most previous studies considered that the EOC spheroid was composed only of EOC cells (*65*, *66*), we occasionally found spheroids composed of two different cell types. Most of the cells beyond the EOC cells were morphologically mesothelial cells. Distinguishing mesothelial cells from EOC cells can be challenging because of the shared expression of certain markers (*21*, *29*) and the ability of mesothelial cells to exhibit both epithelial and mesenchymal characters depending on their microenvironment (*24*, *27*, *28*, *67*). Our results revealed that using immunofluorescence staining, HBME1 could distinguish mesothelial cells from EOC cells. HBME1 is derived from a suspension of malignant mesothelioma cells and is a well-known marker used to differentiate malignant mesothelioma from carcinoma (*24*). We found that >60% of the EOC spheroids contained HBME1-positive mesothelial cells. Moreover, we found mesothelial cells in EOC spheroids using a Wt1-CreERT2 mouse model; such models are helpful for tracking specific cells (*48*). These results strongly suggest that EOC cells form aggregated spheroids with mesothelial cells in ascites.

We showed that EOC cells rapidly formed aggregated spheroids with surrounding mesothelial cells compared to spheroids with only EOC cells. Cells bind to each other via extracellular domains, and mesenchymal cells rapidly form aggregated spheroids (*13*, *68*). The expression of these molecules in EOC cells is low owing to their epithelial characteristics (*66*, *69*). In contrast, mesothelial cells express high levels of mesenchymal characters (*26*, *67*, *70*). Only a few studies have investigated the differences in spheroid formation in EOC cells with nonmalignant cells; however, existing studies have shown that cancer cells can form aggregated spheroids with nonmalignant cells, fibroblasts (*13*), and macrophages (*10*). Notably, the percentage of HBME1-positive mesothelial cells in spheroids was significantly increased in spheroids derived from patients who underwent chemotherapy. Heterocellular spheroids with EOC and mesothelial cells can be resistant to chemotherapy because they can form more aggregated spheroids (*1*, *10*, *12*, *71*). The result is in accordance with that of a previous study, which showed that a higher ratio of macrophages in spheroids was related to a worse prognosis in patients with EOC (*10*). These results indicate that EOC cells form spheroids with surrounding mesothelial cells immediately after detachment from the primary site, retaining their epithelial characteristics.

ACMS was highly invasive in the collagen layer, a monolayer of mesothelial cells, omentum tissues, and the in vivo model. Yoshihara *et al.* (*31*) showed that TGF-β1–stimulated mesothelial cells invaded first, followed by EOC cells when incubated on the collagen layer. In addition, Gaggioli *et al.* (*69*) showed that tumor-associated fibroblasts served as “leader cells” that the squamous cell carcinoma cells follow. Spheroids have a higher invasive capacity for collagen than single cells (*11*, *72*), and nonmalignant fibroblasts and mesothelial cells supported the cancer metastases (*13*, *73*, *74*). However, the mechanisms underlying this support have not yet been elucidated. To elucidate the roles of mesothelial cells in ACMSs as leader cells, we observed the induction of metastases using time-lapse imaging (movie S5). The video shows that in the first stage, mesothelial cells migrate toward the periphery of the spheroids and then invade the mesothelial layer. EOC cells can only follow after the mesothelial cells have initiated invasion. This study elucidates how nonmalignant cells in spheroids can lead to the development of peritoneal metastasis. Consequently, metastatic sites were surrounded by mesothelial cells in the in vitro, ex vivo, and in vivo models. We also revealed that mesothelial cells were present outside metastatic areas in clinical samples. On the basis of these results, we conclude that mesothelial cells in cancer spheroids invaded the collagen layer first, followed by cancer cells.

This study also investigated why mesothelial cells became aggressive after spheroid formation with EOC cells. Notably, RNA expression in mesothelial cells was markedly altered, whereas the changes in RNA expression were relatively small in EOC cells. These results may support previous studies that indicate that cancer cells can reprogram the normal omental mesothelium into cancer-associated mesothelial cells (*75*) and that cancer evolution related to genetic changes in EOC cells between primary tumors and metastases was relatively small compared with that in other types of solid tumors (*44*, *45*, *76*). For example, metastatic breast and pancreatic cancer cells exhibit considerably altered gene expression profiles compared to those at the primary site (*77*, *78*). The core mutations in EOC are involved in cell adhesion, suggesting that the interaction with other cells may be an early event in EOC progression (*44*). However, the effects of DNA stability in spheroids have not yet been determined (*5*). Nevertheless, our data show that EOC cells can acquire the ability to invade by altering the RNA expression in mesothelial cells. Considering the rapid progression of ovarian cancer, this is a reasonable explanation for its rapid metastasis. EOC cells can control the surrounding mesothelial cells and use them as leaders and may not need to alter their gene expression levels to induce peritoneal metastases.

Among the changes in RNA expression in mesothelial cells, TGF-β pathways were significantly up-regulated when mesothelial cells formed spheroids with EOC cells, and HIF1A expression was up-regulated via spheroid formation in ascites. Using the PROGENy pathway activity analysis of the ascites samples of an scRNA-seq dataset, we also corroborated that mesothelial cells compared to other cell types showed an increased activity in both of these pathways. A recent study suggests that close proximity between cells is important for the activation of TGF-β (*79*). The number of invadopodia was significantly increased in TGF-β1–stimulated mesothelial cells. Fascin-1 and myosin X, which play important roles in mature invadopodium formation, were up-regulated in TGF-β1–stimulated mesothelial cells. Inhibition of fascin-1 or myosin X decreased the number of invadopodia. Previous studies have reported increased fascin-1 expression in various types of cancer and that it is related to invasion and prognosis (*33*, *34*); however, very few studies have focused on nonmalignant cells (*58*). We found that mesothelial cells up-regulated the expression of fascin-1 and myosin X to increase invadopodium structures for aggressive invasion via spheroid formation in EOC cells. There are the same results of the previous study that fascin-1 was highly expressed in almost cases in the stromal components in ovarian cancer (*58*). Inhibiting the function of fascin-1 in mesothelial cells or TGF-β1 interaction within ACMSs reduced the invasion ability. We also revealed that the presence of fascin-1–expressing stromal cells in metastatic tissues was associated with worse prognosis. The FI is currently under a phase 2 clinical trial with a particular focus on ovarian cancer (*80*). Blocking fascin-1 or ACMS formation may be a rational treatment strategy for suppressing EOC invasion.

This study has some limitations. First, we assessed peritoneal metastasis on the basis of trans-coelomic dissemination, although several studies have mentioned a hematogenous route to peritoneal dissemination. Second, we focused only on HGSOC in ascites. Although HGSOC is the predominant histological type, other EOC types should also be evaluated. Third, we did not consider other types of nonmalignant cells in the ascites besides mesothelial cells. A strength of this study is that our hypothesis was based on clinical ascites samples. Consequently, the results of this study may reflect the biology of EOC in ascites.

In summary, we attempted to elucidate the mechanisms by which ovarian cancer cells survive in nonadherent ascites and rapidly form broad peritoneal metastases in the abdominal cavity. We found that EOC cells alter mesothelial cell characteristics through spheroid formation to acquire invasion capabilities into the mesothelial layer. Therefore, EOC cells can rapidly induce metastasis in the abdominal cavity immediately after detachment from the primary site without any dynamic RNA expression changes. These findings represent a unique characteristic of ovarian cancer in that it rapidly induces numerous peritoneal metastases within a few months of acquiring malignant characteristics, and it is difficult to detect early-stage EOC. The results also indicate that EOC cells can control the tumor microenvironment in ascites fluid. This indicates that targeting ACMSs may be a suitable treatment strategy to reduce EOC cell survival in ascites, invasion of the peritoneal cavity, and resistance to chemotherapy. Blocking fascin-1 or myosin X inhibited the aggressive invasion of mesothelial cells. Therefore, we must consider both cancer and nonmalignant mesothelial cells because they act as invasive leader cells for EOC cells.

## MATERIALS AND METHODS

### Institutional review board statement

This study was conducted with approval of the research protocol by an institutional review board: 2017-0497 (approved date: 13 March 2018) and 2020-0570-2 (approved date: 4 March 2023) by the Ethics Committee of Nagoya University and R448 (approved date: 9 August 2024) by the Ethics Committee of TOYOTA Memorial Hospital. The animal experimental protocols were approved by Nagoya University (approved number. M220294-001 and M240032-001) and by Gifu University (2021-206 and 2022-035). This study was conducted in accordance with the Declaration of Helsinki.

### Experimental design describing the patient cohort for prognosis

We retrospectively collected data from 21 facilities within the Tokai Ovarian Cancer Group from 1990 to 2022. The dataset included a total of 1856 patients with EOC. Patient backgrounds are summarized in table S1. From these patients, we selected data on those with high-grade serous carcinoma and endometrioid carcinoma (*n* = 983). We categorized these patients on the basis of the presence (positive) or absence (negative) of malignant cells in cytology slides. We then compared the patient’s backgrounds and prognoses of patients with stage I or >I disease.

### Cytology slides of clinical ascites

We retrospectively examined cytology slides of malignant ascites obtained at the time of surgery from 31 patients diagnosed with HGSOC at TOYOTA Memorial Hospital between April 2014 and September 2020. We collected written informed consent from all patients with the authorization of the Ethics Committee of TOYOTA Memorial Hospital (R448). Papanicolaou staining was performed using standard clinical methods, which proved to be highly effective for visualizing cell nuclei and cytology. We counted the number of EOC cells, both as single cells and as aggregates in spheroid formations, in three randomly selected high-power field views. In addition, four samples were prepared as cell blocks and stained with various markers. Usually, 10 to 50 ml of ascites is used to create and analyze a cytological slide. In contrast, a large volume of ascites is required to create a cell block sample. To calculate the number of nonmalignant cells in malignant ascites, we evaluated eight samples. In each case, we randomly selected four high-power field views and counted the nonmalignant cells. The classification of each cell type was based on the cytologic features as described in the pathology textbook (*61*). The distinguishing features were as follows:

1) Ovarian cancer cells: Relatively large size, high chromatin concentration, large and irregularly shaped nuclei, and very high nucleus-to-cytoplasm ratio. Nuclei often contain fragmented chromatin.

2) Lymphocytes: Small, round cells with a high nuclear-to-cytoplasmic ratio and smooth nuclear borders.

3) Neutrophils: Small cells with a triplet-shaped nucleus and a low nuclei-to-cytoplasm ratio.

4) Macrophages: Slightly larger cells with eccentric nuclei. The cytoplasm sometimes shows a gradient of hematoxylin concentration.

5) Mesothelial cells: Relatively large cells with high hematoxylin staining with centrally located, round nuclei and occasionally high chromatin concentration. The cell borders are often indistinct because of the presence of surface villi.

6) Fibroblasts: Spindle-shaped cells with a generally low nuclear-to-cytoplasmic ratio.

### Cell cultures and collection of primary mesothelial cells

The OV90 (RRID: CVCL_3768), SKOV3 (CVCL_0532), OVCAR3 (CVCL_0465), ES2 (CVCL_3509), and BJ (CRL_2522) cells were obtained from American Type Culture Collection (Manassas, VA). The A2780 (CVCL_0134) cells were purchased from the European Collection of Authenticated Cell Cultures (Salisbury, UK). Kuramochi (CVCL_1345) cells were purchased from the Japanese Collection of Research Bioresources (Tokyo, Japan). The ID8 mouse ovarian surface epithelial cell line (no. SCC145) was purchased from Merck KGaA (Darmstadt, Germany). LP9 (AG07086) cells were purchased from Coriell Institute (Camden, NJ). These cells were maintained with RPMI 1640 medium (Nacalai Tesque, Kyoto, Japan) supplemented with 10% fetal bovine serum (FBS) and penicillin/streptomycin. All experiments were performed after verification that the cells were mycoplasma-free. To generate OV90 or ID8 stably expressing GFP or luciferase (luc), a recombinant retrovirus was used.

HPMCs were collected according to a previous report (*81*). Briefly, the tumor-free omentum was used to collect mesothelial cells from patients with borderline ovarian carcinoma. The resected omentum was cut into 1-cm^2^ pieces and trypsinized with 0.25% trypsin at 37°C for 30 min. The suspension was passed through a 100-μm filter to remove undigested fragments. The filtered medium was centrifuged at 300*g* for 5 min. After removing the supernatant, the collected cells were cultured in RPMI 1640 with 10% FBS on a collagen-coated dish (no. 4020-010, IWAKI, Tokyo, Japan). The following day, the medium was changed, and incubation was continued for 3 days. At confluence, the cells exhibited a cobblestone appearance. If many spindle-shaped cells persisted even at confluence, these cells were not used as mesothelial cells. Mesothelial cells were used within three passages. In this study, we used mesothelial cells derived from six distinct sources (HPMC nos. 1 to 6). TGF-β1–stimulated mesothelial cells were generated by stimulation with TGF-β1 (10 ng/ml; AF-100-21C, Peprotech, NJ) for 72 hours.

### Immunofluorescence for EOC cell lines, primary ascites, and mesothelial cells

Glass coverslips (15 mm in diameter, no. 1, Matsunami) were coated with collagen (0.5 mg/ml; KP-8000, Nitta Gelatin, Osaka, Japan). Cells were cultured on these coverslips. After fixation with 4% paraformaldehyde (PFA), mesothelial cells were stained with the following antibodies: calretinin (ab133316, rabbit, 1/200), EpiCAM (ab213500, rabbit, 1/100), αSMA (ab124964, rabbit, 1/400), pancytokeratin-allophycocyanin (APC) (REA831, 1/50), WT1 (sc-7385, mouse, 1/50), HBME1 (Dako, mouse, 1/50), podoplanin (BioLegend, 916606, mouse, 1/200), CK7 (ab181598, rabbit, 1/200), PAX8 (Proteintech, 10336-1-AP, rabbit, 1/200), p53 (DO-1: sc-126, mouse, 1/50), LRRN4 (HPA075974, Sigma-Aldrich, rabbit, 1/150), vimentin (D21H3: Cell Signaling, no. 5741, rabbit, 1/50), N-cadherin (D4R1N, Cell Signaling, no. 13116, rabbit, 1/50), PDGFRα (no. 3146, Cell Signaling, rabbit, 1/50), desmin (no. 5322, CST, rabbit, 1/100), MSLN (SC-33672, Santa Cruz, mouse, 1/50), UPK3B (NBP1-92564, NOVUS, rabbit, 1/500), KRT8 (M0631, DAKO, 1/50), and KRT18 (NBP2-44951, NOVUS, mouse, 1/100). To stain the nucleus and cell structure, 4′,6-diamidino-2-phenylindole (DAPI; Roche, 236276, 1/1000) and phalloidin-iFluor 647 (ab176759, 1/500) or Alexa Fluor 594 phalloidin (A12381, 1/500) were applied according to the manufacturer’s instructions. Staining was observed using a Nikon AR X laser confocal microscopy system with a Plan Apo λD 20× OFN25 DIC N2 objective lens (Nikon, Tokyo Japan).

### Whole-exome sequencing in primary mesothelial cells

Primary mesothelial cells from five different sources were cultured in a collagen-coated six-well plate. Upon reaching confluence, we collected these cells’ DNA using the NucleoSpin DNA RapidLyse kit (Takara Bio, Kusatsu, Japan). DNA concentrations were confirmed to be greater than 20 ng/μl for all samples. Whole-exome sequencing was performed using the Illumina NovaSeq X plus platform at Rhelixa. The total analyzed exonic region covered ~60,250,365 base pairs in this study. The quality of the raw paired-end sequence reads was assessed with FastQC (version 0.11.7). Low-quality bases (Phred score <20) and adapter sequences were trimmed using Trimmomatic software (version 0.38). The cleaned reads were aligned to the reference genome using bwa-mem (version 0.7.17-r1188). Duplicate reads were removed, and alignment was refined around known indels using tools from the GATK (Genome Analysis Toolkit). Base quality score recalibration was performed with GATK ApplyBQSR (version 4.0.8.1). Copy number variations (CNVs) were analyzed using CNVkit (version 0.9.8). Genetic mutation data from EOC-specific genes in EOC cell lines (Kuramochi, OVCAR3, OV90, and SKOV3) were referenced from Barnes *et al.* (*46*).

### Cell block creation and IHC

Cell blocks were created from ascites samples. The collected ascites samples were centrifuged at 1500 rpm for 15 min. After removing the supernatant, 4% PFA was added to fix the cellular components for 15 min. The sample was then centrifuged at 1500 rpm for 5 min, and the supernatant was gently removed. A high concentration of array-gel (USM-J18, YoukenScience, Tokyo) was added, and the sample was centrifuged again at 1500 rpm for 5 min. A coagulant was added to fix the sample, which was then transferred to a cassette to prepare formalin-fixed paraffin-embedded (FFPE) blocks for use as cell block samples. From the FFPE blocks, 4-μm-thick sections were prepared and stained for hematoxylin and eosin, CK7 (Abcam, ab181598, rabbit, 1/200), HBME1 (Dako, M3505, mouse, 1/50), PAX8 (Proteintech, 10336-1-AP, rabbit, 1/200), or podoplanin (BioLegend, 916606, mouse, 1/200). A blocking buffer without a primary antibody was used as a negative control. For antigen retrieval, sections were heated at 120°C for 1 min in 10 mM citrate buffer (pH 6.0) with Pascal (DAKO).

Ten primary samples from the ovary were included for IHC analysis of HBME1 (1/50), and 44 metastasis samples from the omentum of the patients with HGSOC were included for the IHC analysis of fascin-1 (sc-21743, mouse, 1/200) and podoplanin (BioLegend, 916606, mouse, 1/200). These patients had not received chemotherapy before surgery. Patient’s clinical information was retrospectively collected. Consecutive sections were prepared from FFPE blocks and stained for these markers.

### Isolation of spheroids from clinical ascites samples

We prospectively collected the ascites samples at Nagoya University Hospital from January 2021 to December 2023, in accordance with the guidelines of the Ethics Committee of Nagoya University (2020-0570-2). We collected written informed consent from all patients. The first 14 cases were used to evaluate whether mesothelial cells were present in EOC spheroids. To examine differences in the proportion of HBME1-positive cells in spheroids, an additional 10 samples collected before and after chemotherapy were included.

To separate cellular components from erythrocytes, we used the Percoll (GE Healthcare, Chicago, IL) density gradient centrifugation method (*47*). Ascites fluid was centrifuged at 300*g* for 5 min. The pellet was suspended in serum-free RPMI 1640 medium, gently overlaid on Percoll [50% with phosphate-buffered saline (PBS)], and then centrifuged at 1200*g* for 20 min without braking. The interphase layer was carefully collected, diluted with RPMI 1640 medium, and centrifuged at 300*g* for 5 min. The pelleted cellular component was fixed with 4% PFA and stored at 4°C until further analysis.

### scRNA-seq analysis using public datasets

All scRNA-seq data used in this study are publicly available. The human ovarian cancer Chromium 10x Genomics scRNA-seq datasets were obtained from Mendeley Data (*40*), Synapse (accession number syn25569736) (*37*), and the Gene Expression Omnibus (GEO) repository with the accession number GSE146026 (*36*). The human parietal pleura Chromium 10x Genomics scRNA-seq dataset was downloaded from the GEO repository with the accession number GSE243446 (*41*).

Processed expression matrices, UMAP/tSNE (t-distributed stochastic neighbor embedding) coordinates, cell type annotations, and clinical metadata, e.g., tumor locations, for all datasets were used as indicated in the original publication sources. The datasets were handled using the Seurat package (version 5.1.0) (*41*) in R (version 4.4.1). Clusters, results from gene expression level analyses, and proportions of cell types across tumor locations were visualized using the scCustomize (version 2.1.2) (https://zenodo.org/records/14529706) and SCpubr (version 2.0.2) (*82*) packages.

The gene expression signature scores of fibroblasts and mesothelial cells were calculated using the AddModuleScore function in Seurat using default parameters. In addition to the prototypical marker genes of fibroblasts (COL1A1, COL6A1, DCN, FAP, FBLN1, and LUM) and mesothelial cells (CALB2, LRRN4, MSLN, SLPI, UPK3B, and WT1), the top 30 differently expressed genes between the fibroblast and mesothelial cell populations in the pleura (*41*) and Zheng *et al.* HGSOC datasets (*40*), respectively, were used as signatures of each cell population. The Wilcoxon test as implemented in the SCpubr package was used to statistically compare the different groups of samples.

We used the PROGENy package (version 1.26.0) (*83*) for the pathway activity analysis of the Zheng *et al.* ovarian cancer ascites samples (*40*). Cell type populations containing more than 10 cells were analyzed using the 500 most responsive genes per pathway as recommended by the PROGENy developers. The PROGENy pathway activity scores were summarized by cell type and shown as a heatmap using the ComplexHeatmap (version 2.20.0) package (*84*).

### Observation of whole spheroids with immunofluorescence using a multiphoton microscope

Cellular components from ascites fluid in 4% PFA were pelleted and resuspended with 0.1% Triton X-100 in PBS for 5 min. The permeabilized cells were washed with PBS and treated with 1% bovine serum albumin in PBS for more than 1 hour. After washing out the bovine serum albumin, we added the primary antibodies (HBME1, calretinin, WT1, CK7, or PAX8) and incubated them overnight at 4°C with gentle agitation. The Alexa Fluor 488–conjugated secondary antibody (Life Technologies) and phalloidin-iFluor 647 (Abcam) were added to the PBS-washed cells and incubated for several hours at room temperature with gentle agitation. The cells were washed several times with PBS and observed using the Nikon A1RMP multiphoton confocal microscopy system with an Apo LWD 25× DIC N2 objective lens (Nikon). Imaris software (Oxford Instruments) was used to generate 3D images from the microscope data obtained by varying the *z* axis. We stained HBME1 in spheroids from 28 patients and assessed 383 spheroids to determine whether HBME1-positive cells were present in the spheroids. We also compared the proportion of HBME1-positive cells in spheroids before and after chemotherapy (15 versus 13 patients).

### Cre-ER model and ascites specimen

To trace mesothelial cells in the peritoneal cavity of mice, we generated Wt1-CreERT2; Rosa-LSL-tdTomato mice. Wt1-CreERT2 [Wt1tm2(cre/ERT2)Wtp/J, Jax no. 010912] and Rosa-LSL-tdTomato [B6;129S6-Gt (ROSA)26Sor tm9(CAG-tdTomato)Hze/J, Jax no. 007909] were purchased from the Jackson Laboratory. To establish the reporter system using tdTomato, the Wt1-CreERT2 mice were crossed with Rosa-LSL-tdTomato reporter mice (Wt1-CreERT2; Rosa-LSL-tdTomato mice). For tamoxifen treatment, CreERT2 activity was induced by intragastric administration of tamoxifen (T-5648, Sigma-Aldrich) in corn oil at 2 mg per mouse every other day for a total of five doses. One month after tamoxifen treatment, we evaluated the location of Tomato-positive cells in the abdominal wall.

Using the mouse model, we injected 1 × 10^6^ ID8-GFP cells into the mouse abdominal cavity. One month later, when ID8 cells induced metastases in the abdominal cavity, we collected malignant ascites from the mice. Cellular components were isolated and observed under the Nikon AX-R confocal microscopy system with a Plan Apo λ 10× objective lens (Nikon). *Z*-stack images were constructed using Imaris software from images taken by varying the *z* axis.

### Observation of spheroid formation with time-lapse imaging

To observe spheroid formation, 2000 CellTracker Red (CMTPX, Thermo Fisher Scientific, Waltham, MA)–stained EOC cancer cells were mixed with an equal number of unstained EOC cancer cells or human mesothelial cells in 200 μl of RPMI 1640 medium containing 10% FBS. The mixture was placed into one well of a 96-well ultralow-attachment round-bottom plate (no. 7007, Costar). The plates were then set in the IncuCyte (IncuCyte ZOOM) system with a 10× objective. Red fluorescence images were taken at 1-h intervals for 72 hours. As spheroids formed, the area size of red fluorescence gradually decreased. We plotted the area size at each time point using Zoom Basic Analyzer software (Essen BioScience). The fluorescence images were detected using the top-hat-hand background subtraction for each image [radius: 100 μm; threshold: 2.0 Green Calibrated Unit (GCU)]. We analyzed two different ovarian cancer cell lines (OV90 and SKOV3) with two different primary mesothelial cells (HPMC nos. 1 and 2). All media were filtered through a 0.45-μm filter (SLHVR33RS, Millipore) before use in the spheroid formation assay.

### Collagen invasion assay

On a 60-mm Nunclon Sphera dish (no. 174944, Thermo Fisher Scientific), 7 × 10^5^ CellTracker-stained EOC cells were cultured with or without the same number of differently stained mesothelial cells for 3 days. Nonspheroidal cells were removed using a 100-μm filter (no. 352360, Falcon). To prevent the direct contact of the spheroids with the glass bottom, 100 μl of collagen type I-A (2 mg/ml, Nitta Gelatin) containing 5× RPMI (Nitta Gelatin) and epidermal growth factor (EGF; 5 ng/ml) was added to a 24-well glass-bottom culture plate (no. 5826-024, IWAKI). After incubation at 37°C for 2 hours to allow the collagen gel to solidify, we applied the spheroid-containing collagen gel without EGF. Once the collagen gel solidified, 500 μl of serum-free medium was added to the gel and incubated for 3 days. The spheroids embedded in the collagen gel were observed using a Nikon AX-R confocal microscopy system with a Plan Apo λ 10× objective lens. *Z*-stacked images were generated using Imaris software. The spheroid expansion rate was calculated using manually traced *Z*-stack images using ImageJ software (version 1.53e, National Institutes of Health, Bethesda, MD).

### Human mesothelial cell layer clearance assay

A human mesothelial cell layer was formed on the surface of 15-mm collagen I–coated glass coverslips using HPMCs and stained with CellTracker (blue, CMAC, Thermo Fisher Scientific). Each coverslip was placed on the bottom of a well in a 24-well glass-bottom culture plate with the mesothelial layer facing up. To form EOC spheroids, 4 × 10^6^ CellTracker (green, CMFDA, Thermo Fisher Scientific)–stained EOC cells, with or without an equal number of CellTracker (red)–stained mesothelial cells, were incubated for 72 hours in 6 ml of RPMI 1640 media on the surface of a 60-mm cell-repellent surface dish (no. 628979, Greiner Bio-one) or an Nunclon Sphera dish (no. 174944, Thermo Fisher Scientific). To remove single cells or smaller spheroids, the incubated cells were filtered through a 40-μm strainer (no. 352340, Falcon). Spheroids larger than 40 μm in diameter were placed on the mesothelial cell layer and observed immediately using a BZ-X800 microscope (Keyence, Osaka Japan) with an image cytometer module (BZ-H4C, Keyence). After 72 hours, the plate was fixed with 4% PFA and further analyzed using a Nikon AX-R confocal microscopy system with a Plan Apo λ 10× objective lens.

### Apoptosis assay and FACS

To form spheroids, CellTracker Green–stained OV90 cells (2 × 10^3^) were cocultured with or without an equal number of mesothelial cells in 200 μl of RPMI 1640 medium with 10% FBS in a 96-well ultralow-attachment round-bottom plate for 72 hours. The spheroids were then treated with cisplatin (10 or 78 μM) (Nichi-iko, Toyama, Japan) for 48 hours. The collected spheroids were trypsinized to analyze early apoptotic and dead cells within the spheroids. The dissociated cells were stained with APC annexin V and 7-aminoactinomycin D (7-AAD) (BD Biosciences, San Jose, CA) according to the manufacturer’s instructions. Each sample was prepared under four different conditions (annexin V−/7-AAD−, annexin V+/7-AAD−, annexin V−/7-AAD+, and annexin V+/7-AAD+) to set an accurate fluorescent gate. Annexin V+ or 7-AAD+ cells were analyzed using a FACSAria II flow cytometer (BD Biosciences, Tokyo, Japan). We defined early apoptosis cells as annexin V+/7-AAD− and dead cells as annexin V+/7-AAD+ because annexin V binds to intracellular phosphatidylserine, whereas 7-ADD binds to DNA.

### Ex vivo assay

EOC and mesothelial cells were stained with CellTracker Green (8 nM) and Red (6 nM), respectively. EOC spheroids, with or without mesothelial cells, were formed in a 96-well ultralow-attachment plate for 72 hours. Omentum tissues without tumors, obtained from patients suspected of borderline ovarian cancer, were cut into small pieces and placed into each well of a 24-well plate. Spheroids were collected and gently seeded onto the tissue (eight spheroids per well) in RPMI 1640 medium. After 2 days, we examined the omentum surface using a fluorescence stereomicroscope (M205FA, Leica) and counted the number of metastases. For detailed observation of the invasive front of EOC into adipose tissue, each tissue sample was fixed with 4% PFA and further analyzed using confocal microscopy (TiE-A1R, Nikon). We measured the distance from the edge of the original spheroid to the three most invasive mesothelial and EOC cells under each condition and calculated the average invasion depth.

### In vivo spheroid formation in the abdominal cavity and early metastasis

A total of 1 × 10^6^ OV90 single cells, stained with CellTracker Green (5 nM), with or without an equal number of mesothelial cells stained with CellTracker Red (2 nM), were injected into the abdominal cavity of 6- to 7-week-old BALB/c nu/nu nude mice (Japan SLC, Nagoya, Japan) with 300 μl of serum-free RPMI 1640 medium. Two to four hours after injection, the mice were euthanized, and free-floating abdominal cells were collected with 1 ml of PBS. The collected cells were pelleted by centrifugation and fixed with 4% PFA for 5 min. Cellular components were stained with DAPI and phalloidin and observed using laser confocal microscopy (TiE-A1R). To observe early EOC metastasis on the omentum, tissues from EOC-injected mice were dissected 24 hours after injection and fixed with 4% PFA. EOC cells were then visualized using an A1RMP multiphoton confocal microscopy system. Furthermore, for a perspective view, the PFA-fixed omentum was treated with CUBIC-L and CUBIC-R+ reagents (TCI, Tokyo Japan) and observed using a Lightsheet7 microscope (Carl Zeiss, Jena, Germany).

### In vivo model of malignant ascites and carcinogenesis

Mesothelial cells were stained with CellTracker Red (6 nM) for 20 min. A total of 1 × 10^6^ single OV90 cells, constitutively expressing GFP, were suspended in 500 μl of serum-free RPMI 1640 medium with or without an equal number of mesothelial cells. This cell suspension was injected into the abdominal cavity of 6- to 7-week-old female BALB/c nude mice using a 24G needle. After 1 or 2 weeks, the mice were euthanized. A small incision was made to inject 1 ml of PBS into the abdominal cavity, followed by a 1-min wash to collect as malignant ascites. To quantify the number of spheroid structures in ascites, 50 μl of the collected ascites fluid was used to count spheroids larger than 50 μm in diameter. The remnant fluid was centrifuged at 300*g* for 5 min to collect cellular components. The abdominal cavity was then examined for metastasis using a fluorescence stereomicroscope (M205FA). Metastatic sites >0.05 mm^2^ on the peritoneal cavity were counted and analyzed.

To assess OV90 carcinogenesis with or without mesothelial cells, we injected 2 × 10^6^ OV90 cells constitutively expressing luciferase (luc-OV90) with or without the same number of mesothelial cells into the abdominal cavity of each nude mouse using 300 μl of serum-free RPMI 1640 medium. Tumor burden was evaluated on days 3, 6, and 9 using an IVIS Imaging System (IVIS Spectrum, PerkinElmer) 10 min after the intraperitoneal administration of luciferin (100 mg/kg; Thermo Fisher Scientific). Total flux (photons/s cm^−2^) was compared between two groups at each time point using Living Image software version 4.4.

### RNA sequence analysis

OV90-GFP–expressing cells and three different primary HPMCs, which were stained with CellTracker Red (5 nM), were prepared. Three types of cell components were incubated for 72 hours to form spheroids in ultralow-attachment 96-well round-bottom plates: (i) OV90-GFP only (2000 cells per well), (ii) HPMCs only (4000 cells per well), and (iii) a coculture of OV90-GFP cells and HPMCs (2000 cells per well each). Spheroids were disaggregated using trypsin for 5 min and then filtrated through a 10-μm filter before FACS (fluorescence-activated cell sorting) analysis. The OV90 and HPMCs were separated on the basis of fluorescence (OV90-GFP as green and HPMCs as red). This procedure was performed for both single and cocultures. The number of collecting cells was counted, and RNA was extracted using the ReliaPrep RNA Miniprep System (Promega) according to the manufacturer’s instructions. RNA samples were diluted in 30 μl of nuclease-free water (two extractions of 15 μl each). RNA concentrations were confirmed to be >10 ng/μl for all samples. RNA sequencing was performed using the SMART-Sweq v4 Ultra Low Input RNA Kit for Sequencing at Takara Bio. RNA sequencing data were analyzed using GensSpring (version 14.9.1, Agilent), with low-expressed genes filtered by applying a 20% minimum percentile cutoff to minimize noise. The output was further processed in R (version 4.2.3) with RStudio (version 2023.06.0+421) as an integrated development environment. Intensities were log_2_ transformed and median centered to produce the expression matrix. For clustering, PCA, differentially expressed gene analysis, GO analysis, and gene set enrichment analysis, the following R packages were used: mixOmics package (version 6.22.0) (*85*), Limma package (version 3.54.2) (*86*), and clusterProfiler package (version 4.6.2) (*87*). Gene set enrichment analysis was performed using a desktop application (www.broadinstitute.org/gsea/index.jsp). Human transcript models were based on hg38 (National Center for Biotechnology Information). RNA sequencing data were compared between two groups: (i) OV90 spheroids with and without mesothelial cells and (ii) mesothelial spheroids with and without OV90 cells. Differential expression analysis was conducted using the Limma package, and PCA was performed using the mixOmics package. Statistically significant RNA expression was defined as a *P* value <0.05 and a fold change >2.0 in heterocellular spheroids compared to single-cell spheroids.

### Invadopodium observation assay

Mesothelial cells, with or without TGF-β1 (10 ng/ml) treatment, were plated on a Cell Culture Insert (1.0-μm pore size, Falcon, 353104) pretreated with Matrigel (1 mg/ml; Corning, 354234). These inserts were incubated in RPMI medium, with only the lower chamber containing 10% FBS and EGF (5 ng/ml). After 2 days of incubation, the inserts were fixed with 4% PFA, permeabilized with 0.1% Triton X-100, and stained with various antibodies, DAPI, and phalloidin. Photographs were captured at different *z* axes using an Olympus FV1000 laser confocal microscope equipped with a UPlanSApo 60× oil objective or a Nikon TiE-A1R laser confocal microscope with a Plan Apo λ 60× oil objective. Cross-sectional images were reconstructed using Imaris software. Protrusions from the cell bottom were counted as invadopodia.

### Gelatin invadopodium assay

Mesothelial cells, with or without TGF-β1 (10 ng/ml) treatment, were plated onto FITC-gelatin–coated cover glass. After 2 days of incubation, the cells were fixed, permeabilized, and stained with DAPI and phalloidin. Images were captured using an FV1000 confocal microscope. Active invadopodia degraded the FITC-gelatin beneath the cells, producing black spots in the FITC (green) channel.

### Proteomic data analysis

We used proteomic data from our previous study (*31*), which included protein expression profiles in mesothelial cells and proteins up-regulated in mesothelial cells treated with TGF-β1 (10 ng/ml). We analyzed all proteins detected in each sample, and values falling below the detection sensitivity threshold were substituted with an adjusted minimum detection value. Proteins showing a 1.4-fold or greater increase in TGF-β1–stimulated mesothelial cells compared to control mesothelial cells were considered significant.

### GO and Kaplan-Meier analysis

GO sources related to the positive regulation of filopodium assembly (GO: 0051491) and positive regulation of lamellipodium assembly (GO: 0010592) were obtained from the public database at The Gene Ontology Resource (https://geneontology.org/). Kaplan**-**Meier analysis was conducted using KMplot software (http://kmplot.com/analysis) on the basis of public microarray datasets. The results were derived from 1435 patients with EOC of all stages. We analyzed progression-free survival and overall survival on the basis of the detected protein levels, focusing on FSCN1 (201564). *P* values (log-rank *P*) <0.05 were defined as significant prognostic factors.

### Western blot analysis

Primary mesothelial and TGF-β1–stimulated mesothelial cells were lysed in 2× SDS–polyacrylamide gel electrophoresis sample buffer [80 mM tris-HCl (pH 6.8), 2% SDS, 15% glycerol, 0.002% bromophenol blue, and 100 mM dithiothreitol]. These samples were separated by SDS–polyacrylamide gel electrophoresis and transferred onto an Immobilon-P membrane (Merck Millipore). The following primary antibodies were used: fascin-1 (Merck Millipore, MAB3582), myosin X (Novus Biologicals, 22430002), integrin β1 (BD Biosciences, 610467), cortactin (BD Biosciences, 610049), Tks5 (Santa Cruz Biotechnology, sc-30122), and HIF1A (R&D Systems, 241809). Immunoreactive signals were detected using enhanced chemiluminescence (GE Healthcare) with the ImageQuant LAS 4000 mini kit (GE Healthcare). Band intensities were quantified using ImageJ software, and all protein quantification data were normalized to the expression levels of GAPDH (glyceraldehyde-3-phosphate dehydrogenase) or β-actin.

### Hypoxia detection assay

To detect the hypoxia condition in spheroids, we used the MAR hypoxia-detecting probe (Goryo Chemical, Sapporo, Japan). After forming spheroids and washing with PBS, a 1 μM solution was added and incubated at 37°C for 1 hour. After removing the solution and washing the spheroids with PBS twice, 10% FBS/RPMI medium was added and incubated at 37°C for 3 hours. Photographs were captured at different *z* axes using a Nikon TiE-A1R laser confocal microscope with a Plan Apo λ 10× objective lens.

### Transwell assay

To assess the invasive ability of TGF-β1–stimulated mesothelial cells, we used Transwell assay kits (8 μm, Corning Japan, Tokyo, Japan) according to the manufacturer’s instructions and our previous study (*88*). A total of 1 × 10^6^ control mesothelial and TGF-β1–stimulated mesothelial cells were seeded in the upper chamber with 500 μl of nonserum RPMI medium. In the lower chamber, 500 μl of 10% FBS/RPMI medium was added. We removed the upper chamber after 22 hours of incubation, and the upper chamber was stained by May-Giemsa staining. After removing noninvasive or nonmigratory cells from the upper membrane and fixing, we counted the number of invasive or migratory cells and compared them between TGF-β1–stimulated mesothelial and control cells. We have used three different sources of HPMCs. Images were obtained using a BX43 upright microscope (Olympus, Tokyo, Japan) and counted the number of migration/invasion cells.

### Collagen degradation assay

Mesothelial cells were stimulated with TGF-β1 (10 ng/ml) in 1% FBS medium for 72 hours. Control mesothelial cells were cultured with 1% FBS medium without TGF-β1. The cells were stained with CellTracker Red (5 nM), and 1 × 10^5^ cells were plated on 100 μl of collagen gel (3 mg/ml) that had been solidified in a Cell Culture Insert (1.0-μm pore size, Falcon, 353104). These inserts were incubated in RPMI medium, with only the lower chamber containing 10% FBS and EGF. After 48 hours of incubation, the inserts were fixed with 4% PFA, and the collagen gels were excised from the Cell Culture Inserts. The excised gels were placed onto glass-base dishes (no. 3910-035, IWAKI) and observed using a Nikon AX-R confocal microscopy system equipped with a Plan Apo λ 10× objective. Cross-sectional images were reconstructed using Imaris software, and the thickness of the collagen was measured. In some experiments, a FI (NP-G2-044, T9107, TargetMol, MA) was added to evaluate the role of fascin-1 in mesothelial cell–mediated collagen degradation.

### Gelatin zymography

The same number of mesothelial and TGF-β1–treated mesothelial cells was cultured on collagen-coated dishes in RPMI 1640 culture medium without serum for 2 days. Conditioned media were collected from each dish and centrifuged at 15,000 rpm to remove large cell debris. The SDS-PAGE sample buffer without a reducing agent was added to each supernatant. Samples were loaded onto gelatin (1 mg/ml)–embedded acrylamide gel. The electrophoresed gel was soaked in washing buffer (2.5% Triton X-100, 50 mM tris-HCl, pH 7.5, 5 mM CaCl_2_, and 1 μM ZnCl_2_) twice for 30 min and then rinsed with incubation buffer (1% Triton X-100, 50 mM tris-HCl, pH 7.5, 5 mM CaCl_2_, and 1 μM ZnCl_2_) at 37°C for 10 min. The incubation buffer was replaced, and the gel was incubated at 37°C for 24 hours with gentle agitation. The gel was stained with a staining solution (0.15% Coomassie Brilliant Blue, 50% methanol, and 10% acetic acid) and destained with a destaining solution (7.5% methanol and 7.5% acetic acid).

### Inhibition of TGF-β1 interaction between EOC and mesothelial cells

To inhibit TGF-β1–mediated interactions between EOC and mesothelial cells, we initially designed three siRNAs targeting TGF-β1 (nos. 1 to 3) with the following sequences (5′ to 3′):

1) si-TGF-β1 (no. 1): CTGTAGTTAGATCTATTTATTGA.

2) si-TGF-β1 (no. 2): CTCTGATAACACCCATTTTAAAG.

3) si-TGF-β1 (no. 3): GGGGATAGTGAAGAAGACAATAA.

As none of these siRNAs achieved significant mRNA suppression in EOC cells, we used a commercially available siRNA mix (Santa Cruz Biotechnology, sc-270322) for subsequent experiments (no. 4). OV90 and SKOV3 cells were treated with either si-TGF-β1 or si-control for 48 hours. The efficacy of si-TGF-β1 was evaluated through the examination of TGF-β1 mRNA expression via qPCR and TGF-β1 protein concentration in culture supernatants using enzyme-linked immunosorbent assay (ELISA; no. 432907, BioLegend) at days 2 and 5 after siRNA treatment. The growth ability was compared between si-TGF-β1 and si-control cells at day 2. Following siRNA treatment, EOC cells were cocultured with untreated mesothelial cells to form spheroids in a 96-well low-attachment U-bottom plate for 48 hours, after which collagen invasion assays were performed at 48 and 72 hours. To further investigate TGF-β1 signaling within ACMSs, mesothelial cells were pretreated with the TGF-β1 receptor blocker SB431542 (Fuji File, Japan) for 48 hours before spheroid formation with untreated EOC cells, and their invasive ability was similarly assessed by collagen invasion assay. For both siRNA and receptor inhibitor experiments, expansion rates compared to the original spheroid size were compared with their respective controls.

In vivo siRNA experiments were designed with a short protocol to avoid the reversal of knockdown effects beyond 1 week. EOC cells treated with si-TGF-β1 or si-control for 48 hours were injected intraperitoneally into mice. Three days after injection, abdominal fluid was collected with 1 ml of PBS, and the omentum was harvested as described above. The number of ACMSs in ascites and the metastatic area on the omentum were compared between si-TGF-β1 and si-control groups.

To inhibit Tks5 in EOC cells, we used a siRNA inhibition system (sc-25376, Santa Cruz). EOC cells were treated with si-TKS5/si-control for 48 hours before spheroid formation with mesothelial cells. We used three types of shRNAs targeting TGF-β1 (nos. 1 to 3) and nontargeting control (VectorBuilder Japan, Yokohama, Japan) with the following sequences (5′ to 3′):

1) sh-TGF-β1 (no. 1): CCACAACGAAATCTATGACAA.

2) sh-TGF-β1 (no. 2): CAAGCAGAGTACACACAGCAT.

3) sh-TGF-β1 (no. 3): ACTGCGGATCTCTGTGTCATT.

4) sh-Control (control): CCTAAGGTTAAGTCGCCCTCG.

After transduction into OV90 cells for 3 days, transduced cells were selected with puromycin (2.0 μg/ml). The efficacy of TGF-β1 knockdown was confirmed by qPCR and ELISA and compared with control cells. OV90 cells transduced with sh-TGF-β1 nos. 1 and 2 were used for subsequent in vivo experiments as a result of their effective inhibition of TGF-β1 expression. Cell proliferation was compared between sh-TGF-β1 (nos. 1 and 2) and sh-control cells at day 3. The in vivo shRNA experiments were conducted following the same short-term protocol as the siRNA experiments. A total of 1 × 10^6^ single sh-TGF-β1 (no. 1 or 2)– or sh-control–induced OV90 cells were injected intraperitoneally into mice with the same number of mesothelial cells. Three days after injection, abdominal fluid was collected using 2 ml of PBS, and omentum and peritoneal metastases were observed using a fluorescence stereomicroscope (M205FA). The number of ACMSs in ascites at 50 μl and the metastatic area on the omentum were compared between the sh-TGF-β1 and sh-control groups. Omentum metastases were further visualized using a Nikon AR X laser confocal microscopy system, and the invasive length of mesothelial cells from the cancer-mesothelial interface was measured. The concentration of TGF-β1 in ascites was determined using the ELISA kit (no. 432907, BioLegend), and differences between groups were compared statistically.

### Inhibition of fascin-1 and myosin X by the sh-knockdown system

For the shDNA expression experiment, mesothelial cells were infected with an AAV (adeno-associated virus) vector (AAV2-U6-ZsGreen1, no. 6658, Takara bio) carrying an shDNA-expressing vector. Given that this vector coexpressed the ZsGreen fluorescent protein, shDNA-expressing cells were visually distinguishable. AAV particles were introduced into mesothelial cells 3 days before spheroid formation. The designs for sh-control, sh-FSCN1, and sh-MYO10 were as follows (5′ to 3′):

1) sh-Control (control): TTCTCCGAACGTGTCACGT.

2) sh-*FSCN1* (FSCN-2706): TTGTAAGTGTCATTTGTATAACT.

3) sh-*MYO10* (MYO10-724): CGGTATAAGAGAAATCAAATA.

### Statistical analysis

All data are presented as the means ± standard error. Statistical significance between two experimental groups was analyzed using Student’s *t* test. Statistical significance of more than two groups was analyzed using a one-way analysis of variance (ANOVA) and post hoc Bonferroni test. Differences in survival trends between groups were assessed and illustrated with the log-rank test, and the HR of recurrence or death was estimated with Cox regression analysis. Statistics were evaluated with SPSS (version 29.0.1.0), and figures were illustrated with GraphPad Prism 9.0. A *P* value of <0.05 was considered statistically significant.

## Supplementary Materials

### The PDF file includes:

Figs. S1 to S13 Table S1 Legends for movies S1 to S8 Legend for supportive data S1

### Other Supplementary Material for this manuscript includes the following:

Movies S1 to S8

Supportive Data S1

## References

[1] E. Lengyel, Ovarian cancer development and metastasis. Am. J. Pathol. 177, 1053–1064 (2010). 20651229 10.2353/ajpath.2010.100105 PMC2928939

[2] H. Kobayashi, Y. Yamada, T. Sado, M. Sakata, S. Yoshida, R. Kawaguchi, S. Kanayama, H. Shigetomi, S. Haruta, Y. Tsuji, S. Ueda, T. Kitanaka, A randomized study of screening for ovarian cancer: A multicenter study in Japan. Int. J. Gynecol. Cancer 18, 414–420 (2008). 17645503 10.1111/j.1525-1438.2007.01035.x

[3] U. Menon, C. Karpinskyj, A. Gentry-Maharaj, Ovarian cancer prevention and screening. Obstet. Gynecol. 131, 909–927 (2018). 29630008 10.1097/AOG.0000000000002580

[4] I. Konishi, K. Abiko, T. Hayashi, K. Yamanoi, R. Murakami, K. Yamaguchi, J. Hamanishi, T. Baba, N. Matsumura, M. Mandai, Kyoto Study Group for Ovarian Cancer Research, Peritoneal dissemination of high-grade serous ovarian cancer: pivotal roles of chromosomal instability and epigenetic dynamics. J. Gynecol. Oncol. 33, 1–17 (2022). 10.3802/jgo.2022.33.e83 PMC9428305 36032027

[5] C. E. Ford, B. Werner, N. F. Hacker, K. Warton, The untapped potential of ascites in ovarian cancer research and treatment. Br. J. Cancer 123, 9–16 (2020). 32382112 10.1038/s41416-020-0875-x PMC7341795

[6] E. Kipps, D. S. P. Tan, S. B. Kaye, Meeting the challenge of ascites in ovarian cancer: New avenues for therapy and research. Nat. Rev. Cancer 13, 273–282 (2013). 23426401 10.1038/nrc3432 PMC4673904

[7] K. Uno, S. Iyoshi, M. Yoshihara, K. Kitami, K. Mogi, H. Fujimoto, M. Sugiyama, Y. Koya, Y. Yamakita, A. Nawa, T. Kanayama, H. Tomita, A. Enomoto, H. Kajiyama, Metastatic voyage of ovarian cancer cells in ascites with the assistance of various cellular components. Int. J. Mol. Sci. 23, 4383 (2022). 35457198 10.3390/ijms23084383 PMC9031612

[8] M.-W. Chen, S.-T. Yang, M.-H. Chien, K.-T. Hua, C.-J. Wu, S. M. Hsiao, H. Lin, M. Hsiao, J.-L. Su, L.-H. Wei, The STAT3-miRNA-92-Wnt signaling pathway regulates spheroid formation and malignant progression in ovarian cancer. Cancer Res. 77, 1955–1967 (2017). 28209618 10.1158/0008-5472.CAN-16-1115

[9] K. Shield, M. L. Ackland, N. Ahmed, G. E. Rice, Multicellular spheroids in ovarian cancer metastases: Biology and pathology. Gynecol. Oncol. 113, 143–148 (2009). 19135710 10.1016/j.ygyno.2008.11.032

[10] M. Yin, X. Li, S. Tan, H. J. Zhou, W. Ji, S. Bellone, X. Xu, H. Zhang, A. D. Santin, G. Lou, W. Min, Tumor-associated macrophages drive spheroid formation during early transcoelomic metastasis of ovarian cancer. J. Clin. Invest. 126, 4157–4173 (2016). 27721235 10.1172/JCI87252 PMC5096908

[11] I. Matte, C. M. Legault, P. Garde-Granger, C. Laplante, P. Bessette, C. Rancourt, A. Piché, Mesothelial cells interact with tumor cells for the formation of ovarian cancer multicellular spheroids in peritoneal effusions. Clin. Exp. Metastasis 33, 839–852 (2016). 27612856 10.1007/s10585-016-9821-y

[12] A. Shishido, S. Mori, Y. Yokoyama, Y. Hamada, K. Minami, Y. Qian, J. Wang, H. Hirose, X. Wu, N. Kawaguchi, S. Nagumo, N. Matsuura, H. Yamamoto, Mesothelial cells facilitate cancer stem-like properties in spheroids of ovarian cancer cells. Oncol. Rep. 40, 2105–2114 (2018). 30066911 10.3892/or.2018.6605

[13] Q. Gao, Z. Yang, S. Xu, X. Li, X. Yang, P. Jin, Y. Liu, X. Zhou, T. Zhang, C. Gong, X. Wei, D. Liu, C. Sun, G. Chen, J. Hu, L. Meng, J. Zhou, K. Sawada, R. Fruscio, T. W. Grunt, J. Wischhusen, V. M. Vargas-Hernández, B. Pothuri, R. L. Coleman, Heterotypic CAF-tumor spheroids promote early peritoneal metastatis of ovarian cancer. J. Exp. Med. 216, 688–703 (2019). 30710055 10.1084/jem.20180765 PMC6400537

[14] K. Mogi, M. Yoshihara, S. Iyoshi, K. Kitami, K. Uno, S. Tano, Y. Koya, M. Sugiyama, Y. Yamakita, A. Nawa, H. Tomita, H. Kajiyama, Ovarian cancer-associated mesothelial cells: Transdifferentiation to minions of cancer and orchestrate developing peritoneal dissemination. Cancer 13, 1–13 (2021). 10.3390/cancers13061352 PMC8002484 33802781

[15] B. Sheid, Angiogenic effects of macrophages isolated from ascitic fluid aspirated from women with advanced ovarian cancer. Cancer Lett. 62, 153–158 (1992). 1371714 10.1016/0304-3835(92)90186-y

[16] V. M. Peterson, C. M. Castro, J. Chung, N. C. Miller, A. V. Ullal, M. D. Castano, R. T. Penson, H. Lee, M. J. Birrer, R. Weissleder, Ascites analysis by a microfluidic chip allows tumor-cell profiling. Proc. Natl. Acad. Sci. U.S.A. 110, E4978–E4986 (2013). 24297935 10.1073/pnas.1315370110 PMC3870756

[17] H. Nagai, S. H. Chew, Y. Okazaki, S. Funahashi, T. Namba, T. Kato, A. Enomoto, L. Jiang, S. Akatsuka, S. Toyokuni, Metamorphosis of mesothelial cells with active horizontal motility in tissue culture. Sci. Rep. 3, 1–7 (2013). 10.1038/srep01144 PMC3556594 23359855

[18] A. J. Foley-Comer, S. E. Herrick, T. Al-Mishlab, C. M. Prêle, G. J. Laurent, S. E. Mutsaers, Evidence for incorporation of free-floating mesothelial cells as a mechanism of serosal healing. J. Cell Sci. 115, 1383–1389 (2002). 11896186 10.1242/jcs.115.7.1383

[19] I. Matte, D. Lane, D. Bachvarov, C. Rancourt, A. Piché, Role of malignant ascites on human mesothelial cells and their gene expression profiles. BMC Cancer 14, 288 (2014). 24761768 10.1186/1471-2407-14-288 PMC4008408

[20] P. Sandoval, J. A. Jiménez-Heffernan, Á. Rynne-Vidal, M. L. Pérez-Lozano, Á. Gilsanz, V. Ruiz-Carpio, R. Reyes, J. García-Bordas, K. Stamatakis, J. Dotor, P. L. Majano, M. Fresno, C. Cabañas, M. López-Cabrera, Carcinoma-associated fibroblasts derive from mesothelial cells via mesothelial-to-mesenchymal transition in peritoneal metastasis. J. Pathol. 231, 517–531 (2013). 24114721 10.1002/path.4281

[21] S. E. Mutsaers, Mesothelial cells: Their structure, function and role in serosal repair. Respirology 7, 171–191 (2002). 12153683 10.1046/j.1440-1843.2002.00404.x

[22] H. Huang, Z. Wang, Y. Zhang, R. N. Pradhan, D. Ganguly, R. Chandra, G. Murimwa, S. Wright, X. Gu, R. Maddipati, S. Müller, S. J. Turley, R. A. Brekken, Mesothelial cell-derived antigen-presenting cancer-associated fibroblasts induce expansion of regulatory T cells in pancreatic cancer. Cancer Cell 40, 656–673 (2022). 35523176 10.1016/j.ccell.2022.04.011 PMC9197998

[23] S. E. Mutsaers, C. M.-A. Prêle, S. Pengelly, S. E. Herrick, Mesothelial cells and peritoneal homeostasis. Fertil. Steril. 106, 1018–1024 (2016). 27692285 10.1016/j.fertnstert.2016.09.005

[24] A. Higashiguchi, T. Yamada, N. Susumu, T. Mori, A. Suzuki, D. Aoki, M. Sakamoto, Specific expression of hepatocyte nuclear factor-1β in the ovarian clear cell adenocarcinoma and its application to cytological diagnosis. Cancer Sci. 98, 387–391 (2007). 17270029 10.1111/j.1349-7006.2007.00398.x PMC11159962

[25] F. Hasteh, G. Y. Lin, N. Weidner, C. W. Michael, The use of immunohistochemistry to distinguish reactive mesothelial cells from malignant mesothelioma in cytologic effusions. Cancer Cytopathol. 118, 90–96 (2010). 20209622 10.1002/cncy.20071

[26] A. Yonemura, T. Semba, J. Zhang, Y. Fan, N. Yasuda-Yoshihara, H. Wang, T. Uchihara, T. Yasuda, A. Nishimura, L. Fu, X. Hu, F. Wei, F. Kitamura, T. Akiyama, K. Yamashita, K. Eto, S. Iwagami, M. Iwatsuki, Y. Miyamoto, K. Matsusaki, J. Yamasaki, O. Nagano, H. Saya, S. Song, P. Tan, H. Baba, J. A. Ajani, T. Ishimoto, Mesothelial cells with mesenchymal features enhance peritoneal dissemination by forming a protumorigenic microenvironment. Cell Rep. 43, 113613 (2024). 38232734 10.1016/j.celrep.2023.113613

[27] O. Lin, Challenges in the interpretation of peritoneal cytologic specimens. Arch. Pathol. Lab. Med. 133, 739–742 (2009). 19415948 10.5858/133.5.739

[28] A. Sato, I. Torii, Y. Okamura, T. Yamamoto, T. Nishigami, T. R. Kataoka, M. Song, S. Hasegawa, T. Nakano, T. Kamei, T. Tsujimura, Immunocytochemistry of CD146 is useful to discriminate between malignant pleural mesothelioma and reactive mesothelium. Mod. Pathol. 23, 1458–1466 (2010). 20657552 10.1038/modpathol.2010.134

[29] H. Batra, V. B. Antony, The pleural mesothelium in development and disease. Front. Physiol. 5, 284 (2014). 25136318 10.3389/fphys.2014.00284 PMC4117979

[30] K. Ojasalu, C. Brehm, K. Hartung, M. Nischak, F. Finkernagel, P. Rexin, A. Nist, E. Pavlakis, T. Stiewe, J. M. Jansen, U. Wagner, S. Gattenlöhner, A. Bräuninger, S. Müller-Brüsselbach, S. Reinartz, R. Müller, Upregulation of mesothelial genes in ovarian carcinoma cells is associated with an unfavorable clinical outcome and the promotion of cancer cell adhesion. Mol. Oncol. 14, 2142–2162 (2020). 32533757 10.1002/1878-0261.12749 PMC7463315

[31] M. Yoshihara, H. Kajiyama, A. Yokoi, M. Sugiyama, Y. Koya, Y. Yamakita, W. Liu, K. Nakamura, Y. Moriyama, H. Yasui, S. Suzuki, Y. Yamamoto, C. Ricciardelli, A. Nawa, K. Shibata, F. Kikkawa, Ovarian cancer-associated mesothelial cells induce acquired platinum-resistance in peritoneal metastasis via the FN1/Akt signaling pathway. Int. J. Cancer 146, 2268–2280 (2020). 31904865 10.1002/ijc.32854 PMC7065188

[32] S. Linder, P. Cervero, R. Eddy, J. Condeelis, Mechanisms and roles of podosomes and invadopodia. Nat. Rev. Mol. Cell Biol. 24, 86–106 (2023). 36104625 10.1038/s41580-022-00530-6

[33] J. Pu, Y. Huang, Q. Fang, J. Wang, W. Li, Z. Xu, X. Wu, Y. Lu, H. Wei, Hypoxia-induced Fascin-1 upregulation is regulated by Akt/Rac1 axis and enhances malignant properties of liver cancer cells via mediating actin cytoskeleton rearrangement and Hippo/YAP activation. Cell Death Discov. 7, 385 (2021). 34897283 10.1038/s41420-021-00778-5 PMC8665929

[34] A. Li, J. P. Morton, Y. F. Ma, S. A. Karim, Y. Zhou, W. J. Faller, E. F. Woodham, H. T. Morris, R. P. Stevenson, A. Juin, N. B. Jamieson, C. J. Mac Kay, C. R. Carter, H. Y. Leung, S. Yamashiro, K. Blyth, O. J. Sansom, L. M. Machesky, Fascin is regulated by slug, promotes progression of pancreatic cancer in mice, and is associated with patient outcomes. Gastroenterology 146, 1386–1396 (2014). 24462734 10.1053/j.gastro.2014.01.046 PMC4000441

[35] R. Satoyoshi, N. Aiba, K. Yanagihara, M. Yashiro, M. Tanaka, Tks5 activation in mesothelial cells creates invasion front of peritoneal carcinomatosis. Oncogene 34, 3176–3187 (2015). 25088196 10.1038/onc.2014.246

[36] B. Izar, I. Tirosh, E. H. Stover, I. Wakiro, M. S. Cuoco, I. Alter, C. Rodman, R. Leeson, M.-J. Su, P. Shah, M. Iwanicki, S. R. Walker, A. Kanodia, J. C. Melms, S. Mei, J.-R. Lin, C. B. M. Porter, M. Slyper, J. Waldman, L. Jerby-Arnon, O. Ashenberg, T. J. Brinker, C. Mills, M. Rogava, S. Vigneau, P. K. Sorger, L. A. Garraway, P. A. Konstantinopoulos, J. F. Liu, U. Matulonis, B. E. Johnson, O. Rozenblatt-Rosen, A. Rotem, A. Regev, A single-cell landscape of high-grade serous ovarian cancer. Nat. Med. 26, 1271–1279 (2020). 32572264 10.1038/s41591-020-0926-0 PMC7723336

[37] I. Vázquez-García, F. Uhlitz, N. Ceglia, J. L. P. Lim, M. Wu, N. Mohibullah, J. Niyazov, A. E. B. Ruiz, K. M. Boehm, V. Bojilova, C. J. Fong, T. Funnell, D. Grewal, E. Havasov, S. Leung, A. Pasha, D. M. Patel, M. Pourmaleki, N. Rusk, H. Shi, R. Vanguri, M. J. Williams, A. W. Zhang, V. Broach, D. S. Chi, A. D. C. Paula, G. J. Gardner, S. H. Kim, M. Lennon, K. L. Roche, Y. Sonoda, O. Zivanovic, R. Kundra, A. Viale, F. N. Derakhshan, L. Geneslaw, S. I. Bhaloo, A. Maroldi, R. Nunez, F. Pareja, A. Stylianou, M. Vahdatinia, Y. Bykov, R. N. Grisham, Y. L. Liu, Y. Lakhman, I. Nikolovski, D. Kelly, J. Gao, A. Schietinger, T. J. Hollmann, S. F. Bakhoum, R. A. Soslow, L. H. Ellenson, N. R. Abu-Rustum, C. Aghajanian, C. F. Friedman, A. M. Pherson, B. Weigelt, D. Zamarin, S. P. Shah, Ovarian cancer mutational processes drive site-specific immune evasion. Nature 612, 778–786 (2022). 36517593 10.1038/s41586-022-05496-1 PMC9771812

[38] C. X. Dominguez, S. Müller, S. Keerthivasan, H. Koeppen, J. Hung, S. Gierke, B. Breart, O. Foreman, T. W. Bainbridge, A. Castiglioni, Y. Senbabaoglu, Z. Modrusan, Y. Liang, M. R. Junttila, C. Klijn, R. Bourgon, S. J. Turley, Single-cell RNA sequencing reveals stromal evolution into LRRC15^+^ myofibroblasts as a determinant of patient response to cancer immunotherapy. Cancer Discov. 10, 232–253 (2020). 31699795 10.1158/2159-8290.CD-19-0644

[39] A. J. Shih, A. Menzin, J. Whyte, J. Lovecchio, A. Liew, H. Khalili, T. Bhuiya, P. K. Gregersen, A. T. Lee, Identification of grade and origin specific cell populations in serous epithelial ovarian cancer by single cell RNA-seq. PLOS ONE 13, 1–17 (2018). 10.1371/journal.pone.0206785 PMC6211742 30383866

[40] X. Zheng, X. Wang, X. Cheng, Z. Liu, Y. Yin, X. Li, Z. Huang, Z. Wang, W. Guo, F. Ginhoux, Z. Li, Z. Zhang, X. Wang, Single-cell analyses implicate ascites in remodeling the ecosystems of primary and metastatic tumors in ovarian cancer. Nat. Cancer 4, 1138–1156 (2023). 37488416 10.1038/s43018-023-00599-8 PMC10447252

[41] J. Obacz, J. A. Valer, R. Nibhani, T. S. Adams, J. C. Schupp, N. Veale, A. Lewis-Wade, J. Flint, J. Hogan, G. Aresu, A. S. Coonar, A. Peryt, G. Biffi, N. Kaminski, H. Francies, D. M. Rassl, M. J. Garnett, R. C. Rintoul, S. J. Marciniak, Single-cell transcriptomic analysis of human pleura reveals stromal heterogeneity and informs in vitro models of mesothelioma. Eur. Respir. J. 63, 2300143 (2024). 38212075 10.1183/13993003.00143-2023 PMC10809128

[42] C. Pesenti, L. Beltrame, A. Velle, R. Fruscio, M. Jaconi, F. Borella, F. M. Cribiù, E. Calura, L. V. Venturini, D. Lenoci, F. Agostinis, D. Katsaros, N. Panini, T. Bianchi, F. Landoni, M. Miozzo, M. D’Incalci, J. D. Brenton, C. Romualdi, S. Marchini, Copy number alterations in stage I epithelial ovarian cancer highlight three genomic patterns associated with prognosis. Eur. J. Cancer 171, 85–95 (2022). 35714451 10.1016/j.ejca.2022.05.005

[43] J. Chien, H. Sicotte, J.-B. Fan, S. Humphray, J. M. Cunningham, K. R. Kalli, A. L. Oberg, S. N. Hart, Y. Li, J. I. Davila, S. Baheti, C. Wang, S. Dietmann, E. J. Atkinson, Y. W. Asmann, D. A. Bell, T. Ota, Y. Tarabishy, R. Kuang, M. Bibikova, R. K. Cheetham, R. J. Grocock, E. M. Swisher, J. Peden, D. Bentley, J.-P. A. Kocher, S. H. Kaufmann, L. C. Hartmann, V. Shridhar, E. L. Goode, *TP53* mutations, tetraploidy and homologous recombination repair defects in early stage high-grade serous ovarian cancer. Nucleic Acids Res. 43, 6945–6958 (2015). 25916844 10.1093/nar/gkv111 PMC4538798

[44] M. A. Eckert, S. Pan, K. M. Hernandez, R. M. Loth, J. Andrade, S. L. Volchenboum, P. Faber, A. Montag, R. Lastra, M. E. Peter, S. D. Yamada, E. Lengyel, Genomics of ovarian cancer progression reveals diverse metastatic trajectories including intraepithelial metastasis to the fallopian tube. Cancer Discov. 6, 1342–1351 (2016). 27856443 10.1158/2159-8290.CD-16-0607 PMC5164915

[45] A. Bashashati, G. Ha, A. Tone, J. Ding, L. M. Prentice, A. Roth, J. Rosner, K. Shumansky, S. Kalloger, J. Senz, W. Yang, M. M. Conechy, N. Melnyk, M. Anglesio, M. T. Y. Luk, K. Tse, T. Zeng, R. Moore, Y. Zhao, M. A. Marra, B. Gilks, S. Yip, D. G. Huntsman, J. N. Mc Alpine, S. P. Shah, Distinct evolutionary trajectories of primary high-grade serous ovarian cancers revealed through spatial mutational profiling. J. Pathol. 231, 21–34 (2013). 23780408 10.1002/path.4230 PMC3864404

[46] B. M. Barnes, L. Nelson, A. Tighe, G. J. Burghel, I.-H. Lin, S. Desai, J. C. M. Grail, R. D. Morgan, S. S. Taylor, Distinct transcriptional programs stratify ovarian cancer cell lines into the five major histological subtypes. Genome Med. 13, 1–19 (2021). 34470661 10.1186/s13073-021-00952-5 PMC8408985

[47] T. G. Shepherd, B. L. Thériault, E. J. Campbell, M. W. Nachtigal, Primary culture of ovarian surface epithelial cells and ascites-derived ovarian cancer cells from patients. Nat. Protoc. 1, 2643–2649 (2007). 10.1038/nprot.2006.328 17406520

[48] T. P. Wilm, H. Tanton, F. Mutter, V. Foisor, B. Middlehurst, K. Ward, T. Benameur, N. Hastie, B. Wilm, Restricted differentiative capacity of Wt1-expressing peritoneal mesothelium in postnatal and adult mice. Sci. Rep. 11, 1–15 (2021). 34354169 10.1038/s41598-021-95380-1 PMC8342433

[49] K. Shield, C. Riley, M. A. Quinn, G. E. Rice, M. L. Ackland, N. Ahmed, Α2Β1 integrin affects metastatic potential of ovarian carcinoma spheroids by supporting disaggregation and proteolysis. J. Carcinog. 6, 11 (2007). 17567918 10.1186/1477-3163-6-11 PMC1929068

[50] M. Tanaka, Crosstalk of tumor stromal cells orchestrates invasion and spreading of gastric cancer. Pathol. Int. 72, 219–233 (2022). 35112770 10.1111/pin.13211

[51] B. T. Beaty, J. Condeelis, Digging a little deeper: The stages of invadopodium formation and maturation. Eur. J. Cell Biol. 93, 438–444 (2014). 25113547 10.1016/j.ejcb.2014.07.003 PMC4262566

[52] S. Natarajan, K. M. Foreman, M. I. Soriano, N. S. Rossen, H. Shehade, D. R. Fregoso, J. T. Eggold, V. Krishnan, O. Dorigo, A. J. Krieg, S. C. Heilshorn, S. Sinha, K. C. Fuh, E. B. Rankin, Collagen remodeling in the hypoxic tumor-mesothelial niche promotes ovarian cancer metastasis. Cancer Res. 79, 2271–2284 (2019). 30862717 10.1158/0008-5472.CAN-18-2616 PMC6822898

[53] X. Zhao, S. Gao, H. Ren, W. Sun, H. Zhang, J. Sun, S. Yang, J. Hao, Hypoxia-inducible factor-1 promotes pancreatic ductal adenocarcinoma invasion and metastasis by activating transcription of the actin-bundling protein fascin. Cancer Res. 74, 2455–2464 (2014). 24599125 10.1158/0008-5472.CAN-13-3009

[54] D. A. Murphy, S. A. Courtneidge, The “ins” and “outs” of podosomes and invadopodia: Characteristics, formation and function. Nat. Rev. Mol. Cell Biol. 12, 413–426 (2011). 21697900 10.1038/nrm3141 PMC3423958

[55] H. Zhang, J. S. Berg, Z. Li, Y. Wang, P. Lång, A. D. Sousa, A. Bhaskar, R. E. Cheney, S. Strömblad, Myosin-X provides a motor-based link between integrins and the cytoskeleton. Nat. Cell Biol. 6, 523–531 (2004). 15156152 10.1038/ncb1136

[56] S. Meng, E. E. Sørensen, M. Ponniah, J. Thorlacius-Ussing, R. Crouigneau, T. Larsen, M. T. Borre, N. Willumsen, M. Flinck, S. F. Pedersen, MCT4 and CD147 colocalize with MMP14 in invadopodia and support matrix degradation and invasion by breast cancer cells. J. Cell Sci. 137, 261608 (2024). 10.1242/jcs.261608 PMC11112124 38661040

[57] N. M. Moss, M. V. Barbolina, Y. Liu, L. Sun, H. G. Munshi, M. S. Stack, Ovarian cancer cell detachment and multicellular aggregate formation are regulated by membrane type 1 matrix metalloproteinase: A potential role in I.p. metastatic dissemination. Cancer Res. 69, 7121–7129 (2009). 19706774 10.1158/0008-5472.CAN-08-4151 PMC2737080

[58] S. M. Guire, B. Kara, P. C. Hart, A. Montag, K. Wroblewski, S. Fazal, X.-Y. Huang, E. Lengyel, H. A. Kenny, Inhibition of fascin in cancer and stromal cells blocks ovarian cancer metastasis. Gynecol. Oncol. 153, 405–415 (2019). 30797592 10.1016/j.ygyno.2019.01.020 PMC6486884

[59] T. Yasuda, M. Koiwa, A. Yonemura, K. Miyake, R. Kariya, S. Kubota, T. Yokomizo-Nakano, N. Yasuda-Yoshihara, T. Uchihara, R. Itoyama, L. Bu, L. Fu, K. Arima, D. Izumi, S. Iwagami, K. Eto, M. Iwatsuki, Y. Baba, N. Yoshida, H. Ohguchi, S. Okada, K. Matsusaki, G. Sashida, A. Takahashi, P. Tan, H. Baba, T. Ishimoto, Inflammation-driven senescence-associated secretory phenotype in cancer-associated fibroblasts enhances peritoneal dissemination. Cell Rep. 34, 108779 (2021). 33626356 10.1016/j.celrep.2021.108779

[60] M. Slyper, C. B. M. Porter, O. Ashenberg, J. Waldman, E. Drokhlyansky, I. Wakiro, C. Smillie, G. Smith-Rosario, J. Wu, D. Dionne, S. Vigneau, J. Jané-Valbuena, T. L. Tickle, S. Napolitano, M.-J. Su, A. G. Patel, A. Karlstrom, S. Gritsch, M. Nomura, A. Waghray, S. H. Gohil, A. M. Tsankov, L. Jerby-Arnon, O. Cohen, J. Klughammer, Y. Rosen, J. Gould, L. Nguyen, M. Hofree, P. J. Tramontozzi, B. Li, C. J. Wu, B. Izar, R. Haq, F. S. Hodi, C. H. Yoon, A. N. Hata, S. J. Baker, M. L. Suvà, R. Bueno, E. H. Stover, M. R. Clay, M. A. Dyer, N. B. Collins, U. A. Matulonis, N. Wagle, B. E. Johnson, A. Rotem, O. Rozenblatt-Rosen, A. Regev, A single-cell and single-nucleus RNA-Seq toolbox for fresh and frozen human tumors. Nat. Med. 26, 792–802 (2020). 32405060 10.1038/s41591-020-0844-1 PMC7220853

[61] L. G. Koss, M. R. Melamed, *Koss’ Diagnostic Cytology and Its Histopathologic Bases* (Lippincott Williams & Wilkins, ed. 5, 2005).

[62] M. Bartoschek, N. Oskolkov, M. Bocci, J. Lövrot, C. Larsson, M. Sommarin, C. D. Madsen, D. Lindgren, G. Pekar, G. Karlsson, M. Ringnér, J. Bergh, Å. Björklund, K. Pietras, Spatially and functionally distinct subclasses of breast cancer-associated fibroblasts revealed by single cell RNA sequencing. Nat. Commun. 9, 5150 (2018). 30514914 10.1038/s41467-018-07582-3 PMC6279758

[63] A. Ghoneum, S. Almousa, B. Warren, A. Y. Abdulfattah, J. Shu, H. Abouelfadl, D. Gonzalez, C. Livingston, N. Said, Exploring the clinical value of tumor microenvironment in platinum-resistant ovarian cancer. Semin. Cancer Biol. 77, 83–98 (2021). 33476723 10.1016/j.semcancer.2020.12.024 PMC8286277

[64] B. P. Rickard, C. Conrad, A. J. Sorrin, M. K. Ruhi, J. C. Reader, S. A. Huang, W. Franco, G. Scarcelli, W. J. Polacheck, D. M. Roque, M. G. Del Carmen, H.-C. Huang, U. Demirci, I. Rizvi, Malignant ascites in ovarian cancer: Cellular, acellular, and biophysical determinants of molecular characteristics and therapy response. Cancer 13, 134318 (2021). 10.3390/cancers13174318 PMC8430600 34503128

[65] M. P. Iwanicki, R. A. Davidowitz, M. R. Ng, A. Besser, T. Muranen, M. Merritt, G. Danuser, T. A. Ince, J. S. Brugge, Ovarian cancer spheroids use myosin-generated force to clear the mesothelium. Cancer Discov. 1, 144–157 (2011). 22303516 10.1158/2159-8274.CD-11-0010 PMC3269166

[66] G. Gunay, H. A. Kirit, A. Kamatar, O. Baghdasaryan, S. Hamsici, H. Acar, The effects of size and shape of the ovarian cancer spheroids on the drug resistance and migration. Gynecol. Oncol. 159, 563–572 (2020). 32958270 10.1016/j.ygyno.2020.09.002

[67] D. Yan, X. Liu, H. Xu, S.-W. Guo, Mesothelial cells participate in endometriosis fibrogenesis through platelet-induced mesothelial-mesenchymal transition. J. Clin. Endocrinol. Metab. 105, 1–24 (2020). 32813013 10.1210/clinem/dgaa550

[68] K. L. Sodek, M. J. Ringuette, T. J. Brown, Compact spheroid formation by ovarian cancer cells is associated with contractile behavior and an invasive phenotype. Int. J. Cancer 124, 2060–2070 (2009). 19132753 10.1002/ijc.24188

[69] C. Gaggioli, S. Hooper, C. Hidalgo-Carcedo, R. Grosse, J. F. Marshall, K. Harrington, E. Sahai, Fibroblast-led collective invasion of carcinoma cells with differing roles for RhoGTPases in leading and following cells. Nat. Cell Biol. 9, 1392–1400 (2007). 18037882 10.1038/ncb1658

[70] K. Kitami, M. Yoshihara, S. Tamauchi, M. Sugiyama, Y. Koya, Y. Yamakita, H. Fujimoto, S. Iyoshi, K. Uno, K. Mogi, Y. Ikeda, A. Yokoi, N. Yoshikawa, K. Nishino, K. Niimi, A. Nawa, A. Enomoto, H. Kajiyama, Peritoneal restoration by repurposing vitamin D inhibits ovarian cancer dissemination via blockade of the TGF-β1/thrombospondin-1 axis. Matrix Biol. 109, 70–90 (2022). 35339636 10.1016/j.matbio.2022.03.003

[71] A. Hussain, V. Voisin, S. Poon, C. Karamboulas, N. H. B. Bui, J. Meens, J. Dmytryshyn, V. W. Ho, K. H. Tang, J. Paterson, B. A. Clarke, M. Q. Bernardini, G. D. Bader, B. G. Neel, L. E. Ailles, Distinct fibroblast functional states drive clinical outcomes in ovarian cancer and are regulated by tcf21. J. Exp. Med. 217, e20191094 (2020). 32434219 10.1084/jem.20191094 PMC7398174

[72] Y. Klymenko, O. Kim, E. Loughran, J. Yang, R. Lombard, M. Alber, M. S. Stack, Cadherin composition and multicellular aggregate invasion in organotypic models of epithelial ovarian cancer intraperitoneal metastasis. Oncogene 36, 5840–5851 (2017). 28628116 10.1038/onc.2017.171 PMC5648607

[73] Y. Peng, H. Kajiyama, H. Yuan, K. Nakamura, M. Yoshihara, A. Yokoi, K. Fujikake, H. Yasui, N. Yoshikawa, S. Suzuki, T. Senga, K. Shibata, F. Kikkawa, PAI-1 secreted from metastatic ovarian cancer cells triggers the tumor-promoting role of the mesothelium in a feedback loop to accelerate peritoneal dissemination. Cancer Lett. 442, 181–192 (2019). 30429105 10.1016/j.canlet.2018.10.027

[74] J. Liao, F. Qian, N. Tchabo, P. Mhawech-Fauceglia, A. Beck, Z. Qian, X. Wang, W. J. Huss, S. B. Lele, C. D. Morrison, K. Odunsi, Ovarian cancer spheroid cells with stem cell-like properties contribute to tumor generation, metastasis and chemotherapy resistance through hypoxia-resistant metabolism. PLOS ONE 9, 1–13 (2014). 10.1371/journal.pone.0084941 PMC3883678 24409314

[75] M. Chauvin, M.-C. Meinsohn, S. Dasari, P. May, S. Iyer, N. M. P. Nguyen, E. Oliva, Z. Lucchini, N. Nagykery, A. Kashiwagi, R. Mishra, R. Maser, J. Wells, C. J. Bult, A. K. Mitra, P. K. Donahoe, D. Pépin, Cancer-associated mesothelial cells are regulated by the anti-Müllerian hormone axis. Cell Rep. 42, 112730 (2023). 37453057 10.1016/j.celrep.2023.112730

[76] C. Li, E. Bonazzoli, S. Bellone, J. Choi, W. Dong, G. Menderes, G. Altwerger, C. Han, A. Manzano, A. Bianchi, F. Pettinella, P. Manara, S. Lopez, G. Yadav, F. Riccio, L. Zammataro, B. Zeybek, Y. Yang-Hartwich, N. Buza, P. Hui, S. Wong, A. Ravaggi, E. Bignotti, C. Romani, P. Todeschini, L. Zanotti, V. Zizioli, F. Odicino, S. Pecorelli, L. Ardighieri, D.-A. Silasi, B. Litkouhi, E. Ratner, M. Azodi, G. S. Huang, P. E. Schwartz, R. P. Lifton, J. Schlessinger, A. D. Santin, Mutational landscape of primary, metastatic, and recurrent ovarian cancer reveals c-MYC gains as potential target for BET inhibitors. Proc. Natl. Acad. Sci. U.S.A. 116, 619–624 (2019). 30584090 10.1073/pnas.1814027116 PMC6329978

[77] S. Zhu, M. Zhang, X. Liu, Q. Luo, J. Zhou, M. Song, J. Feng, J. Liu, Single-cell transcriptomics provide insight into metastasis-related subsets of breast cancer. Breast Cancer Res. 25, 1–9 (2023). 37858183 10.1186/s13058-023-01728-y PMC10588105

[78] S. Yachida, S. Jones, I. Bozic, T. Antal, R. Leary, B. Fu, M. Kamiyama, R. H. Hruban, J. R. Eshleman, M. A. Nowak, V. E. Velculescu, K. W. Kinzler, B. Vogelstein, C. A. Iacobuzio-Donahue, Distant metastasis occurs late during the genetic evolution of pancreatic cancer. Nature 467, 1114–1117 (2010). 20981102 10.1038/nature09515 PMC3148940

[79] M. Lodyga, E. Cambridge, H. M. Karvonen, P. Pakshir, B. Wu, S. Boo, M. Kiebalo, R. Kaarteenaho, M. Glogauer, M. Kapoor, K. Ask, B. Hinz, Cadherin-11-mediated adhesion of macrophages to myofibroblasts establishes a profibrotic niche of active TGF-β. Sci. Signal. 12, eaao3496 (2019). 10.1126/scisignal.aao3469 30647145

[80] E. Sarantelli, A. Mourkakis, L. C. Zacharia, A. Stylianou, V. Gkretsi, Fascin-1 in cancer cell metastasis: Old target-new insights. Int. J. Mol. Sci. 24, 11253 (2023). 37511011 10.3390/ijms241411253 PMC10379093

[81] H. Kajiyama, F. Kikkawa, O. Maeda, T. Suzuki, K. Ino, S. Mizutani, Increased expression of dipeptidyl peptidase IV in human mesothelial cells by malignant ascites from ovarian carcinoma patients. Oncology 63, 158–165 (2002). 12239451 10.1159/000063801

[82] E. Blanco-Carmona, Generating publication ready visualizations for Single Cell transcriptomics using SCpubr. bioRxiv 482303 [Preprint] (2022).

[83] M. Schubert, B. Klinger, M. Klünemann, A. Sieber, F. Uhlitz, S. Sauer, M. J. Garnett, N. Blüthgen, J. Saez-Rodriguez, Perturbation-response genes reveal signaling footprints in cancer gene expression. Nat. Commun. 9, 20 (2018). 29295995 10.1038/s41467-017-02391-6 PMC5750219

[84] Z. Gu, R. Eils, M. Schlesner, Complex heatmaps reveal patterns and correlations in multidimensional genomic data. Bioinformatics 32, 2847–2849 (2016). 27207943 10.1093/bioinformatics/btw313

[85] F. Rohart, B. Gautier, A. Singh, K.-A. L. Cao, mixOmics: An R package for ‘omics feature selection and multiple data integration. PLoS Comput. Biol. 13, e1005752 (2017). 29099853 10.1371/journal.pcbi.1005752 PMC5687754

[86] M. E. Ritchie, B. Phipson, D. Wu, Y. Hu, C. W. Law, W. Shi, G. K. Smyth, Limma powers differential expression analyses for RNA-sequencing and microarray studies. Nucleic Acids Res. 43, e47 (2015). 25605792 10.1093/nar/gkv007 PMC4402510

[87] T. Wu, E. Hu, S. Xu, M. Chen, P. Guo, Z. Dai, T. Feng, L. Zhou, W. Tang, L. Zhan, X. Fu, S. Liu, X. Bo, G. Yu, clusterProfiler 4.0: A universal enrichment tool for interpreting omics data. Innovation 2, 100141 (2021). 34557778 10.1016/j.xinn.2021.100141 PMC8454663

[88] K. Uno, Y. Koya, M. Yoshihara, S. Iyoshi, K. Kitami, M. Sugiyama, E. Miyamoto, K. Mogi, H. Fujimoto, Y. Yamakita, X. Wang, A. Nawa, H. Kajiyama, Chondroitin sulfate proteoglycan 4 provides new treatment approach to preventing peritoneal dissemination in ovarian cancer. Int. J. Mol. Sci. 25, 1626 (2024). 38338902 10.3390/ijms25031626 PMC10855983

