# Supplementary material for: Mesothelial cells promote peritoneal invasion and metastasis of ascites-derived ovarian cancer cells through spheroid formation
Source: Sci Adv. 2026 Feb 6;12(6):eadu5944. doi: 10.1126/sciadv.adu5944 (PMC12880548; doi:10.1126/sciadv.adu5944)
Supplement: Supplementary file 1 — Figs. S1 to S13 Table S1 Legends for movies S1 to S8 Legend for supportive data S1 [file sciadv.adu5944_sm.pdf]

Science Advances
AAAS

# Supplementary Materials for

## Mesothelial cells promote peritoneal invasion and metastasis of ascites-derived ovarian cancer cells through spheroid formation

Kaname Uno *et al.*

Corresponding author: Kaname Uno, kaname.uno@med.lu.se;
Masato Yoshihara, myoshihara1209@med.nagoya-u.ac.jp

*Sci. Adv.* **12**, eadu5944 (2026)
DOI: 10.1126/sciadv.adu5944

**The PDF file includes:**

Figs. S1 to S13
Table S1
Legends for movies S1 to S8
Legend for supportive data S1

**Other Supplementary Material for this manuscript includes the following:**

Movies S1 to S8
Supportive Data S1

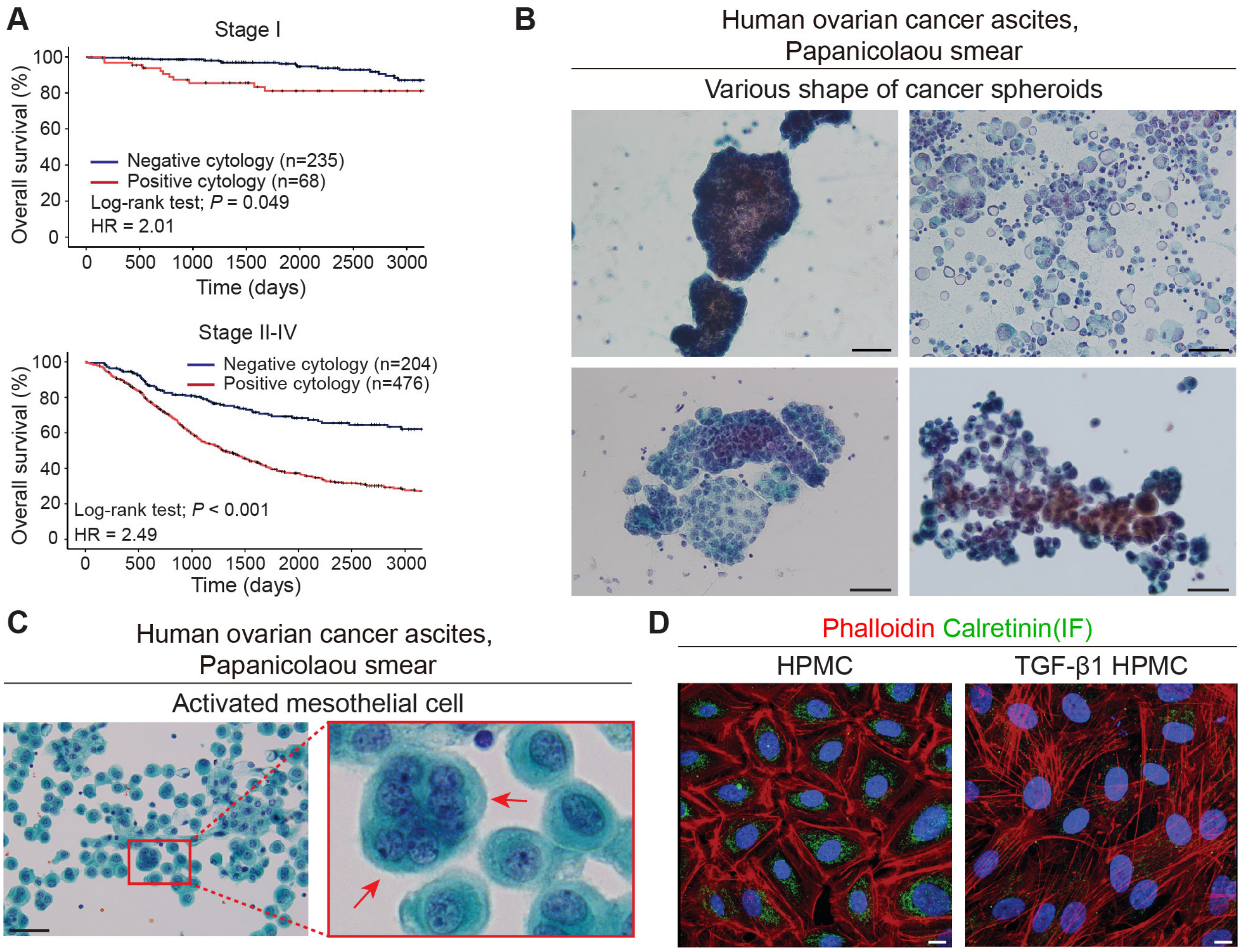


**Fig. S1. Various ovarian cancer spheroids and staining patterns of EOC and mesothelial cells.** (A) Kaplan–Meier curves for overall survival in patients with stage I (n=303) and stage II/III/IV (n=680) ovarian cancer. Data were obtained from the Tokai Ovarian Cancer Group; patients with positive cytology at the time of initial surgery showed a significantly worse prognosis in both groups. Positive cytology indicates the existence of EOC cells in ascites. (B) Papanicolaou staining results from different patients. The size and shapes of spheroids were different in each patient, but almost all EOC cells in ascites were present as spheroids. Bar = 50 µm. (C) Papanicolaou staining results of activated mesothelial cells in clinical ascites sample. These cells have multiple nuclei, and their morphology is similar to that of EOC cells (red arrows). Bar = 50 µm. (D) Immunofluorescence images with calretinin staining. The morphology and expression of calretinin differed in the mesothelial cells with and without TGF-β1 stimulation. Bar = 8 µm. HR, hazard ratio; EOC, epithelial ovarian cancer; IF, Immunofluorescence.

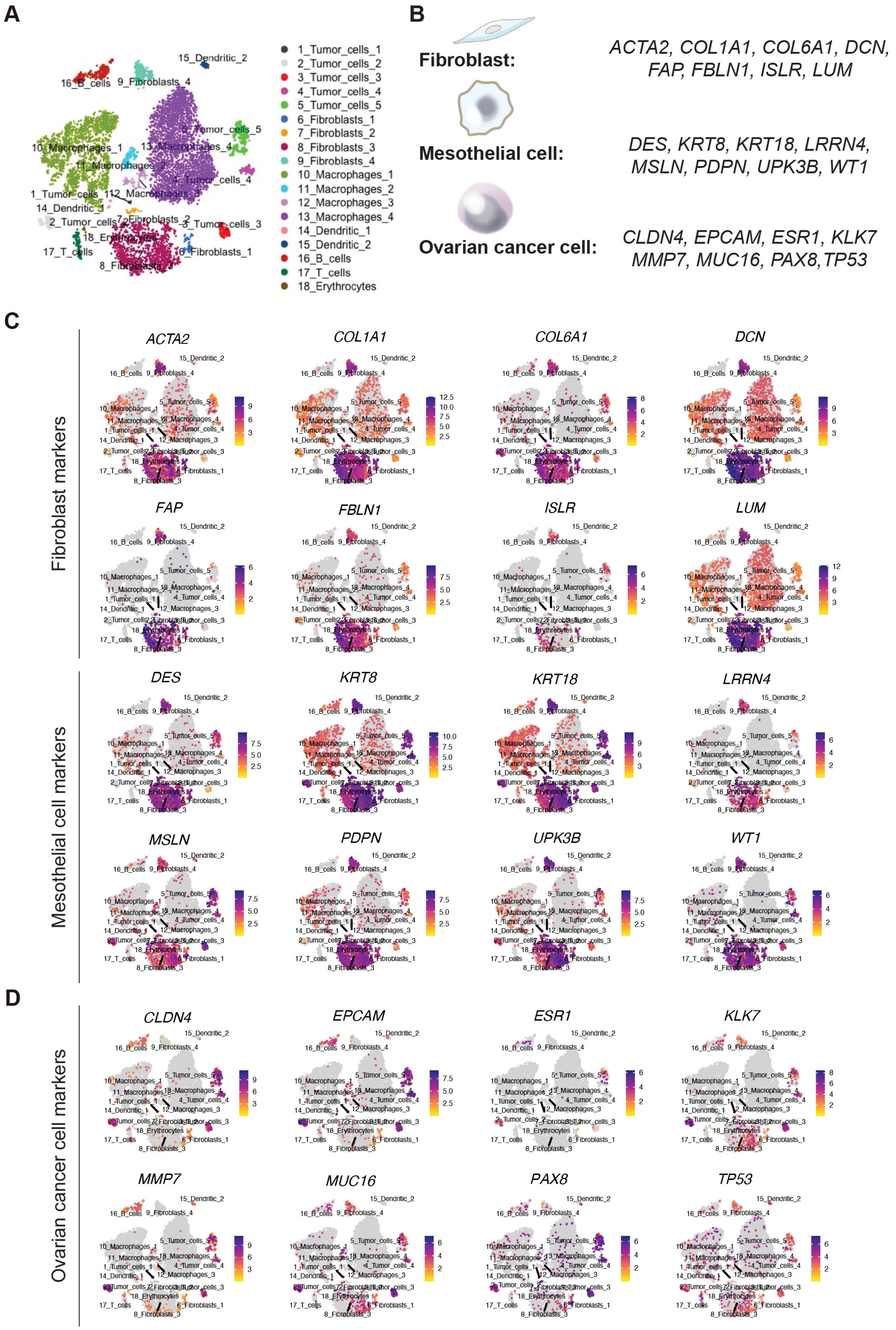
A
1_Tumor_cells_1
2_Tumor_cells_2
3_Tumor_cells_3
4_Tumor_cells_4
5_Tumor_cells_5
6_Fibroblasts_1
7_Fibroblasts_2
8_Fibroblasts_3
9_Fibroblasts_4
10_Macrophages_1
11_Macrophages_2
12_Macrophages_3
13_Macrophages_4
14_Dendritic_1
15_Dendritic_2
16_B_cells
17_T_cells
18_Erythrocytes
B
Fibroblast:
ACTA2, COL1A1, COL6A1, DCN, FAP, FBLN1, ISLR, LUM
Mesothelial cell:
DES, KRT8, KRT18, LRRN4, MSLN, PDPN, UPK3B, WT1
Ovarian cancer cell:
CLDN4, EPCAM, ESR1, KLK7 MMP7, MUC16, PAX8,TP53
C
Fibroblast markers
ACTA2
COL1A1
COL6A1
DCN
FAP
FBLN1
ISLR
LUM
Mesothelial cell markers
DES
KRT8
KRT18
LRRN4
MSLN
PDPN
UPK3B
WT1
D
Ovarian cancer cell markers
CLDN4
EPCAM
ESR1
KLK7
MMP7
MUC16
PAX8
TP53

**Fig. S2. The previous classification of "fibroblasts" in the single-cell RNA-sequencing data clearly represented a mixture of both fibroblasts and mesothelial cells.** (A) Reproduction of t-SNE-based cellular classification using previously published single-cell RNA-sequencing data. Cluster 6–9 were annotated as "fibroblasts" in the original study. (B) The specific markers used for identification and characterization of mesothelial cells, fibroblasts, and EOC cells in ascites. (C, D) t-SNE plots showing the RNA expression of (C) mesothelial cells and fibroblasts, and (D) EOC cell marker genes. The clusters 6–9, previously annotate as "fibroblasts" did express classic fibroblast marker genes (e.g. *COL1A1, DCN*, and *ACTA2*) and mesothelial-specific marker genes (e.g. *DES, LRRN4, UPK3B, MSLN*, and *WT1*). These results indicate that the "fibroblasts" cluster, in fact, were clearly a mix between fibroblasts and mesothelial cells. t-SNE, t-distributed stochastic neighbor embedding. EOC, epithelial ovarian cancer.

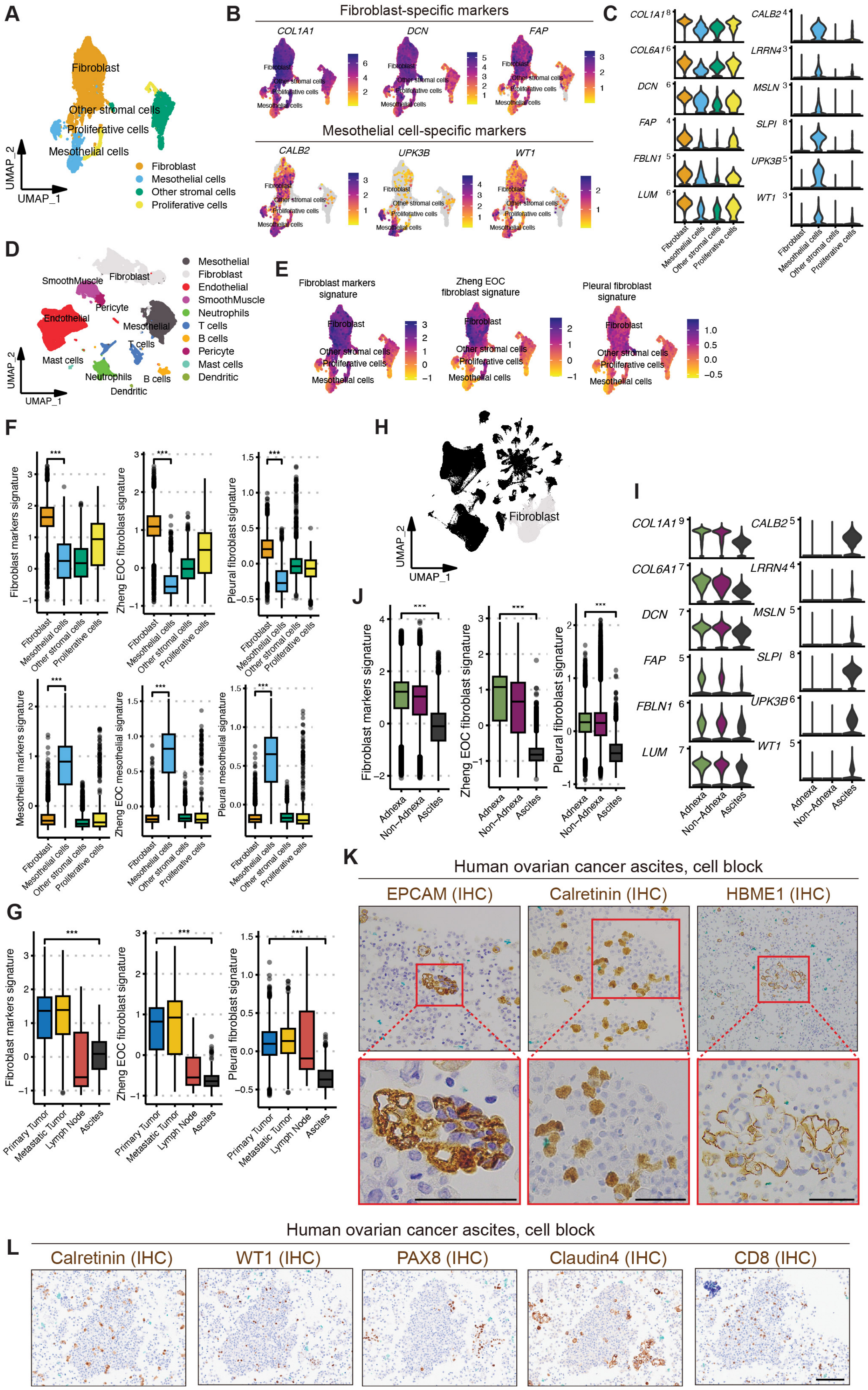
A
Fibroblast
Other stromal cells
Proliferative cells
Mesothelial cells
UMAP_2
UMAP_1
Fibroblast
Mesothelial cells
Other stromal cells
Proliferative cells
B
Fibroblast-specific markers
COL1A1
DCN
FAP
Mesothelial cell-specific markers
CALB2
UPK3B
WT1
C
COL1A1
COL6A1
DCN
FAP
FBLN1
LUM
CALB2
LRRN4
MSLN
SLPI
UPK3B
WT1
D
Mesothelial
Fibroblast
Endothelial
SmoothMuscle
Neutrophils
T cells
B cells
Pericyte
Mast cells
Dendritic
E
Fibroblast markers signature
Zheng EOC fibroblast signature
Pleural fibroblast signature
F
Fibroblast markers signature
Zheng EOC fibroblast signature
Pleural fibroblast signature
Mesothelial markers signature
Zheng EOC mesothelial signature
Pleural mesothelial signature
G
Primary Tumor
Metastatic Tumor
Lymph Node
Ascites
H
Fibroblast
I
J
Adnexa
Non-Adnexa
Ascites
K
Human ovarian cancer ascites, cell block
EPCAM (IHC)
Calretinin (IHC)
HBME1 (IHC)
L
Human ovarian cancer ascites, cell block
Calretinin (IHC)
WT1 (IHC)
PAX8 (IHC)
Claudin4 (IHC)
CD8 (IHC)

**Fig. S3. Stromal cells in ascites showed highly expression of mesothelial signature using three different single-cell RNA-sequencing datasets**. (A) UMAP clustering of the stromal cell compartment colored by the major cell-type clusters exactly as in the original study. (B) UMAP plots showing the RNA expression levels of fibroblast (*COL1A1, DCN*, and *FAP*) and mesothelial cell (*CALB2, UPK3B*, and *WT1*) marker genes. (C) Violin plots showing the expression of prototypical fibroblast and mesothelial marker genes among the stromal cell types specified in this dataset. (D) UMAP reproduction of a publicly available single-cell RNA-sequencing atlas of human parietal pleura. (E) UMAP plots of stromal cells as in (A) showing the expression of three different fibroblast signatures. (F) Box plots showing the expression of the various fibroblast and mesothelial signatures in the different stromal cell types of the Zheng et al EOC dataset. (G) Box plots showing the expression of the three different fibroblast signatures in the Zheng et al EOC dataset (ref 40) depending on tumor location, i.e. primary tumor, metastasis, lymphocyte, and ascites. (H) UMAP as in the original study of an additional EOC single-cell RNA-sequencing dataset and highlighting the cell cluster annotated as the "Fibroblast" cluster. (I) Violin plots showing the expression of fibroblast and mesothelial marker genes depending on tumor location, i.e. adnexa, non-adnexa, and ascites in the "Fibroblast" cluster highlighted in (H). (J) Box plots showing the expression of the three different fibroblast signatures depending on tumor sites. (K) Immunohistochemistry of cell block samples from clinical ascites samples. EPCAM is for EOC cells, and calretinin and HBME1 are for mesothelial cells. Bar = 50 μm. (L) Immunohistochemistry of cell block samples from clinical ascites. Calretinin and WT1 positive cells are negative for PAX8, Claudin-4, and CD8. Bar = 100 μm. *** $p < 0.001$. UMAP, uniform manifold approximation and projection; IHC, immunohistochemistry.

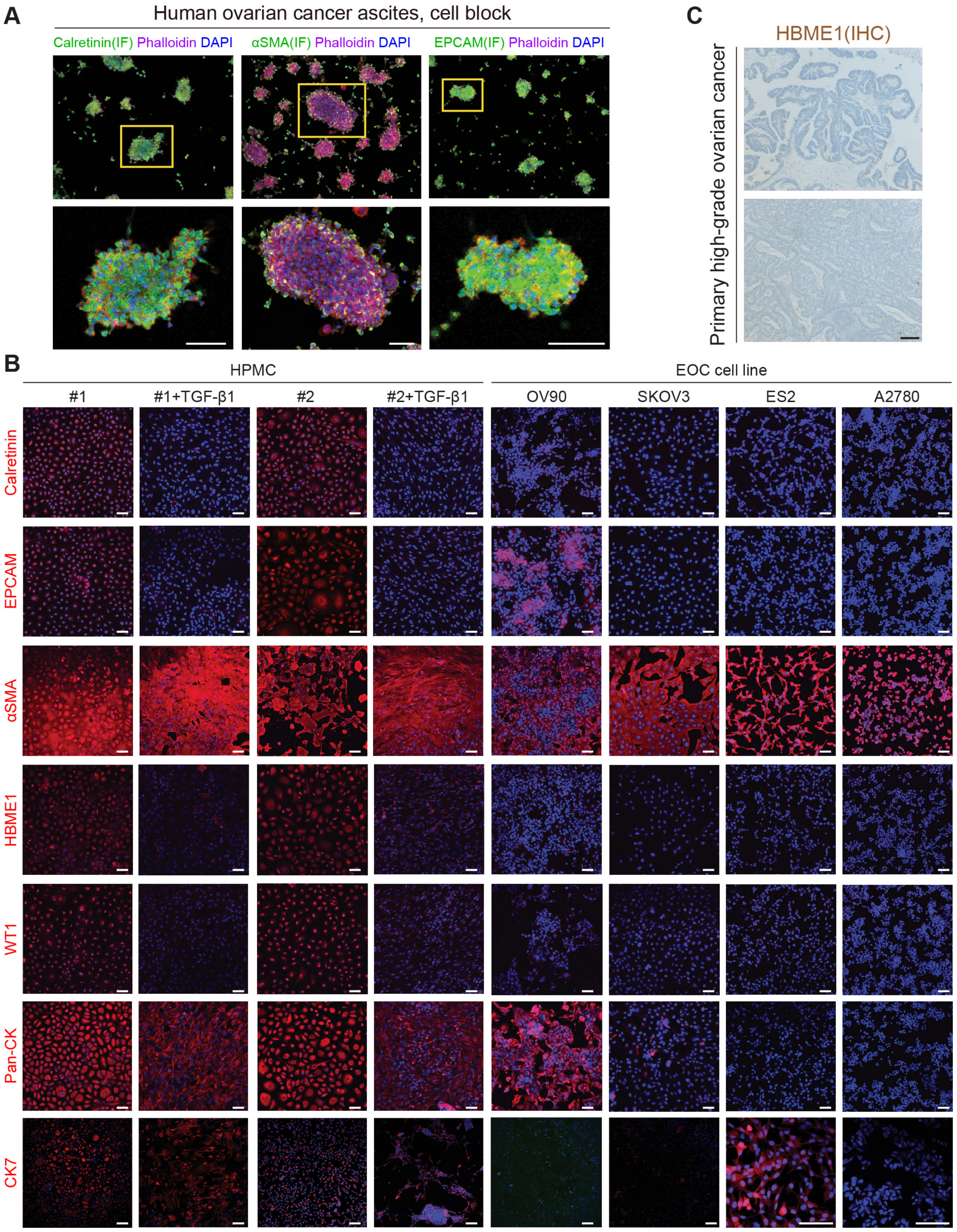
A
Human ovarian cancer ascites, cell block
Calretinin(IF) Phalloidin DAPI
αSMA(IF) Phalloidin DAPI
EPCAM(IF) Phalloidin DAPI
C
HBME1(IHC)
Primary high-grade ovarian cancer
B
HPMC
EOC cell line
#1
#1+TGF-β1
#2
#2+TGF-β1
OV90
SKOV3
ES2
A2780
Calretinin
EPCAM
αSMA
HBME1
WT1
Pan-CK
CK7

**Fig. S4. HBME1 can a suitable marker for detecting mesothelial cells from EOC cells.** (A) Immunofluorescent (IF) images of EOC spheroids with calretinin, αSMA, and EPCAM using confocal microscopy. Although calretinin and αSMA are used to detect mesothelial cells, the EOC cells also showed positive staining. Bar = 50 µm. (B) IF images of mesothelial cells, TGF-β1-stimulated mesothelial cells, and EOC cell line staining. Mesothelial cells showed both epithelial and mesenchymal characteristics. HBME1 can a suitable marker for detecting mesothelial cells from EOC cells. Bar = 50 µm. (C) IHC of primary HGSOC tumor with HBME1. Primary HGSOC tumors were negative staining for HBME1 (n = 14). Bar = 100 µm. EOC, epithelial ovarian cancer; HGSOC, high-grade serous ovarian cancer; IF, immunofluorescent; IHC, immunohistochemistry.

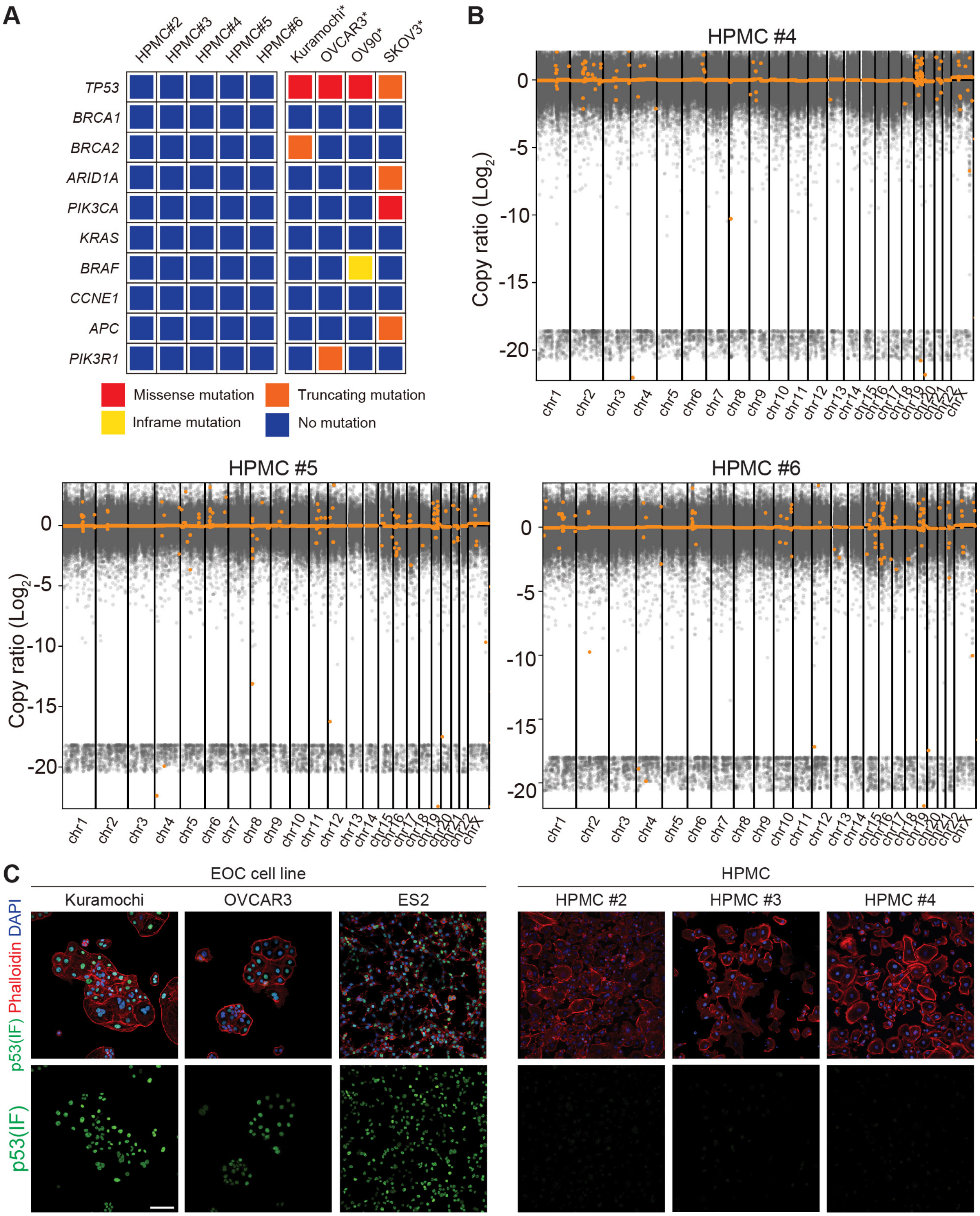


**Fig. S5. Our primary human mesothelial cells (HPMC) had no EOC genetic signature.** (A) Heatmap showing ovarian cancer related genetic mutation. All HPMC had no mutation in these genes. We referred the genetic information of EOC cell lines from ref 46. (B) Scatter plots

showing whole-exome sequencing of HPMC #4, #5, and #6. They had very small number of copy number aberrations, which is very different of EOC cells. (C) Immunofluorescent staining of p53 of EOC cell lines (Kuramochi, OVCAR3, and ES2) and HPMC (#2–#4). All HPMC were negative for p53 staining, while EOC cells were positive for p53 staining. Bar = 100 μm. HPMC, human primary mesothelial cells; EOC, epithelial ovarian cancer.

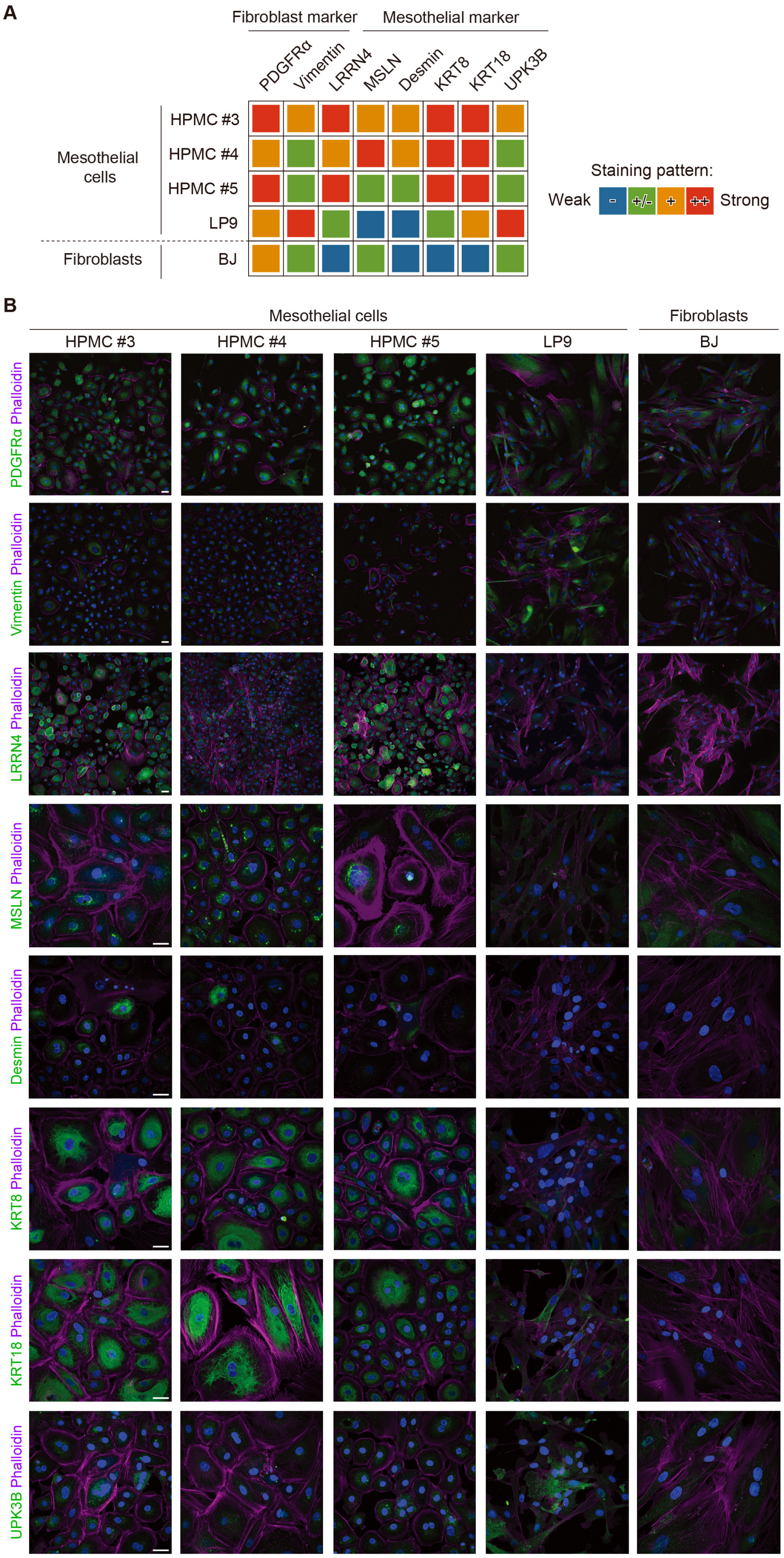
A
Fibroblast marker
Mesothelial marker
PDGFRα
Vimentin
LRRN4
MSLN
Desmin
KRT8
KRT18
UPK3B
Mesothelial cells
HPMC #3
HPMC #4
HPMC #5
LP9
Fibroblasts
BJ
Staining pattern:
Weak
-
+/-
+
++
Strong
B
Mesothelial cells
Fibroblasts
HPMC #3
HPMC #4
HPMC #5
LP9
BJ
PDGFRα Phalloidin
Vimentin Phalloidin
LRRN4 Phalloidin
MSLN Phalloidin
Desmin Phalloidin
KRT8 Phalloidin
KRT18 Phalloidin
UPK3B Phalloidin

**Fig.S6. Our human primary mesothelial cells (HPMC) showed similar character with single-cell RNA-sequencing datasets.** (A) Heatmap showing summary of immunofluorescent staining with mesothelial and fibroblast markers in three kinds of HPMC, mesothelial cell line, LP9, and fibroblast cell line, BJ. (B) Representative immunofluorescent images of each staining in each cell. Green shows specific protein. Magenta represents phalloidin and blue represents DAPI. Bar = 30 μm.

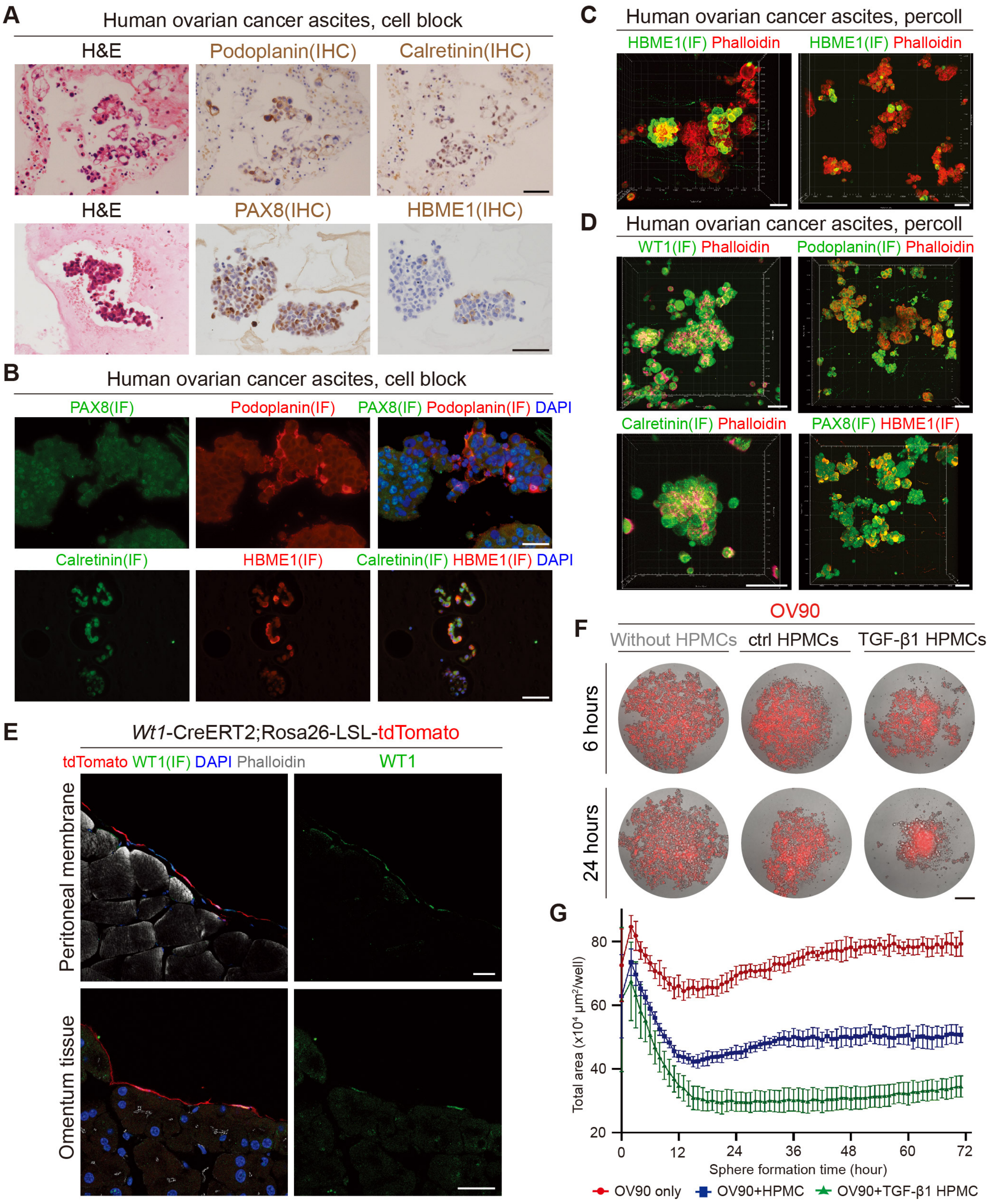


**Fig. S7. Detection of mesothelial cells in EOC spheroids**. (A) Immunohistochemistry cell block samples with H&E, and podoplanin/calretinin or PAX8/HBME1. Several cells in cancer spheroids were positive for mesothelial marker staining. Bar = 100 μm. (B) Double stained immunofluorescence cell block samples with PAX8 (for EOC cells) and

calretinin/HBME1/podoplanin (for mesothelial cells). Bar = 50 µm. (C) Spheroids were observed using a multiphoton microscope. Several spheroids included HBME1-positive mesothelial cells, while some spheroids were entirely negative for HBME1 staining. Bar = 50 µm. (D) Representative images of EOC spheroids with various stains (green) obtained using multiphoton microscopy. Calretinin and WT1 were positive for both EOC and mesothelial cells. Bar = 50 µm. (E) Peritoneal membrane and omentum tissues 4 weeks after tamoxifen induction. Only the peritoneal membrane was positive for Tomato. These cells were also positive for WT1 staining. Bar = 30 µm. (F, G) Spheroid formation with different incubation times (6 h and 24 h) and sphere-forming curve analysis for (i) OV90 only, (ii) with HPMC, and (iii) with HPMC with TGF-β1 stimulation. Bar = 200 µm. EOC, epithelial ovarian cancer; HPMC, human primary mesothelial cells.

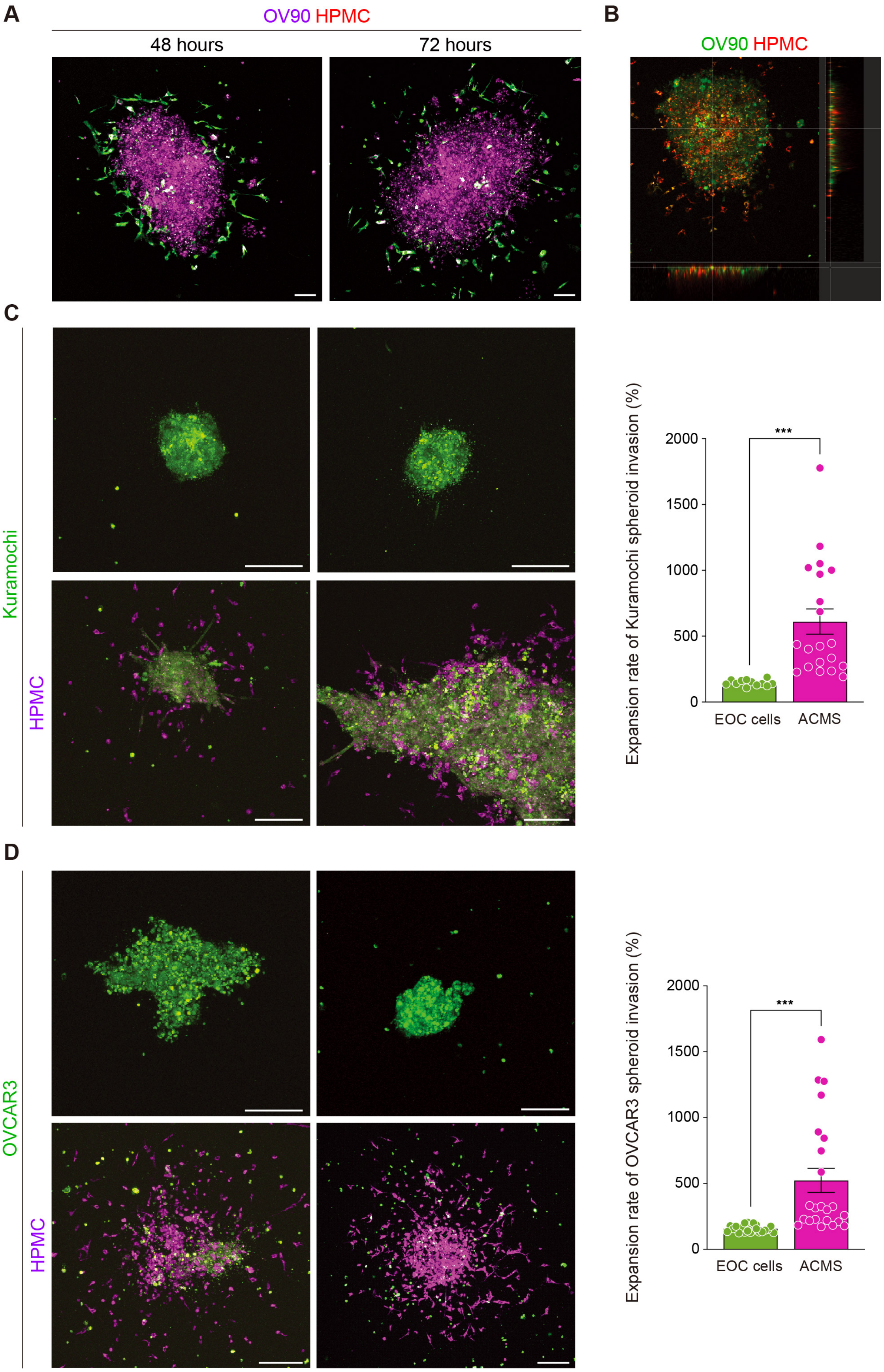
A
OV90 HPMC
48 hours
72 hours
B
OV90 HPMC
C
Kuramochi
HPMC
Expansion rate of Kuramochi spheroid invasion (%)
2000
1500
1000
500
0
***
EOC cells
ACMS
D
OVCAR3
HPMC
Expansion rate of OVCAR3 spheroid invasion (%)
2000
1500
1000
500
0
***
EOC cells
ACMS

**Fig. S8. Mesothelial cells invade first collagen layer from ACMS regardless of EOC cell lines.** (A) Representative images of ACMS invading collagen. Green-stained mesothelial cells invaded the collagen layer, while most red-stained OV90 cells stayed inside the ACMS. Bar = 100 μm. (B) 3D Images of the cell invasion showing the vertical and horizonal directions. Red-stained mesothelial cells invaded the collagen more than EOC cells in each field. (C, D) Representative images of collagen invasion from a spheroid and bar graphs showing expansion rate of spheroid invasion from original spheroids at 72 h. Spheroids were formed with red-stained mesothelial cells and green-stained (C) Kuramochi, and (D) OVCAR3. Bar = 200 μm. *** $p < 0.001$.

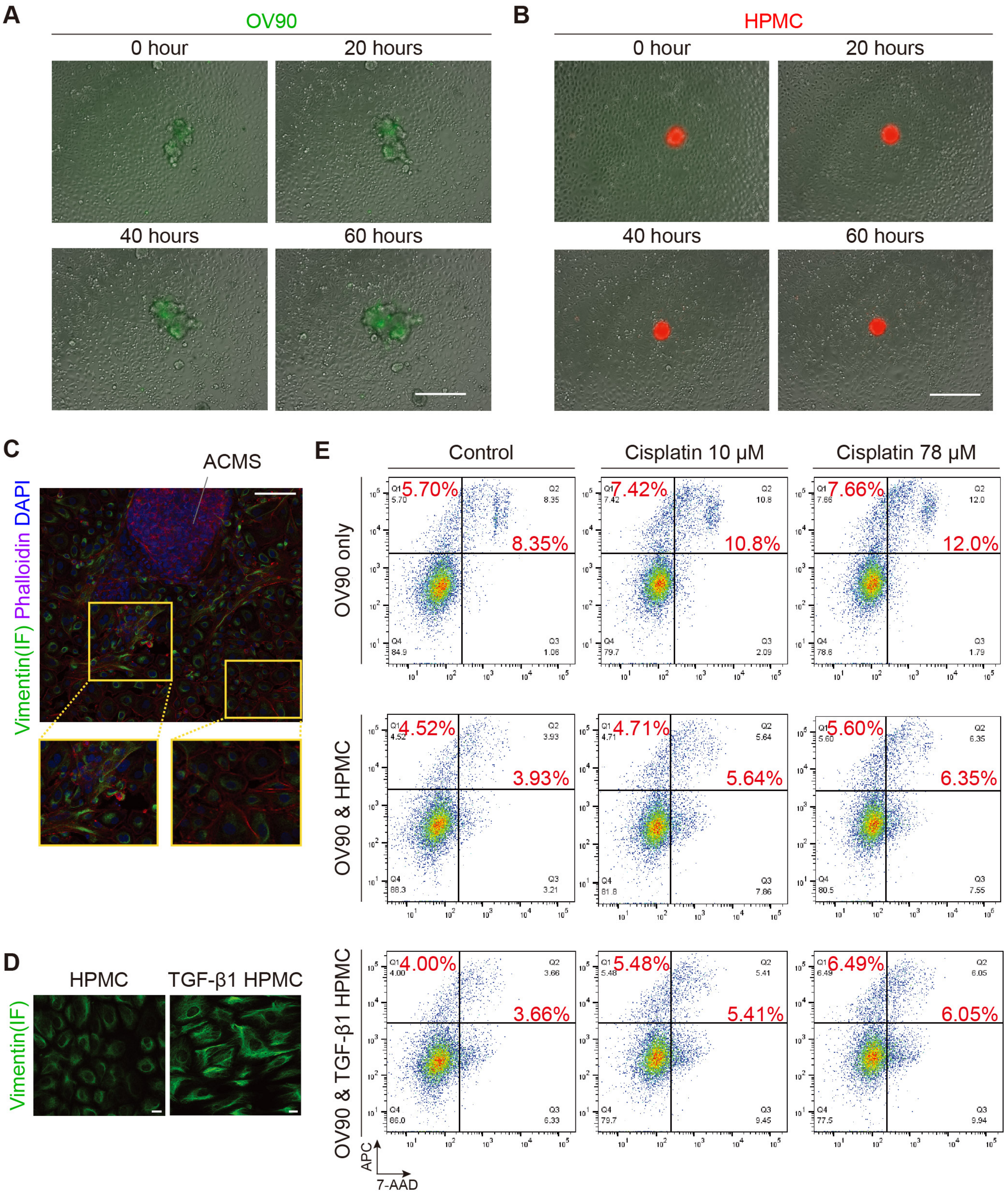


**Fig. S9. ACMS has an aggressive mesothelial clearance capability and resistance to anoikis and chemotherapy.** (A, B) Representative images of spheroids with only (A) OV90 (stained with green) or (B) HPMC (stained with red) invading into the mesothelial layer at 0 h, 20 h, 40 h, and 60 h. Bar = 200 μm. (C) Representative images of ACMS invading the mesothelial layer. Mesothelial cells showing spindle mesenchymal morphology close to the invasion border were strongly positive staining for vimentin, while mesothelial cells showing cobblestone appearance

far from invasion border were almost negative. Bar = 100 μm. (D) Immunofluorescent images of vimentin in HPMC with and without TGF-β1 stimulation. Bar = 15 μm. (E) Representative data of the apoptosis assay for OV90 cells. The percentage represents the rate of cells that are positive for both 7-AAD and APC. The rate of apoptosis cells in spheroids with only OV90 was higher than that of spheroids with mesothelial cells (8.3% vs. 3.6%). The rates of apoptotic cells were also higher in spheroids with only OV90 cells when these spheroids were treated with different concentration of cisplatin. HPMC, human primary mesothelial cells; ACMS, aggregated cancer-mesothelial spheroid.

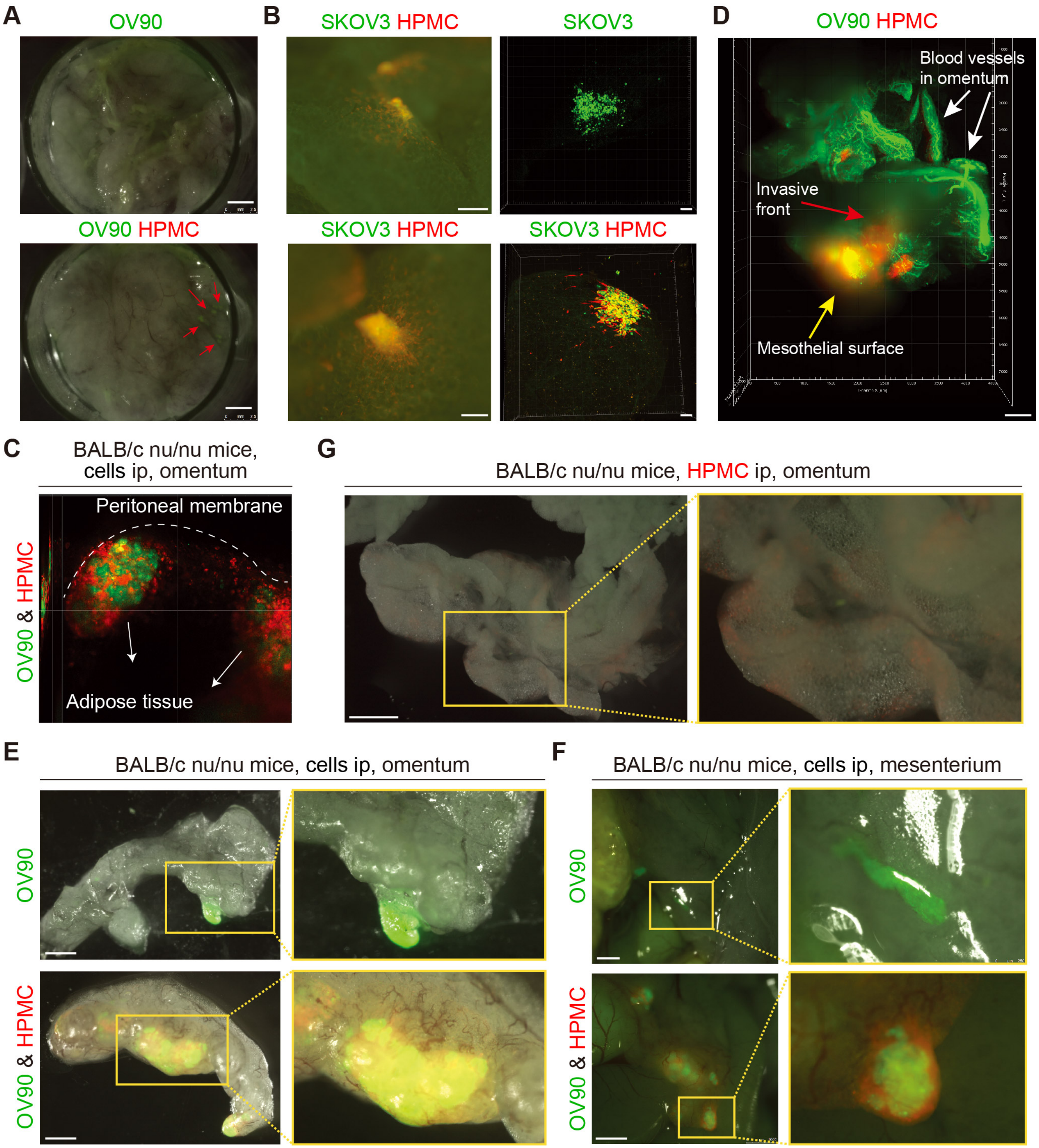


**Fig. S10. Mesothelial cells within ACMS invaded adipose tissue first and induced peritoneal metastases *ex vivo* and *in vivo*.** (A) Images of Metastases on omentum. Bar = 2 mm. (B) Representative images obtained using a fluorescent microscope and confocal microscope. On the outer rim of metastases, red-stained mesothelial cells invaded the omentum, while green-stained SKOV3 cells remained close to the metastasis centers. Bar = 200 µm. (C) Multiphoton microscopy image of the omentum in the early phase of invasion. Red-stained mesothelial cells invaded into deeper adipose tissues, while green-stained OV90 cells remained in the center of the metastasis site. (D) Representative images showing the decolorization method. The yellow arrow

denotes the metastasis sites on the surface of the omentum. The red arrow indicates the invasive front of this region. Only red-stained mesothelial cells invaded the deep adipose tissue. Green tube structures (white arrows) represent the original blood vessels in the omentum. Bar = 500 µm. (E) Representative images showing the omentum after spheroid injection. While small amounts of metastasis were observed when mice were injected with spheroids with only OV90, large metastases were observed when mice were injected with ACMS. Red-stained mesothelial cells surrounded metastasis sites, as shown in Figures 4I and 4J. Bar = 2.5 mm. (F) Representative images of metastases on mescenterium. Metastasis pattern was the same with the metastases on omentum. Bar = 1.0 mm. (G) When only red-stained mesothelial cells were injected, they attached to the surface of the omentum but did not invade or grow. Bar = 1.0 mm. ACMS, aggregated cancer-mesothelial spheroid. HPMC, human primary mesothelial cells.

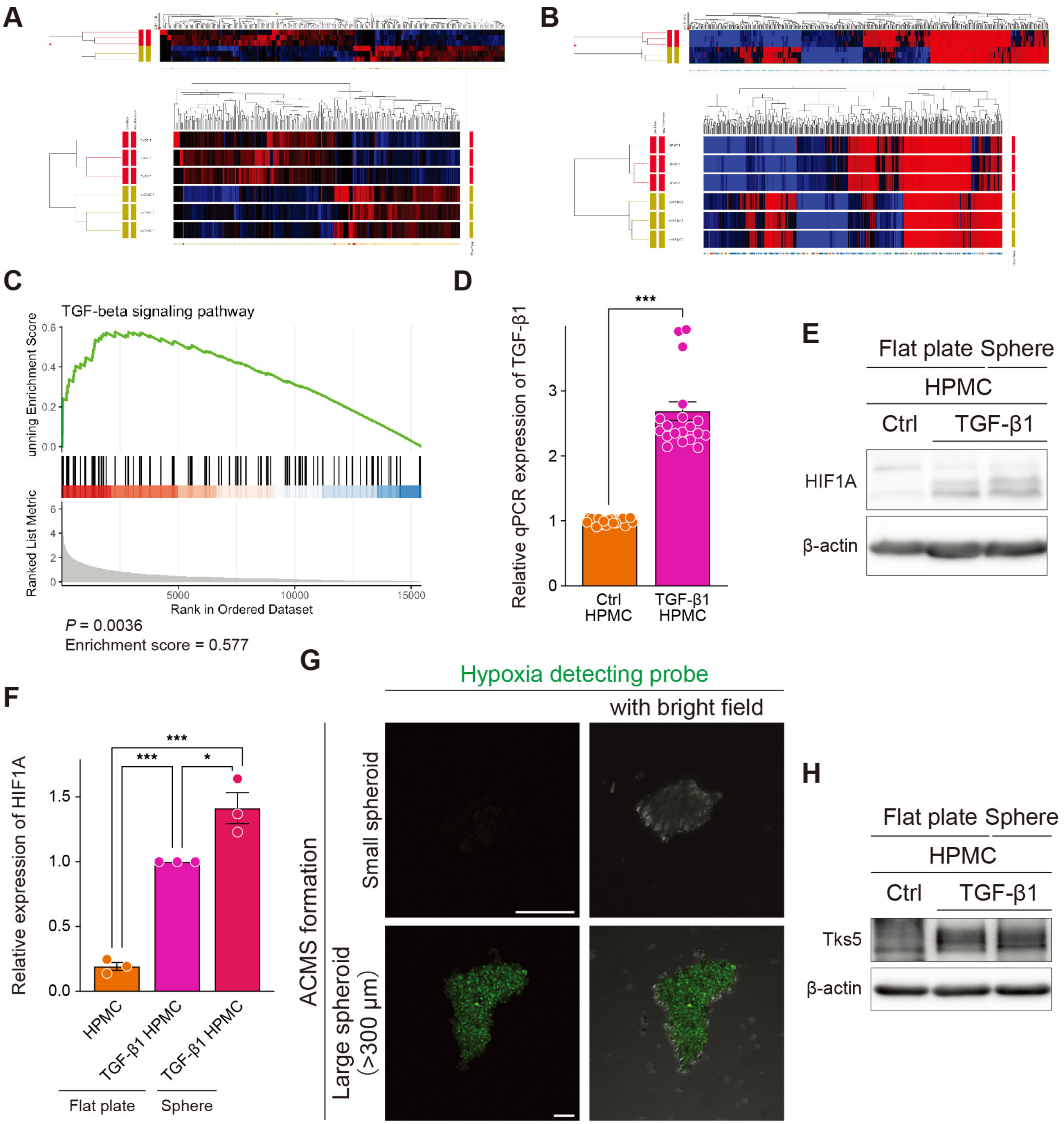


**Fig. S11. EOC cells change the RNA expression in mesothelial cells via spheroid interactions.** (A, B) Clustering of genetic expression changes in (A) OV90 and (B) HPMC. (C) The TGF-β pathway was significantly upregulated in mesothelial cells after forming ACMS. (D) Box plot showing relative qPCR expression of TGF-β1 in mesothelial cells with and without TGF-β1 stimulation. *** $p < 0.001$. (E, F) Western blot analysis of HIF1A. The expression was significantly higher in TGF-β1-stimulated mesothelial cells compared to the control cells. Moreover, it was further high with spheroid formation. * $p < 0.05$, *** $p < 0.001$. (G) Representative images of spheroids with the hypoxia detecting probe. Bar = 100 μm. (H) Western blotting revealed increased Tks5 levels in TGF-β1-stimulated mesothelial cells. The expression level of Tks5 was upregulated during sphere formation. ACMS, aggregated cancer-mesothelial spheroid.

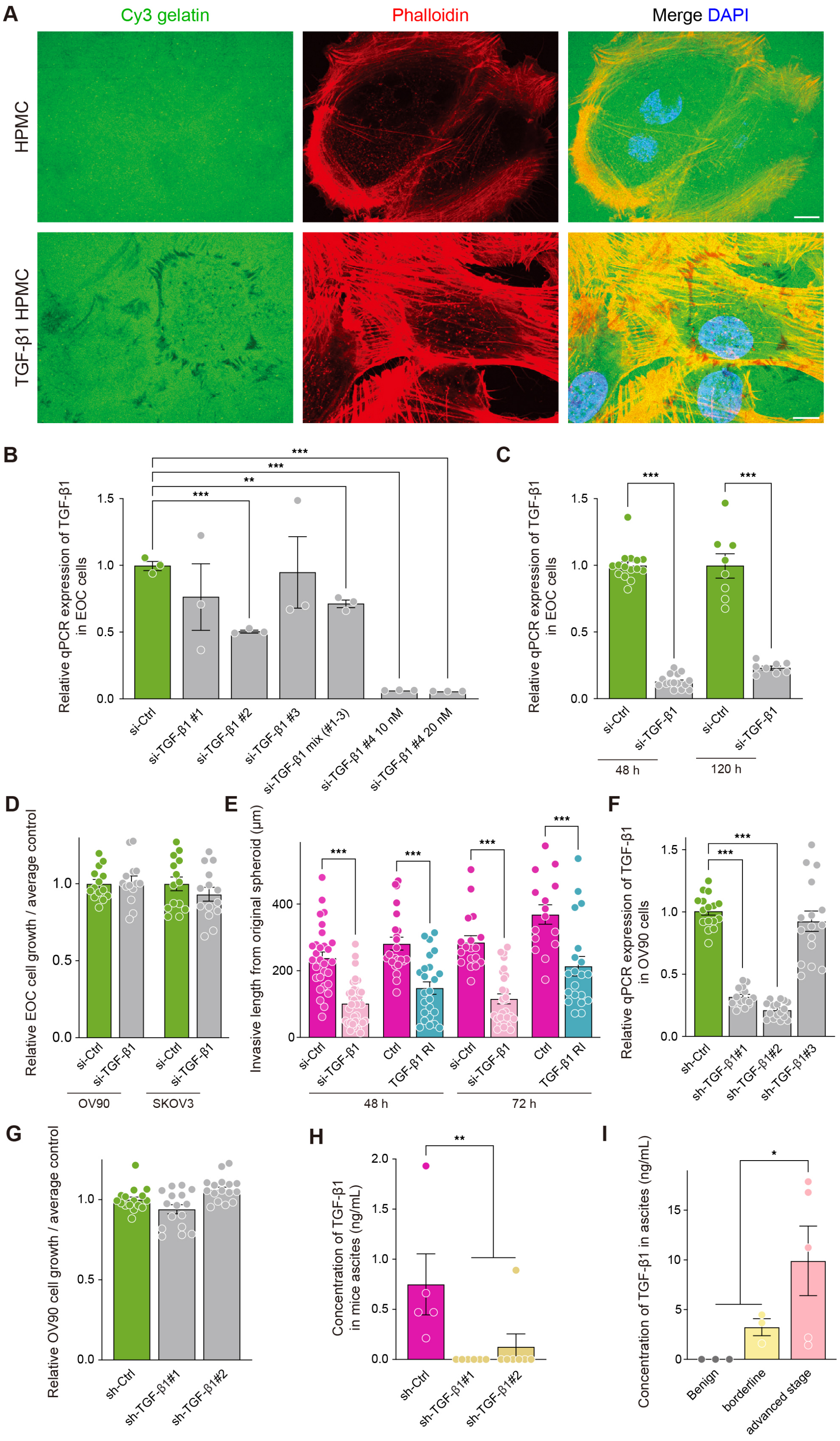
A
Cy3 gelatin
Phalloidin
Merge DAPI
HPMC
TGF-β1 HPMC
B
Relative qPCR expression of TGF-β1 in EOC cells
si-Ctrl
si-TGF-β1 #1
si-TGF-β1 #2
si-TGF-β1 #3
si-TGF-β1 mix (#1-3)
si-TGF-β1 #4 10 nM
si-TGF-β1 #4 20 nM
C
Relative qPCR expression of TGF-β1 in EOC cells
si-Ctrl
si-TGF-β1
48 h
120 h
D
Relative EOC cell growth / average control
OV90
SKOV3
E
Invasive length from original spheroid (μm)
Ctrl
TGF-β1 RI
48 h
72 h
F
Relative qPCR expression of TGF-β1 in OV90 cells
sh-Ctrl
sh-TGF-β1#1
sh-TGF-β1#2
sh-TGF-β1#3
G
Relative OV90 cell growth / average control
H
Concentration of TGF-β1 in mice ascites (ng/mL)
I
Concentration of TGF-β1 in ascites (ng/mL)
Benign
borderline
advanced stage

**Fig. S12. TGF-β1 stimulation from EOC cells transforms mesothelial cells into highly invasive phenotype.** (A) The Cy3 gelatin assay of the mesothelial cells and TGF-β1-stimulated mesothelial cells. TGF-β1-stimulated mesothelial cells degraded more gelatin than the control cells. Bar = 10 μm. (B) Bar graph showing relative qPCR expression of TGF-β1 with siRNA for TGF-β1 (#1–4) for 48 h. si-TGF-β1 #4 showed the strongest inhibitory effect and was selected for subsequent experiments (C) Bar graph showing relative qPCR expression of TGF-β1 in EOC cells when inhibiting TGF-β1 with si-RNA at day 2 and day 5 after siRNA treatment. (D) Bar graph showing growth ability of EOC cells when inhibiting TGF-β1 with si-RNA. (E) Bar graph showing invasive length from original spheroids to invasive front when EOC cells were inhibited si-RNA for TGF-β1 or mesothelial cells were treated with TGF-β1 receptor inhibitor at 48 h and 72 h. (F) Bar graph showing relative qPCR expression of sh-Control- or TGF-β1 (#1–3)-transduced OV90 cells. Sh-TGF-β1 #1 and #2 showed the strong inhibitory effect. (G) Bar graph showing growth rate of sh-TGF-β1-transduced OV90 cells on day 3. (H) Bar graph showing the TGF-β1 concentration in ascites was significantly lower in mice receiving sh-TGF-β1-transduced OV90 cells than in control mice (n=6). (I) Bar plot showing the concentration of TGF-β1 in ascites from the patients with benign (n = 3), borderline/early stage (n = 3), and advanced stage (n = 5) EOC. More than half of the ascites in the advanced stage included >10 ng/mL TGF-β1 in their ascites. * $p < 0.05$, ** $p < 0.01$, *** $p < 0.001$. HPMC, human primary mesothelial cells.

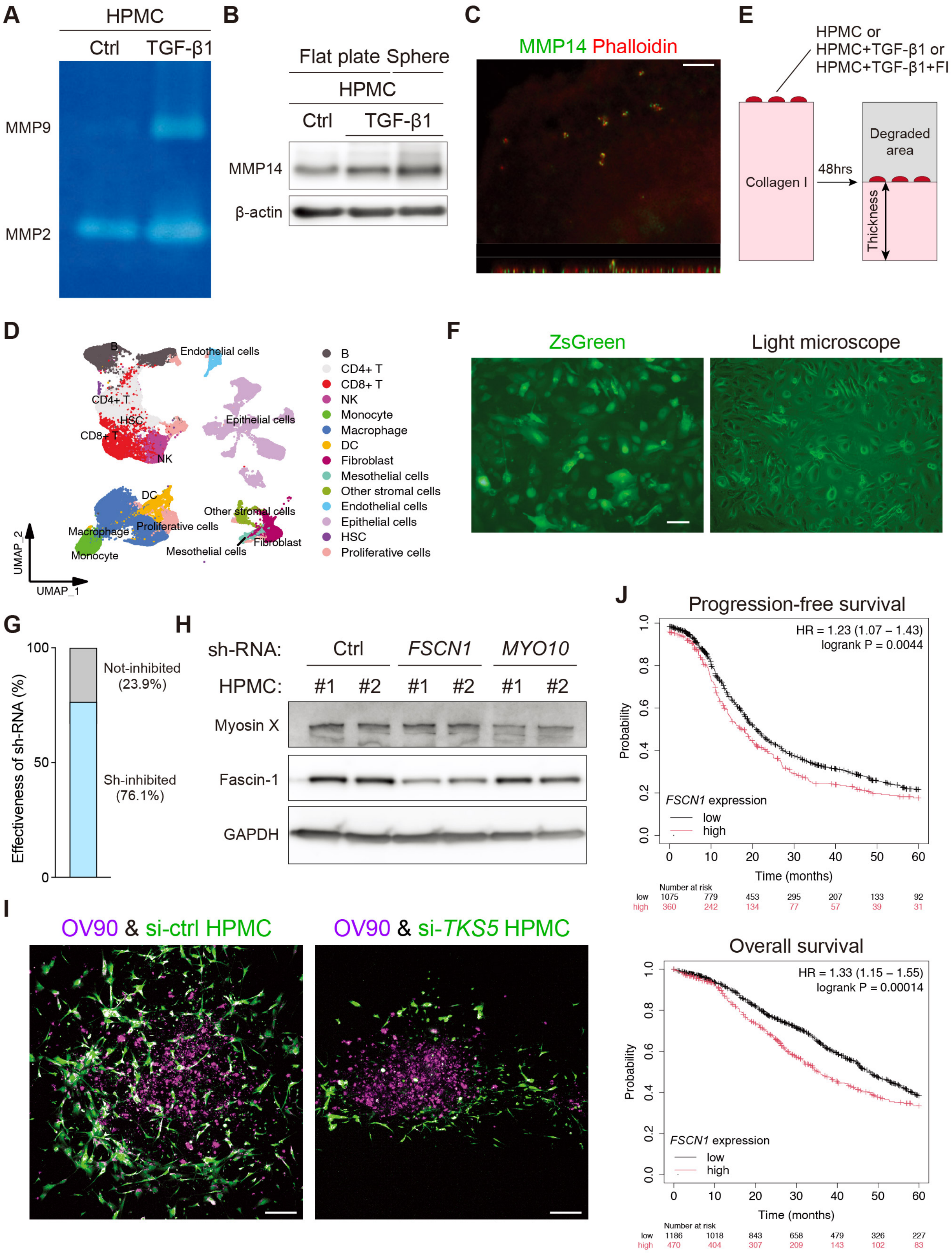

A
HPMC
Ctrl
TGF-β1
MMP9
MMP2
B
Flat plate Sphere
HPMC
Ctrl
TGF-β1
MMP14
β-actin
C
MMP14 Phalloidin
E
HPMC or
HPMC+TGF-β1 or
HPMC+TGF-β1+FI
Collagen I
48hrs
Degraded area
Thickness
D
B
Endothelial cells
CD4+ T
HSC
CD8+ T
NK
Epithelial cells
DC
Other stromal cells
Proliferative cells
Macrophage
Monocyte
Mesothelial cells
Fibroblast
UMAP_2
UMAP_1
B
CD4+ T
CD8+ T
NK
Monocyte
Macrophage
DC
Fibroblast
Mesothelial cells
Other stromal cells
Endothelial cells
Epithelial cells
HSC
Proliferative cells
F
ZsGreen
Light microscope
G
Effectiveness of sh-RNA (%)
100
50
0
Not-inhibited (23.9%)
Sh-inhibited (76.1%)
H
sh-RNA:
Ctrl
FSCN1
MYO10
HPMC:
#1 #2 #1 #2 #1 #2
Myosin X
Fascin-1
GAPDH
I
OV90 & si-ctrl HPMC
OV90 & si-TKS5 HPMC
J
Progression-free survival
HR = 1.23 (1.07 – 1.43)
logrank P = 0.0044
Probability
FSCN1 expression
low
high
Time (months)
Number at risk
low 1075 779 453 295 207 133 92
high 360 242 134 77 57 39 31
Overall survival
HR = 1.33 (1.15 – 1.55)
logrank P = 0.00014
Probability
FSCN1 expression
low
high
Time (months)
Number at risk
low 1186 1018 843 658 479 326 227
high 470 404 307 209 143 102 83

**Fig. S13. TGF-β1-stimulated mesothelial cells secreted MMP2/9 and activated at the invadopodia by MMP14.** (A) Gelatin zymography shows that the concentrations of MMP2 and MMP9 were increased in the supernatant of TGF-β1-stimulated mesothelial cells compared to the control cells. (B) The MMP14 expression was increased when mesothelial cells stimulated TGF-β1 and formed spheroids. (C) Immunofluorescent 3D images of MMP14 (green) and phalloidin (red) in TGF-β1-stimulated mesothelial cells. Phalloidin location represents invadopodia formation. MMP14 is detected in the invadopodia region. Bar = 4 μm. (D) UMAP reproduction of a publicly available single-cell RNA-sequencing dataset from Zheng et al (ref. 40). (E) Schematic illustration of the collagen degradation assay. (F, G) For shRNA expressing experiment, mesothelial cells were infected with an AAV carrying an shRNA-expressing vector co-expressed with the ZsGreen fluorescence protein. More than 75% of mesothelial cells were ZsGreen-positive. Bar = 50 μm. (H) Western blotting analyses revealed that sh-fascin1 and -myosin X inhibited the expression of fascin1 and myosin X, respectively. (I) Representative images of collagen invasion of ACMS. The invasive ability of mesothelial cells was inhibited when Tks5 was inhibited in mesothelial cells. (J) Kaplan–Meier analysis revealed the expression of *FSCN1* was related to PFS (P = 0.0044) and OS (P = 0.00014) in patients with ovarian cancer. MMP, matrix metalloproteinase; UMAP, uniform manifold approximation and projection; PFS, progression-free survival; OS, overall survival.

**Table S1.**

Supplementary Table Patient backgrounds

| Characteristics | All patients (n = 983) |
|---|---|
| **Age in years, median ± SD (range)** | 56 ± 12.5 (19–92) |
| **FIGO tumor stage** | |
| I, n (%) | 303 (30.8%) |
| II, n (%) | 114 (11.6%) |
| III, n (%) | 475 (48.3%) |
| IV, n (%) | 91 (9.3%) |
| **Pathology** | |
| High-grade serous carcinoma, n (%) | 628 (63.9%) |
| Endometrioid carcinoma, n (%) | 355 (36.1%) |
| **CA125 level, U/mL, median ± SD (range)** | 539.2 ± 4,122.5 (2–60,000) |
| **Positive cytology, n (%)** | 544 (55.3%) |
| **Complete resection, n (%)** | 293 (29.8%) |
| **Recurrence, n (%)** | 546 (55.5%) |
| **Progression-free interval, day, median ± SD (range)** | 841 ± 1,268 (0–5,630) |
| **Death, n (%)** | 394 (40.1%) |
| **Overall survival, day, median ± SD (range)** | 1402 ± 1,279 (10–7,038) |

SD, standard deviation; FIGO, International Federation of Gynecology and Obstetrics; CA, cancer antigen.

**Captions for the supplementary movie files**

**Movie. S1. 3D image of a whole ovarian cancer spheroids.** The multiphoton microscope could observe whole spheroids and HBME1 clearly distinguished mesothelial cells within ovarian cancer spheroids.

**Movie. S2. 3D image of a whole ovarian cancer spheroids after chemotherapy.** Some spheroids observed after chemotherapy showed cancer cells located within HBME1-positive cells.

**Movie. S3. Time-lapse imaging of forming a spheroid with only OV90 cells.** Spheroids with only OV90 cells were less aggregate and easily broken.

**Movie. S4. Time-lapse imaging of forming aggregated cancer-mesothelial spheroids (ACMS).** Spheroids with OV90 and mesothelial cells formed aggregated and strongly connected spheroids.

**Movie. S5. Time-lapse imaging of ACMS invasion into the mesothelial layer.** Spheroids gradually invaded mesothelial layer and induced metastatic regions with four steps.

**Movie. S6. Time-lapse imaging of spheroids with only OV90 cells invasion.** It demonstrated longer duration before invading the mesothelial layer.

**Movie. S7. Time-lapse imaging of spheroids with only mesothelial cells invasion.** The spheroid remained localized in the same position.

**Movie. S8. 3D image of omentum metastasis with** tissue decolorization. Both green-stained OV90 cells and red-stained mesothelial cells were present on the surface of the omentum. In contrast, only mesothelial cells were present in the deeper adipose regions.

**Captions for the supplementary supportive data**

**Supportive Data S1. Raw data used to generate the main and supplementary figures.** Each tab contains corresponding data for figure preparation, and the tab names indicate the associated figure panels.
